# Harnessing coumarin privilege to achieve potency and selectivity: hypoxia-targeted alkyl coumarin-tethered phenyl quinazolinones as dual hCA IX/HSP90α inhibitors with chemosensitizing activity

**DOI:** 10.1039/d6ra06511b

**Published:** 2026-09-09

**Authors:** Amal S. Elgohary, Mervat H. El-Hamamsy, Nabaweya Sharafeldin, Simone Giovannuzzi, Triantafyllos-Dimitrios Gerokonstantis, Nikolaos S. Thomaidis, Claudiu T. Supuran, Haytham O. Tawfik

**Affiliations:** a Department of Pharmaceutical Chemistry, Faculty of Pharmacy, Tanta University Tanta 31527 Egypt haytham.omar.mahmoud@pharm.tanta.edu.eg; b Department of NEUROFARBA, Section of Pharmaceutical and Nutraceutical Sciences, University of Florence, Polo Scientifico Via U. Schiff 6 50019 Sesto Fiorentino Firenze Italy claudiu.supuran@unifi.it; c Laboratory of Analytical Chemistry, Department of Chemistry, National and Kapodistrian University of Athens, Panepistimiopolis Zografou 15771 Athens Greece; d Department of Pharmaceutical Chemistry, Faculty of Pharmacy, Alsalam University Tanta 31611 Egypt

## Abstract

Hypoxic tumor regions remain challenging to treat in breast cancer therapy due to extracellular acidification, chemoresistance, and other adaptive mechanisms promoted by the coordinated actions of tumor-associated carbonic anhydrase IX (hCA IX), heat shock protein 90 alpha (HSP90α), and other proteins. Inspired by the privileged role of coumarins as selective hCA IX inhibitors and as HSP90α modulators, together with the ATP-mimetic and anticancer properties of phenyl quinazolinones, a series of alkyl coumarin-tethered phenyl quinazolinones (4a–l) were rationally designed and synthesized as hypoxia-targeted dual hCA IX/HSP90α inhibitors. ^1^H NMR, ^13^C NMR-DEPTQ, HRMS, elemental analysis, and HPLC unequivocally established the structures of the synthesized compounds. The synthesized hybrids exhibited potent, highly selective inhibition of hCA IX (in the nanomolar range), while displaying negligible activity against the off-target isoforms hCA I and II. Compound 4b emerged as the lead derivative, exhibiting a *K*_I_ value of 51.7 nM against hCA IX together with exceptional selectivity (>1934-fold over hCA I and hCA II). Remarkably, 4b also exhibited potent HSP90α inhibitory activity (IC_50_ = 13.21 nM), comparable to that of ganetespib. Furthermore, 4b preferentially suppressed the proliferation of hypoxic MCF-7 and MDA-MB-231 breast cancer cells, sensitized them to doxorubicin, and induced p53-mediated mitochondrial apoptosis. Molecular docking and molecular dynamics simulations against hCA IX and HSP90α, along with *in silico* ADME and toxicity studies, further supported the favorable binding behavior, stability, and druglike characteristics of the lead compound.

## Abstract 2

Hypoxic breast tumors are difficult to treat due to extracellular acidification and chemoresistance driven by tumor-associated carbonic anhydrase IX (hCA IX), HSP90α, and related proteins.

## Graphical abstract

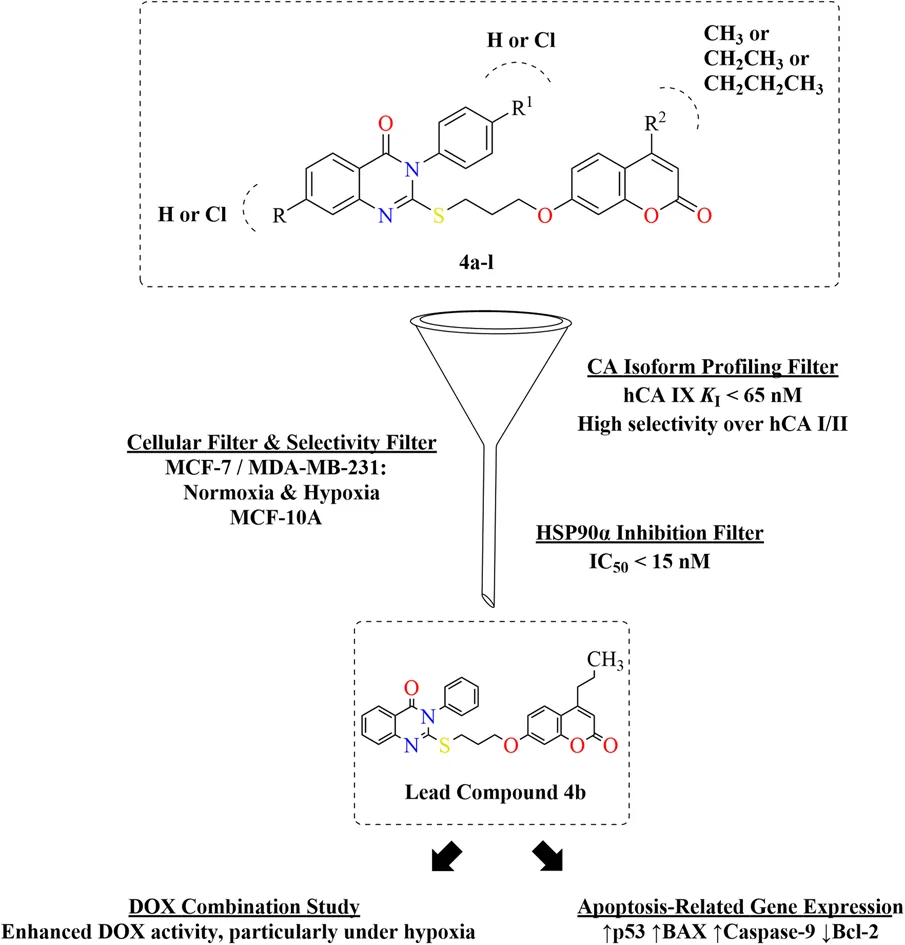

## Introduction

Breast cancer remains the most frequently diagnosed malignancy and one of the leading causes of cancer-related mortality among women worldwide.^1^ Despite substantial advances in diagnosis and targeted therapies, therapeutic resistance remains a major clinical challenge, particularly in aggressive subtypes.^2^ Tumor hypoxia, a hallmark of the breast tumor microenvironment, promotes extracellular acidification, metabolic reprogramming, invasion, metastasis, and resistance to chemotherapy.^3^ These adaptive responses are largely orchestrated by hypoxia-inducible factor-1 alpha (HIF-1α), a master transcriptional regulator of tumor survival and progression.^4^ Consequently, exploiting hypoxia-associated therapeutic vulnerabilities has emerged as an attractive strategy for the development of novel anticancer agents capable of overcoming breast cancer chemoresistance.^5^

Among the numerous HIF-1α-regulated proteins implicated in hypoxic adaptation, tumor-associated carbonic anhydrase IX (hCA IX) has attracted considerable attention owing to its restricted expression in normal tissues and pronounced overexpression in hypoxic solid tumors, including breast cancer.^6^ By regulating extracellular acidification and intracellular pH homeostasis, hCA IX promotes tumor survival, invasion, and chemoresistance, making it an attractive therapeutic target.^7^ Although sulfonamides remain the most extensively investigated hCA inhibitors, coumarins have emerged as a distinct class of non-sulfonamide inhibitors exhibiting remarkable isoform selectivity. Notably, the naturally occurring sesquiterpene coumarin, *e.g.*, colladonin, displayed potent inhibitory activity against hCA IX (*K*_I_ = 90.0 nM) and hCA XII (*K*_I_ = 210.0 nM), while exhibiting substantially weaker activity against hCA I and II.^8^ These findings highlight coumarins as privileged scaffolds for the development of selective hypoxia-associated carbonic anhydrase inhibitors (Fig. 1).

**Fig. 1:**
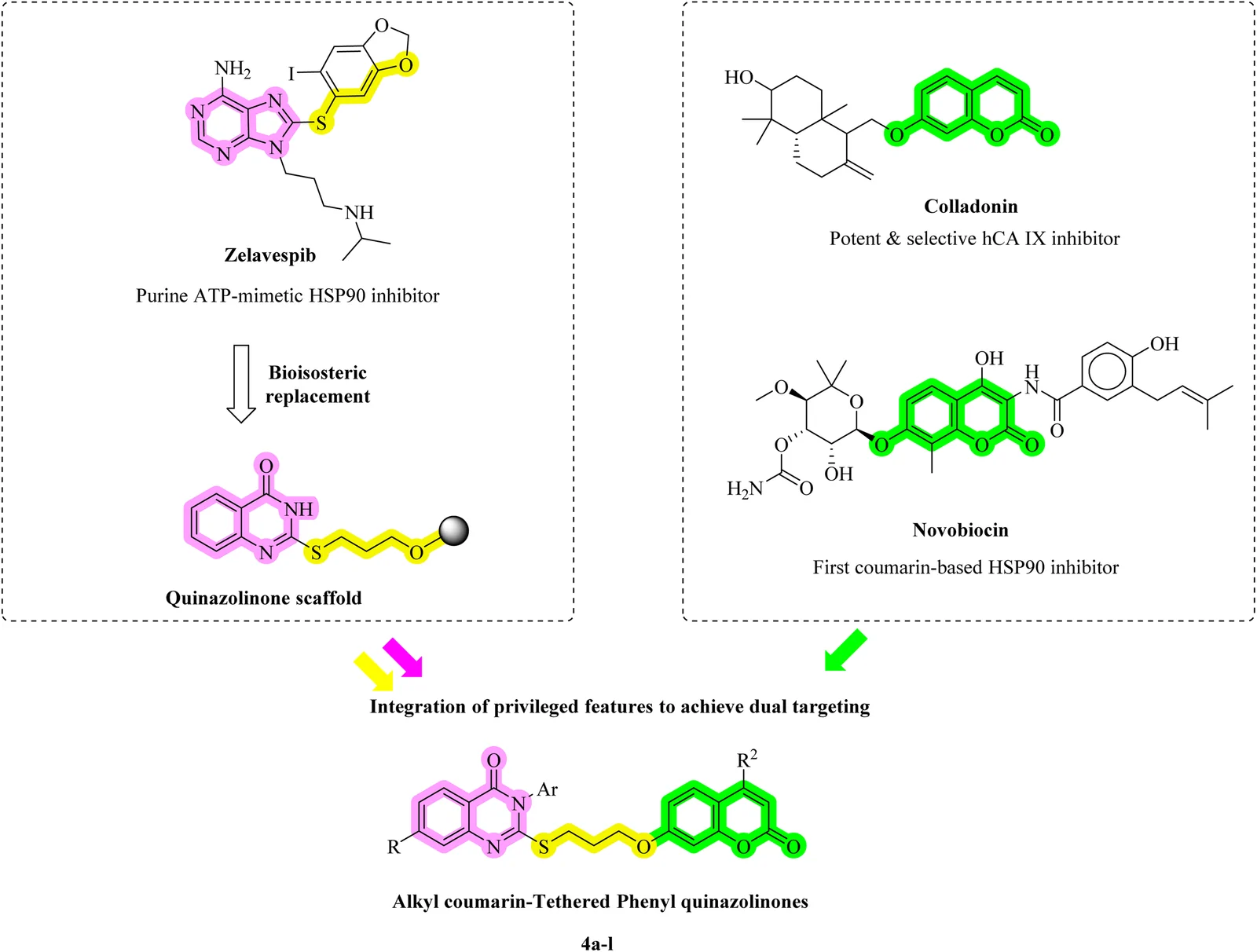
Rational design of alkyl coumarin-tethered phenyl quinazolinones (4a–l) through integration of coumarin privilege and quinazolinone ATP-mimetic features as dual hCA IX/HSP90α inhibitors.

In addition to hCA IX, heat shock protein 90 alpha (HSP90α) has emerged as another attractive therapeutic target in hypoxic tumors owing to its central role in maintaining the stability of numerous oncogenic client proteins, including HIF-1α, thereby promoting tumor survival and adaptation to hypoxic stress.^9–12^ Interestingly, coumarins have attracted considerable attention as HSP90 modulators, as exemplified by novobiocin and its analogues, which established the coumarin scaffold as a privileged motif for HSP90 inhibition.^13–15^ On the other hand, several ATP-mimetic purine derivatives, such as zelavespib (PU-H71), have demonstrated potent HSP90 inhibitory activity through occupation of the ATP-binding pocket.^16–20^ Inspired by these findings, we envisioned that replacing the purine nucleus with a quinazolinone moiety, a privileged anticancer heterocycle possessing ATP-mimetic characteristics, could furnish novel dual hCA IX/HSP90α inhibitors with enhanced anticancer activity (Fig. 1).

In the present series, the linker connecting the quinazolinone and coumarin pharmacophores was deliberately kept constant to preserve a uniform spatial relationship between the two privileged scaffolds, while the SAR investigation focused on variations in the quinazolinone substitution pattern and the alkyl substituent on the coumarin moiety. Therefore, the current dataset does not permit a direct assessment of the effect of linker length on biological activity.

Collectively, these observations suggest that hypoxia simultaneously drives hCA IX overexpression and increases tumor dependence on HSP90α, thereby promoting extracellular acidification, survival signaling, and chemoresistance. Therefore, concurrent inhibition of hCA IX and HSP90α may represent a promising strategy to disrupt hypoxic adaptation through complementary mechanisms. Such a dual-targeting approach is anticipated to not only suppress tumor growth but also sensitize hypoxic cancer cells to conventional chemotherapy. These considerations prompted us to explore hypoxia-targeted dual hCA IX/HSP90α inhibitors as a potential therapeutic strategy for breast cancer.

Inspired by the privileged role of coumarins as selective hCA IX inhibitors and HSP90 modulators, the ATP-mimetic and anticancer properties of phenyl quinazolinones, and the therapeutic relevance of targeting hypoxia-associated vulnerabilities, a series of alkyl coumarin-tethered phenyl quinazolinones (4a–l) was rationally designed and synthesized as hypoxia-targeted dual hCA IX/HSP90α inhibitors. The synthesized compounds were evaluated for their inhibitory activities against hCA I, II, IX, and XII, followed by antiproliferative studies under normoxic and hypoxic conditions against MCF-7 and MDA-MB-231 breast cancer cells. The most promising compounds were further investigated for their HSP90α inhibitory activity, chemosensitizing potential toward doxorubicin (DOX), apoptosis-inducing effects, and binding behavior through molecular docking and molecular dynamics simulations, complemented by *in silico* ADME and toxicity studies.

## Results and discussion

### Chemistry

Our ongoing efforts focused on designing and synthesizing new heterocyclic compounds with anticipated pharmacological activity.^21,22^ In this study, we report a method for the preparation of twelve target alkyl coumarin-tethered phenyl quinazolinones (4a–l), which was illustrated in Scheme 1. 2-Mercaptoquinazolin-4(3*H*)-one derivatives (1a–d) were synthesized *via* nucleophilic attack of the amino group of anthranilic acid derivatives on the carbon atom of (N=C=S) moiety of phenyl isothiocyanate derivatives, affording the thiourea intermediates.^23^ This reaction was facilitated by triethylamine (TEA) as a basic catalyst, which deprotonated the primary amino group of anthranilic acid derivatives and converted it to triethylammonium.^24^ The thiourea intermediates then underwent an intramolecular nucleophilic attack of the anionic amino group towards the carbonyl group of the carboxylic acid.^25^ The proton from triethylammonium was transferred to the nitrogen atom and subsequently to the hydroxyl group, forming a good leaving group and yielding neutral intermediates. These intermediates then eliminate a water molecule to generate the thione intermediates,^26^ which could tautomerize to thiol intermediates (1a–d),^25,27^ which were confirmed with the appearance of the thiol proton singlet signal around 13.00 ppm in ^1^H NMR^28^ as shown in Schemes 1 and 2.

**Scheme 1:**
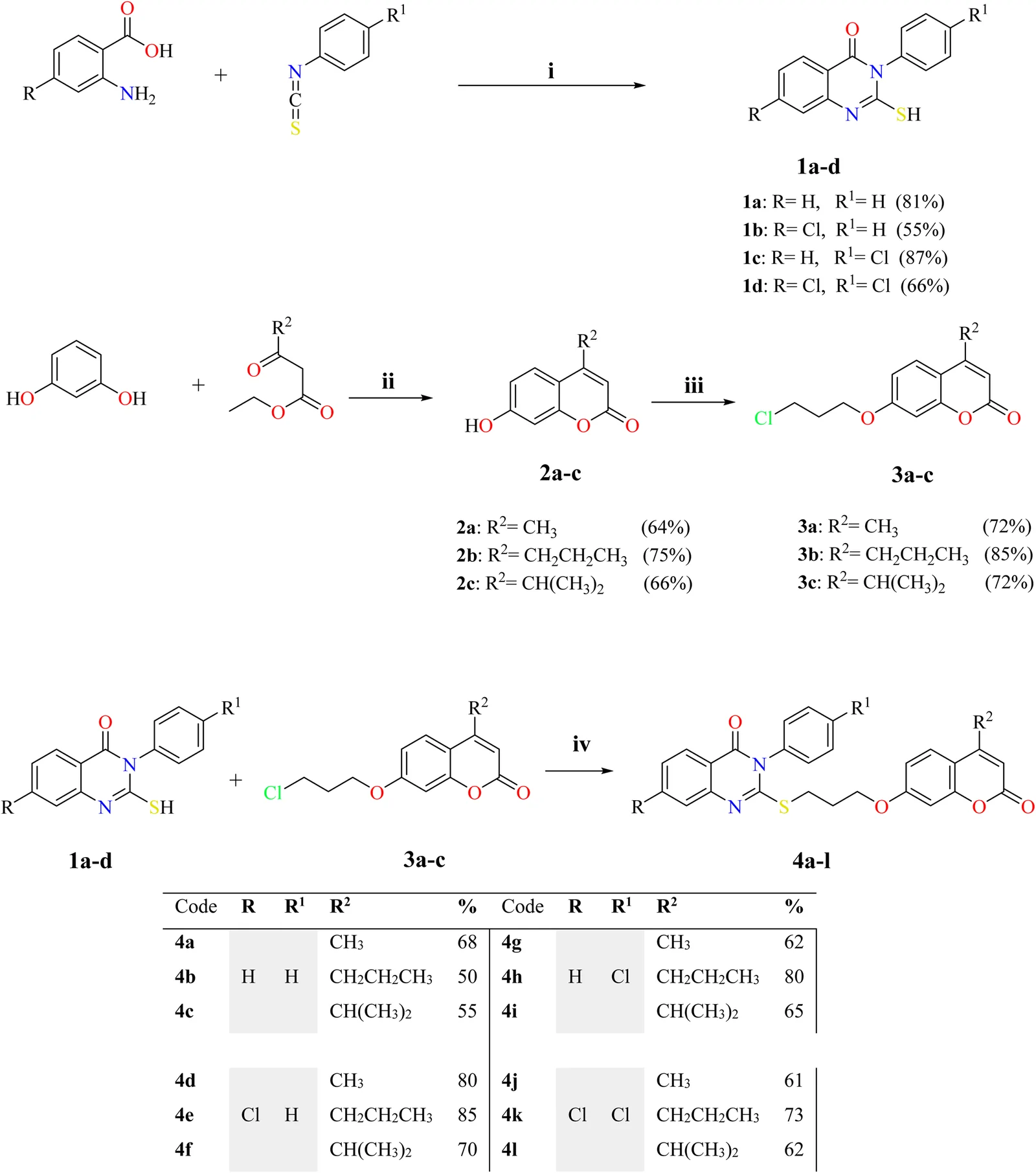
Synthetic steps for target alkyl coumarin-tethered phenyl quinazolinones (4a–l). Reagents and conditions: (i) TEA, abs. ethanol, 80 °C, 24 h; (ii) conc. H_2_SO_4_ (98.0%), 25 °C, 24 h; (iii) Cl(CH_2_)_3_Br, anhydrous K_2_CO_3_, dry acetone, 60 °C, 48 h; and (iv) anhydrous K_2_CO_3_, abs. ethanol, 80 °C, 48 h. Yields (50–85%).

**Scheme 2:**
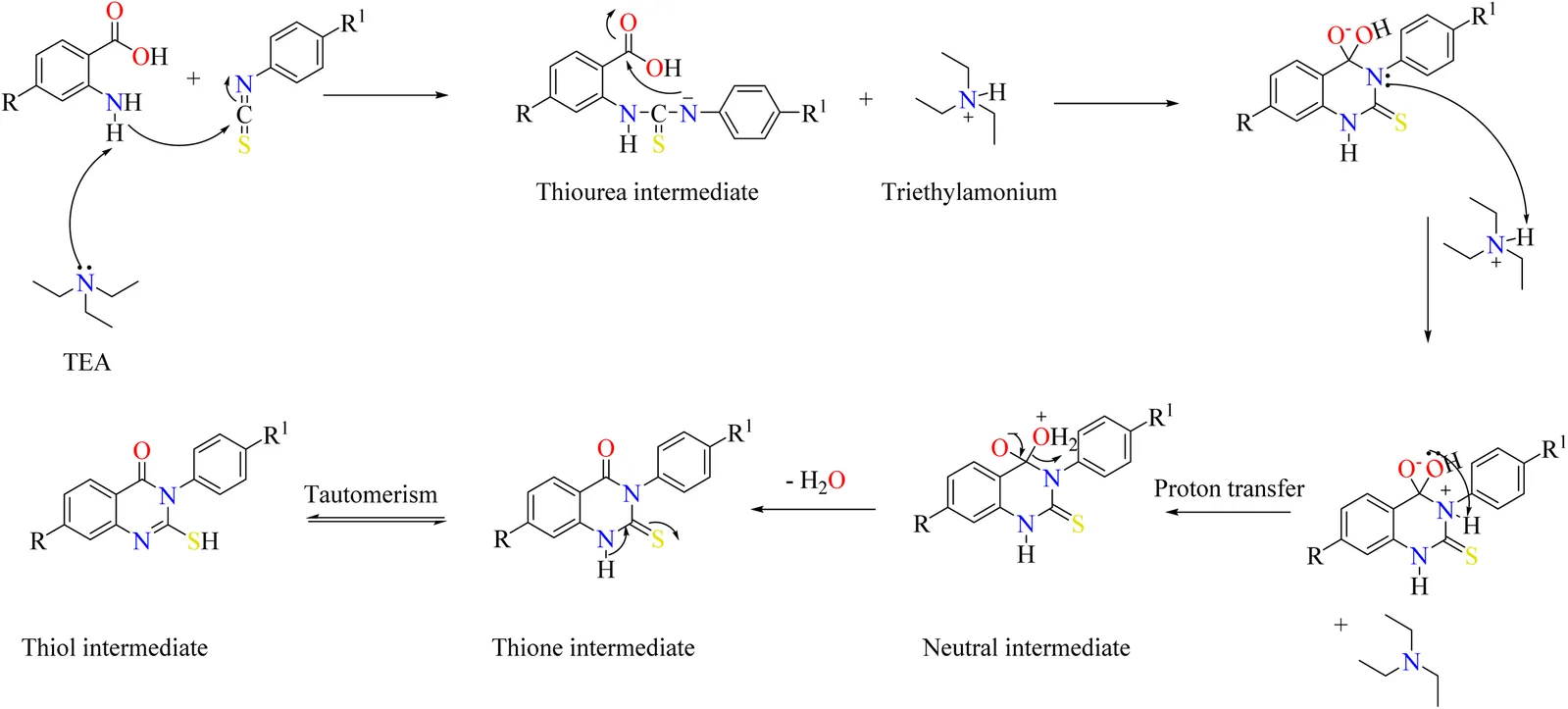
Proposed mechanism for the synthesis of thiol intermediates (1a–d).

7-Hydroxy-2*H*-chromen-2-one derivatives (2a–c) were synthesized by Pechmann condensation of resorcinol with β-Keto esters (such as ethyl acetoacetate, ethyl butyrylacetate, and ethyl isobutyrylacetate) in the presence of conc. H_2_SO_4_ (98.0%).^29,30^ This additive protonated the ketone (C=O) of β-keto esters, yielding oxonium intermediates, which were converted to the hydrogen sulfate ion. The resorcinol reacted with oxonium intermediates, affording intermediates (i) *via* electrophilic substitution. Intermediates (i) rearomatized to intermediates (ii). The hydrogen sulphate ion facilitated this rearomatization. Intermediates (ii) were then cyclized through intramolecular transesterification, yielding intermediates (iii). Finally, intermediates (iii) underwent acid-induced dehydration reaction to afford intermediates (2a–c),^31,32^ which were confirmed *via*^1^H NMR by the appearance of (3-H_coumarin_) proton singlet signal at 6.08 ppm, along with the disappearance of β-keto ester (CH_2_) protons singlet signal around 3.56 ppm^33^ as shown in Schemes 1 and 3.

**Scheme 3:**
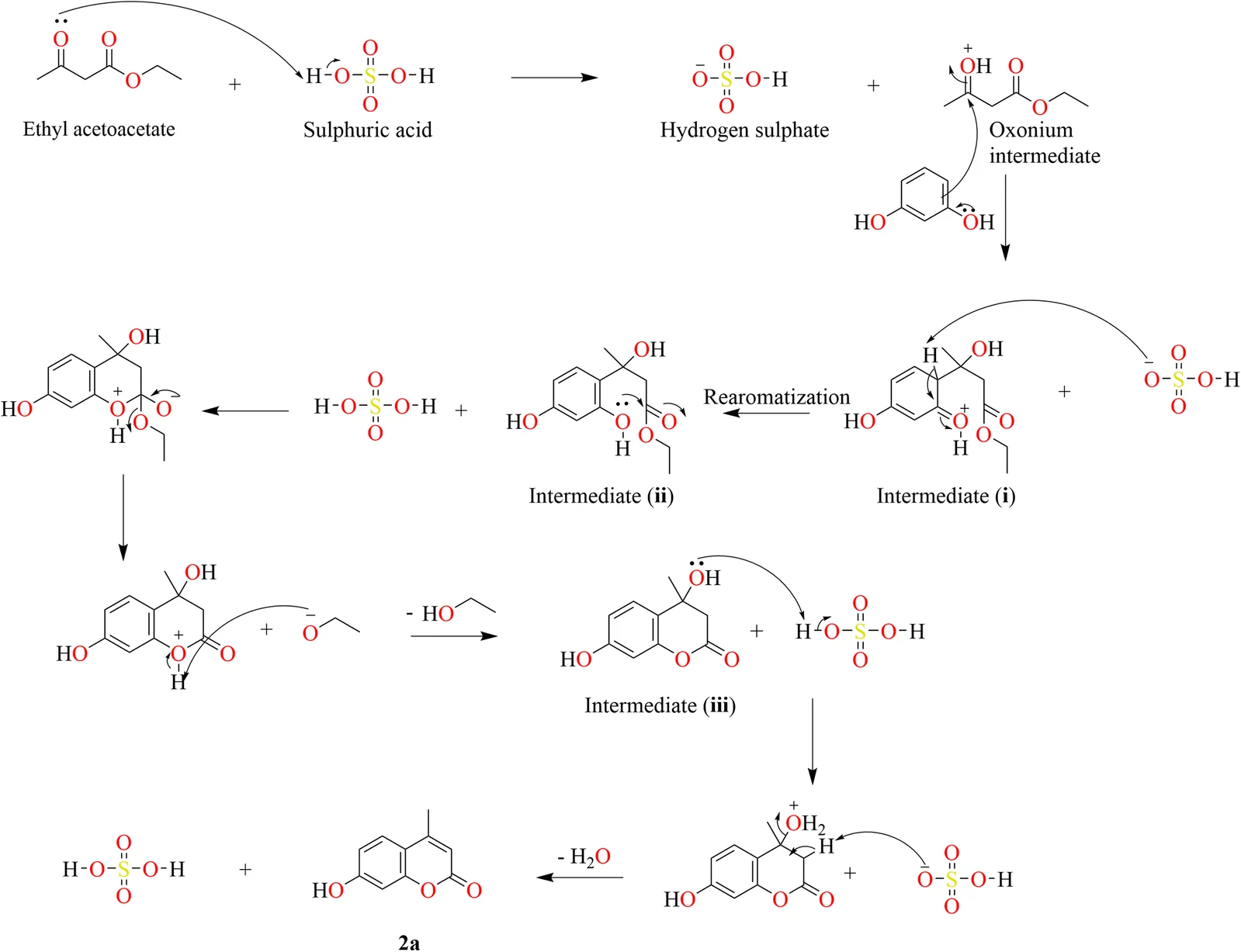
Proposed mechanism for synthesis of 7-hydroxy-4-methyl-2*H*-chromen-2-one (2a).

7-(3-Chloropropoxy)-2*H*-chromen-2-one derivatives (3a–c) were synthesized by nucleophilic substitution reaction of intermediates (2a–c) with 1-bromo-3-chloropropane in dry acetone using anhydrous potassium carbonate as a basic additive.^34^ This additive deprotonated the phenolic hydroxyl group (OH) of intermediates (2a–c) and generated phenoxide intermediates,^35^ which then underwent a nucleophilic attack on the carbon atom bearing bromine of 1-bromo-3-chloropropane. This resulted in *O*-alkylation, yielding intermediates (3a–c)^36^ as shown in Schemes 1 and S1.

Finally, the target compounds (4a–l) were synthesized by nucleophilic substitution of thiol intermediates (1a–d) with appropriate coumarin derivatives (3a–c) in absolute ethanol, using anhydrous potassium carbonate as a base. This basic additive deprotonated the thiol intermediates (1a–d), generating thiolate ions that then underwent nucleophilic attack at the carbon bearing a chlorine atom in the coumarin derivatives (3a–c). This resulted in *S*-alkylation and furnished the target compounds (4a–l) with a yield range of (50–85%), as shown in Scheme 1.

The elemental and spectral data (^1^H NMR, ^13^C NMR-DEPTQ, HMQC, HRMS (ESI), and HPLC) supported the structure of target compounds (4a–l). ^1^H NMR spectra displayed the singlet signal at (3.23–3.26 ppm) correlated to the two protons of SCH_2_ as well as the disappearance of the thiol proton singlet signal around 13.00 ppm.^28^^13^C NMR-DEPTQ spectra; **CH** and CH_3_ [positive (↑)], C_Q_ and CH_2_ [negative (↓)]^37^ while negative peaks of SCH_2_ appeared downside at (29.00–29.08 ppm).

To further support the structural assignment, compound 4b was subjected to two-dimensional HMQC analysis. The obtained spectrum established direct one-bond ^1^H–^13^C correlations, allowing unambiguous assignment of the key aliphatic and heteroatom-bearing carbon signals. The propyl substituent at C-4 of the coumarin scaffold was confirmed through the correlations of the terminal CH_3_ group (*δ*_H_ 0.93/*δ*_C_ 14.23), the central methylene group (*δ*_H_ 1.58/*δ*_C_ 21.91), and the methylene carbon directly attached to the coumarin nucleus (*δ*_H_ 2.69/*δ*_C_ 33.36). Likewise, the OCH_2_CH_2_CH_2_S tether was unequivocally assigned through the correlations observed for OCH_2_ (*δ*_H_ 4.13/*δ*_C_ 67.35), the central methylene group (*δ*_H_ 2.09/*δ*_C_ 28.35), and SCH_2_ (*δ*_H_ 3.23/*δ*_C_ 29.00). Furthermore, the characteristic coumarin H-3 resonance at *δ*_H_ 6.11 exhibited a direct correlation with C-3 at *δ*_C_ 110.74, confirming the integrity of the coumarin nucleus. Collectively, the HMQC data provided additional evidence for the successful construction of the designed alkyl coumarin-tethered phenyl quinazolinone framework and validated the assignments derived from the one-dimensional NMR experiments.

Within a reasonable range (±0.4), elemental analysis findings match the molecular formula for each compound. The high-resolution mass spectra of the synthesized compounds, such as chlorinated compounds, showed peaks corresponding to the molecular ions and their isotopes. The purity levels of all compounds were greater than 95%, as determined by HPLC analysis.

## Biological investigations

### Carbonic anhydrase isoforms inhibition assay

#### Potency parameter

Using a stopped-flow CO_2_ hydrase assay,^38–40^ the target alkyl coumarin-tethered phenyl quinazolinones (4a–l) were evaluated as inhibitors of the four physiologically and pharmacologically significant carbonic anhydrase isoforms: the cytosolic hCA I and II, and the transmembrane tumor-associated isoforms, hCA IX and XII. Tables 1–3 displayed enzyme inhibition constants (*K*_I_s) and calculated selectivity ratios, respectively. As positive controls, acetazolamide (AAZ) and colladonin were also used in the experiments.

Based on the inhibitory data listed in Table 1 as *K*_I_ values for the synthetic analogues (4a–l), the following structure–activity relationship (SAR) was illustrated:

**Table 1:** Inhibitory activity of target alkyl coumarin-tethered phenyl quinazolinones (4a–l) on hCA isoforms I, II, IX, and XII compared with AAZ and colladonin

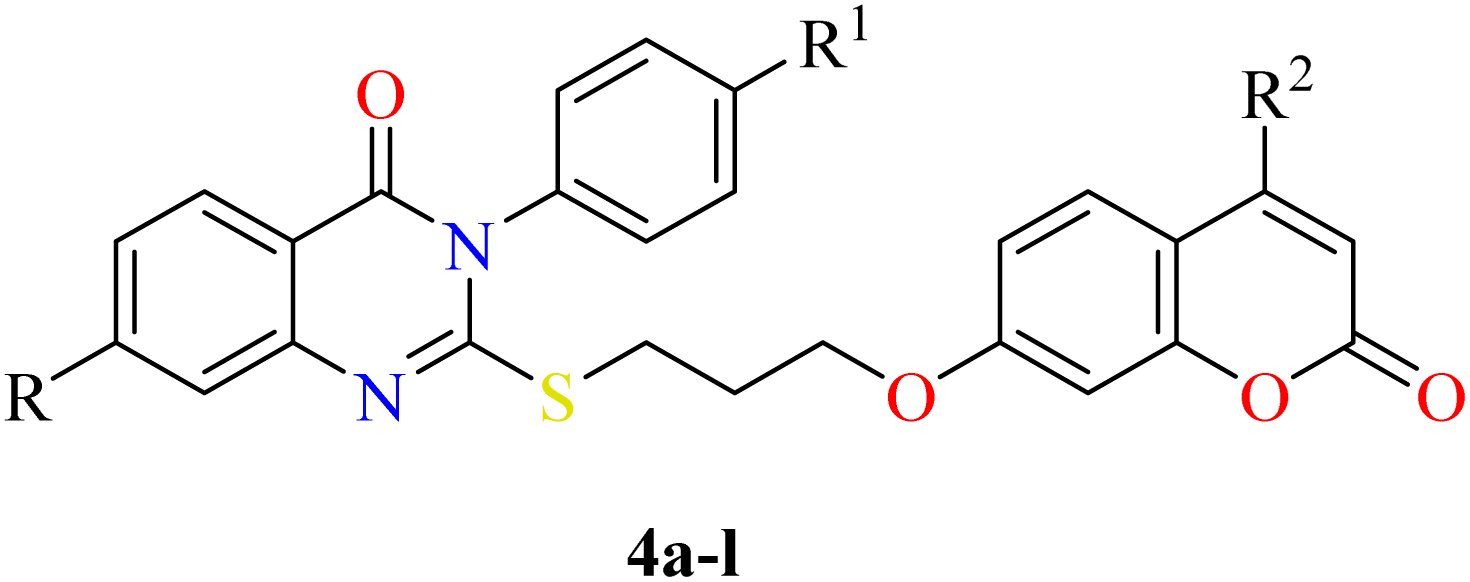							
Codes	*R*	*R* ^1^	*R* ^2^	*K*_I_ (nM)^a^			
				hCA I	hCA II	hCA IX	hCA XII
4a	H	H	CH_3_	79 800	>100 000	58.6	41.2
4b	H	H	CH_2_CH_2_CH_3_	>100 000	>100 000	51.7	36.8
4c	H	H	CH(CH_3_)_2_	83 200	>100 000	66.9	50.1
4d	Cl	H	CH_3_	70 500	83 900	55.3	47.7
4e	Cl	H	CH_2_CH_2_CH_3_	>100 000	>100 000	81.2	70.4
4f	Cl	H	CH(CH_3_)_2_	>100 000	76 100	101.3	54.9
4g	H	Cl	CH_3_	87 400	>100 000	60.5	66.8
4h	H	Cl	CH_2_CH_2_CH_3_	>100 000	>100 000	72.0	131.6
4i	H	Cl	CH(CH_3_)_2_	84 600	92 400	148.7	94.2
4j	Cl	Cl	CH_3_	>100 000	80 600	120.3	54.1
4k	Cl	Cl	CH_2_CH_2_CH_3_	>100 000	87 700	85.6	60.8
4l	Cl	Cl	CH(CH_3_)_2_	>100 000	>100 000	90.2	78.0
Acetazolamide (AAZ)				250.0	12.0	25.0	5.7
Colladonin				29 000	10 000	90.0	210.0

a Values are reported as the mean of three independent experiments using the stopped-flow technique; experimental errors were within ±5–10% of the reported values.

(i) The inhibitory activity of the investigated compounds (4a–l) against the cytosolic hCA I was performed in comparison to AAZ (*K*_I_ = 250.0 nM). Based on the inhibition constants (*K*_I_ values), these compounds exhibited significantly weaker activity than AAZ, with values ranging from 70 500 nM to over 100 000 nM. Among them, compound (4d, *K*_I_ = 70 500 nM) appears to be the most effective, albeit still far less potent than AAZ.

(ii) Evaluation of the cytosolic hCA II inhibition findings showed that (4a–l) exhibited weak inhibitory activity, ranging from *K*_I_ = 76 100 to >100 000 nM. This group's best analogue includes (4f), which has a *K*_I_ value of 76 100 nM.

(iii) The assayed compounds (4a–l) strongly inhibited the hCA IX isoform compared with the cytosolic isoforms, with *K*_I_ values of 51.7–148.7 nM, and they were less potent than the positive control (AAZ, *K*_I_ = 25.0 nM). All compounds exhibited two-digit nanomolar inhibition activity toward the isoform IX (*K*_I_s: 51.7–90.2 nM) except compounds (4f), (4i), and (4j) that showed three-digit nanomolar inhibitory action, *K*_I_s = 101.3, 148.7, and 120.3 nM, respectively. The top four compounds are (4a), (4b), (4d), and (4g) with *K*_I_ values 58.6, 51.7, 55.3, and 60.5 nM, respectively.

Starting from compound (4a) with *K*_I_ = 58.6 nM, the addition of a chlorine atom to the quinazolinone ring (4d) enhanced the inhibitory action towards isoform IX with a *K*_I_ value of 55.3 nM. However, shifting the chlorine atom to the phenyl ring (4g) led to a decrease in potency (*K*_I_ = 60.5 nM). The addition of an extra chlorine atom to the quinazolinone ring (4j) caused a substantial loss of inhibitory activity (*K*_I_ = 120.3 nM), making it nearly half as effective as (4g), as shown in Fig. 2.

**Fig. 2:**
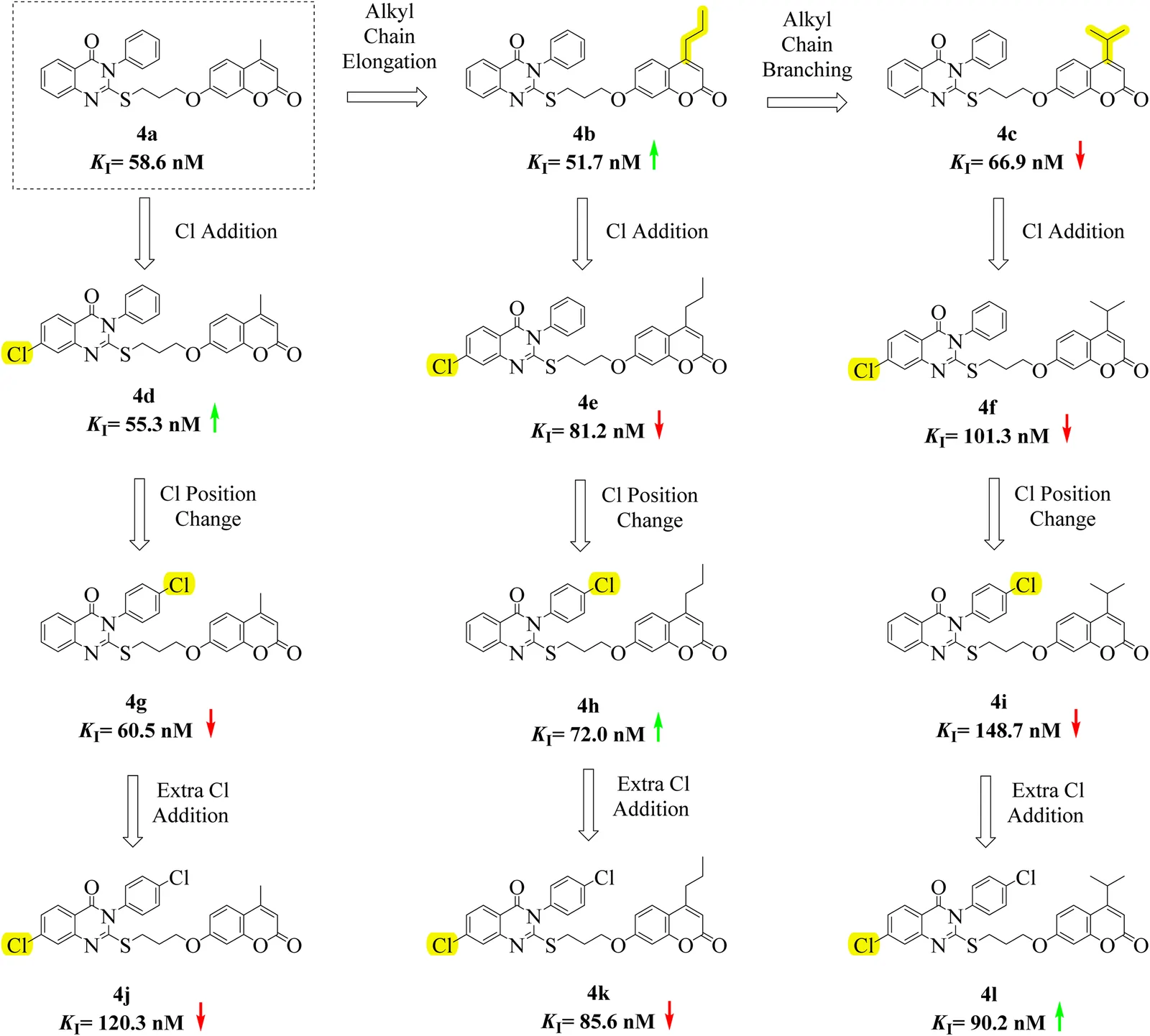
Structure–activity relationship (SAR) analysis of alkyl coumarin-tethered phenyl quinazolinones (4a–l) as carbonic anhydrase IX inhibitors. Structural modifications are highlighted in yellow, whereas green upward arrows and red downward arrows indicate enhanced and reduced inhibitory activities, respectively.

Compound (4a, *K*_I_ = 58.6 nM) showed an increase in inhibitory activity upon switching methyl to a *n*-propyl group, as shown in compound (4b, *K*_I_ = 51.7 nM). Adding a chlorine atom to the quinazolinone ring in compound (4b) resulted in diminished activity (4e, *K*_I_ = 81.2 nM). Changing the chlorine atom's position from the quinazolinone ring to the phenyl ring restored some activity (4h, *K*_I_ = 72.0 nM). Adding an extra chlorine atom further decreased inhibitory activity (4k, *K*_I_ = 85.6 nM) as shown in Fig. 2.

The switching of *n*-propyl to an isopropyl group in compound (4b, *K*_I_ = 51.7 nM) decreased the inhibitory activity of compound (4c, *K*_I_ = 66.9 nM). Similarly, adding a chlorine atom to the quinazolinone ring further decreased activity (4f, *K*_I_ = 101.3 nM), and repositioning that chlorine atom to the phenyl ring caused an even more pronounced drop (4i, *K*_I_ = 148.7 nM). However, introducing an additional chlorine atom reversed that trend and improved inhibition by about 1.5-fold (4l, *K*_I_ = 90.2 nM) as shown in Fig. 2.

(iv) The target compounds (4a–l) greatly inhibited the isoform hCA XII (like hCA IX) compared to cytosolic isoforms, with *K*_I_s ranging from 36.8 to 131.6 nM, although they are less active than AAZ (*K*_I_ = 5.7 nM). This group's best analogue includes (4b), which has a *K*_I_ value of 36.8 nM.

The influence of linker length on activity could not be evaluated in the present series because the linker was maintained unchanged across compounds 4a–l; systematic variation of linker length will be considered in future optimization studies.

For a more detailed quantitative comparison of the observed SAR trends, the key matched structural modifications and their corresponding effects on the inhibitory potency of hCA IX are summarized in Table 2.

**Table 2:** Quantitative summary of the key structure–activity relationships (SARs) of compounds 4a–l against hCA IX

Comparison	Structural modification	hCA IX *K*_I_ (nM)	SAR effect
4a → 4b	CH_3_ → *n*-propyl	58.6 → 51.7	Improved
4b → 4c	*n*-Propyl → *i*-propyl	51.7 → 66.9	Reduced
4a → 4d	Cl introduction at *R*	58.6 → 55.3	Slightly improved
4b → 4e	Cl introduction at *R*	51.7 → 81.2	Reduced
4c → 4f	Cl introduction at *R*	66.9 → 101.3	Reduced
4a → 4g	Cl introduction at *R*^1^	58.6 → 60.5	Slightly reduced
4b → 4h	Cl introduction at *R*^1^	51.7 → 72.0	Reduced
4c → 4i	Cl introduction at *R*^1^	66.9 → 148.7	Markedly reduced
4g → 4j	Additional Cl at *R*	60.5 → 120.3	∼2-Fold reduced
4h → 4k	Additional Cl at *R*	72.0 → 85.6	Reduced
4i → 4l	Additional Cl at *R*	148.7 → 90.2	∼1.65-Fold improved

#### Selectivity parameter

The core sequences of human CAs are at least 30% identical, and the active regions of all hCA isoforms exhibit significant conservation. Planning isoform-selective CAIs for hCA IX with minimal off-target activity is challenging due to the high amino acid conservation among the hCA isoforms.

Table 3 shows that the target compounds (4a–l) exhibited remarkable selectivity for the tumor-associated hCA IX and hCA XII isoforms over the cytosolic hCA I and hCA II isoforms. The selectivity ratios (SRs) were calculated by dividing the *K*_I_ value for the off-target cytosolic isoform (hCA I or hCA II) by that of the corresponding tumor-associated isoform (hCA IX or hCA XII). Accordingly, higher SR values indicate greater preferential inhibition of the tumor-associated isoform and, consequently, greater sparing of the cytosolic isoforms. Thus, the SR values provide a quantitative measure of isoform selectivity rather than inhibitory potency itself.

**Table 3:** Selectivity ratios (SRs) for hCA IX & XII inhibition over hCA I & II for targeted compounds (4a–l)

Compounds	Selectivity ratio (SR)^a^			
	I/IX	II/IX	I/XII	II/XII
4a	1361.7	>1706.4	1936.8	>2427.1
4b	>1934.2	>1934.2	>2717.3	>2717.3
4c	1243.6	>1494.7	1660.6	>1996.0
4d	1274.8	1517.1	1477.9	1758.9
4e	>1231.5	>1231.5	>1420.4	> 1420.4
4f	>987.1	751.2	>1821.4	1386.1
4g	1444.6	>1652.8	1308.3	>1497.0
4h	>1388.8	>1388.8	>759.8	>759.8
4i	568.9	621.3	898.0	980.8
4j	>831.2	669.9	>1848.4	1489.8
4k	>1168.2	1024.5	>1644.7	1442.4
4l	>1108.6	>1108.6	>1282.0	>1282.0
Acetazolamide (AAZ)	10.0	0.5	43.8	2.1
Colladonin	32.2	111.1	13.8	47.6

a Selectivity ratios were calculated as *K*_i_(hCA I or hCA II)/*K*_i_(hCA IX or hCA XII). Higher ratio values indicate greater selectivity toward the tumor-associated hCA IX or hCA XII isoform over the corresponding cytosolic hCA I or hCA II isoform.

(i) The SR_I/IX_ of the target compounds (4a–l) ranged from 568.9 to >1934.2. All compounds showed higher selectivity when compared with AAZ (SR_I/IX_ = 10.0) and colladonin (SR_I/IX_ = 32.2). Compound (4a) exhibited a strikingly high selectivity for hCA IX, with SR_I/IX_ = 1361.7 (almost 136 times that of AAZ). Switching the methyl group to *n*-propyl in (4b) led to an even greater selectivity (SR_I/IX_ = >1934.2). Interestingly, adding a chlorine atom to the phenyl ring in (4g) enhanced selectivity slightly (SR_I/IX_: 1361.7 → 1444.6), but when modifying the methyl group to isopropyl while also introducing a chlorine atom to the phenyl ring in (4i), the selectivity dropped significantly (SR_I/IX_: 1361.7 → 568.9). Similarly, adding chlorine atoms to both the quinazolinone and phenyl rings in (4j) also reduced selectivity (SR_I/IX_: 1361.7 → 831.2).

(ii) The target compounds (4a–l) showed high selectivity for hCA IX over II, with SR_II/IX_ ranging from 621.3 to >1934.2 relative to AAZ; SR_II/IX_ = 0.5 for colladonin. The analogues (4a) and (4b) exhibited the highest selectivity against hCA IX, with SR_II/IX_ values of >1706.4 and >1934.2, respectively (both are about 3000 times those of AAZ). Addition of a chlorine atom to the phenyl ring in (4g) does not significantly alter selectivity (SR_II/IX_: >1706.4 → >1652.8). Switching the methyl group to isopropyl and adding a chlorine atom to the phenyl ring in compound (4i) led to a decrease in selectivity (SR_II/IX_: >1706.4 → 621.3) by about three-fold. Similarly, adding chlorine atoms to both the quinazolinone and phenyl rings in (4j) also resulted in reduced selectivity (SR_II/IX_: >1706.4 → 669.9).

(iii) The computed SR_I/XII_ for compounds (4a–l) ranged from >759.8 to >2717.3. The analogue (4b) demonstrated the greatest hCA XII selectivity, with an SR_I/XII_ > 2717.3 (62-fold that of AAZ). Analogue (4a) showed the second selective compound with SR_I/XII_ = 1936.8. Adding chlorine atoms to quinazolinone and phenyl rings in (4j) does not significantly alter selectivity (SR_I/XII_: 1936.8 → >1848.4). Switching the methyl group to *n*-propyl and adding a chlorine atom to the phenyl ring in compound (4h) led to a decline in selectivity (SR_I/XII_: 1936.8 → >759.8) by about 2.5-fold. Also, a drop in selectivity (SR_I/XII_: 1936.8 → 898.0) was observed upon switching the methyl group to isopropyl and adding a chlorine atom to the phenyl ring in compound (4i).

(iv) All analogues (4a–l) showed greater selectivity for SR_II/XII,_ ranging from >759.8 to >2717.3 relative to AAZ (SR_II/XII_ = 2.1). The analogue (4b) demonstrated the greatest hCA XII selectivity, with SR_II/XII_ = >2717.3 (1300 times that of AAZ), followed by compound (4a) with SR_II/XII_ = >2427.1 (1155 times that of AAZ). Switching the methyl group to *n*-propyl and adding a chlorine atom to the phenyl ring in compound (4h) led to a decrease in selectivity (SR_II/XII_: >2427.1 → >759.8) by about three-fold. Also, a decrease in selectivity (SR_II/XII_: >2427.1 → 980.8) was observed upon replacing the methyl group with an isopropyl group and adding a chlorine atom to the phenyl ring in compound (4i).

Based on their potent dual hCA IX/XII inhibitory activity and remarkable selectivity over the off-target hCA I and hCA II isoforms, compounds 4a, 4b, 4d, and 4g were selected as representative hits for further biological investigations. Overall, the present alkyl coumarin-tethered phenyl quinazolinones emerged as potent and highly selective non-sulfonamide inhibitors of the tumor-associated hCA IX and hCA XII isoforms. Notably, tethering the coumarin moiety through the benzene ring, as exemplified by the present C-7-substituted series, appears to preserve the inherent isoform selectivity of coumarins while conferring remarkable nanomolar potency, highlighting this substitution pattern as a promising design approach for developing selective tumor-associated carbonic anhydrase inhibitors.

#### *In vitro* antiproliferative activity

Remarkably, hCA IX and hCA XII are markedly upregulated in breast cancer under hypoxic conditions and are implicated in extracellular acidification, tumor progression, and therapeutic resistance. Therefore, two breast cancer cell lines with distinct molecular characteristics, namely MCF-7 (luminal A, ER-positive, less aggressive) and MDA-MB-231 (triple-negative, highly invasive, and hypoxia-adapted), were selected as representative models to evaluate the anticancer potential of the synthesized compounds. Based on their potent dual hCA IX/XII inhibitory activity and significant selectivity over the off-target hCA I and hCA II isoforms, compounds 4a, 4b, 4d, and 4g were chosen for further biological investigations. The *in vitro* antiproliferative activities were assessed under both normoxic and hypoxic conditions using the MTT assay, with DOX employed as a reference drug. As summarized in Table 4, all investigated compounds displayed enhanced antiproliferative activity under hypoxic conditions compared with normoxia, whereas DOX exhibited the opposite trend. This finding is particularly noteworthy, as hCA IX and hCA XII are preferentially expressed under hypoxia, suggesting that the improved efficacy of the investigated compounds may arise, at least in part, from their selective targeting of these hypoxia-associated isoforms.

**Table 4:** *In vitro* anticancer screening (in µM) against breast cancer cell lines (MCF-7 and MDA-MB-231) under normal and hypoxic conditions (all treated for 48 h).^a^

Codes	IC_50_ ± S.D (µM) against breast cancer cell lines			
	MDA-MB-231		MCF-7	
	Under normoxia	Under hypoxia	Under normoxia	Under hypoxia
4a	9.78 ± 0.43	6.49 ± 0.36	16.12 ± 0.51	10.05 ± 0.76
4b	5.15 ± 0.49	4.06 ± 0.15	7.18 ± 0.35	4.99 ± 0.19
4d	48.27 ± 5.19	19.46 ± 0.61	30.52 ± 1.24	21.90 ± 1.88
4g	55.70 ± 3.64	42.10 ± 3.06	34.39 ± 1.56	29.30 ± 2.00
DOX	1.65 ± 0.05	8.90 ± 0.54	2.38 ± 0.07	6.58 ± 0.16

a IC_50_ (µM) values are mean ± S.D. of five experiments.

Among the tested compounds, derivatives 4a and 4b emerged as the most active antiproliferative agents. Structurally, both compounds possess an unsubstituted quinazolinone nucleus and an unsubstituted *N*3-phenyl ring, differing only in the alkyl substituent at C-4 of the coumarin moiety, where 4a bears a methyl group, and 4b incorporates an *n*-propyl substituent. Interestingly, elongation of the alkyl substituent from methyl (4a) to *n*-propyl (4b) consistently enhanced antiproliferative activity across both breast cancer cell models under both normoxic and hypoxic conditions, establishing 4b as the lead member of the series. Compound 4b exhibited IC_50_ values of 5.15 and 4.06 µM against MDA-MB-231 cells and 7.18 and 4.99 µM against MCF-7 cells under normoxic and hypoxic conditions, respectively. Thus, the superior cellular profile of 4b over 4a was consistently observed across the two investigated breast cancer models rather than being restricted to a single cell line or oxygenation condition. Notably, 4b was somewhat more potent against MDA-MB-231 than MCF-7 cells, particularly under hypoxia. Although MCF-7 and MDA-MB-231 represent biologically distinct breast cancer models, evaluating only two cell lines does not permit a general conclusion about subtype-independent efficacy. Broader evaluation across additional breast cancer molecular subtypes will therefore be required to establish the generalizability of 4b's antiproliferative activity.

In contrast, the chlorinated analogues 4d and 4g displayed moderate antiproliferative activity. Compound 4d bears a chlorine atom at C-7 of the quinazolinone scaffold, whereas 4g contains a chlorine substituent at the *para*-position of the *N*3-phenyl ring, while both compounds possess a methyl group at C-4 of the coumarin moiety. Although these derivatives retained the characteristic enhancement of activity under hypoxic conditions, their antiproliferative effects were generally inferior to those of 4a and 4b. Interestingly, both compounds exhibited slightly greater activity against MDA-MB-231 cells than MCF-7 cells. Compound 4b reported higher activity than DOX under hypoxia against both breast cancer cell lines. Accordingly, the position of the chlorine substituent on either the quinazolinone or the *N*3-phenyl moiety appears to influence the cellular antiproliferative activity; however, broader evaluation across additional breast cancer models would be required to establish any subtype-dependent SAR.

Furthermore, the cytotoxicity profiles of the selected potent compounds (4a, 4b, 4d, and 4g) were evaluated against the non-tumorigenic human mammary epithelial cell line MCF-10A. Encouragingly, all four compounds exhibited minimal to negligible cytotoxicity toward MCF-10A cells, with IC_50_ values exceeding 50 µM. These findings indicate a favorable *in vitro* cytotoxicity profile and preferential activity toward breast cancer cells relative to non-tumorigenic mammary epithelial cells. Collectively, these results support the promising anticancer potential of this series while indicating limited cytotoxicity toward MCF-10A cells at the tested concentrations.

#### Adjuvant activity evaluation

Under normoxic conditions, compound 4b exhibited moderate antiproliferative activity against MDA-MB-231 and MCF-7 breast cancer cells, with IC_50_ values of 5.15 ± 0.49 and 7.18 ± 0.35 µM, respectively. DOX was more potent under the same conditions, displaying IC_50_ values of 1.65 ± 0.05 and 2.38 ± 0.07 µM, respectively. The DOX + 4b combination was also evaluated under normoxia and exhibited IC_50_ values of 3.76 ± 0.15 µM against MDA-MB-231 cells and 4.49 ± 0.28 µM against MCF-7 cells. Thus, under normoxic conditions, the combination did not improve the antiproliferative activity of DOX alone.

In contrast, hypoxia markedly reduced the sensitivity of both breast cancer cell lines to DOX. The IC_50_ of DOX increased from 1.65 ± 0.05 to 8.90 ± 0.54 µM in MDA-MB-231 cells and from 2.38 ± 0.07 to 6.58 ± 0.16 µM in MCF-7 cells. Notably, compound 4b maintained appreciable antiproliferative activity under hypoxia, with IC_50_ values of 4.06 ± 0.15 and 4.99 ± 0.19 µM against MDA-MB-231 and MCF-7 cells, respectively.

Importantly, under hypoxic conditions, combination treatment with DOX + 4b substantially enhanced the antiproliferative activity relative to DOX alone, reducing the corresponding IC_50_ values from 8.90 ± 0.54 to 2.09 ± 0.09 µM in MDA-MB-231 cells and from 6.58 ± 0.16 to 3.94 ± 0.22 µM in MCF-7 cells (Table 5). This corresponds to approximately 4.3- and 1.7-fold improvements in DOX potency, respectively. In contrast, such an improvement was not observed under normoxic conditions, where DOX alone remained more potent than the combination.

**Table 5:** Antiproliferative effects of DOX, compound 4b, and the combination of DOX with a non-cytotoxic concentration of 4b against MCF-7 and MDA-MB-231 breast cancer cells under normoxic and hypoxic conditions^a^

Condition	Cell line	IC_50_ ± S.D. (µM)		
		Single therapy		Combination therapy
		4b	DOX	DOX + 4b
Normoxia	MDA-MB-231	5.15 ± 0.49	1.65 ± 0.05	3.76 ± 0.15
	MCF-7	7.18 ± 0.35	2.38 ± 0.07	4.49 ± 0.28
Hypoxia	MDA-MB-231	4.06 ± 0.15	8.90 ± 0.54	2.09 ± 0.09
	MCF-7	4.99 ± 0.19	6.58 ± 0.16	3.94 ± 0.22

a IC_50_ (µM) values are mean ± S.D. of five experiments.

Collectively, the direct comparison between normoxic and hypoxic conditions indicates that the beneficial effect of combining 4b with DOX was preferentially manifested under hypoxia, where a marked reduction in DOX sensitivity was observed. These findings suggest that 4b can sensitize hypoxic breast cancer cells to DOX and partially counteract hypoxia-associated chemoresistance. Nevertheless, the precise molecular mechanisms underlying this hypoxia-associated chemosensitizing effect remain to be established through further mechanistic investigations.

#### *In vitro* HSP90α (N-terminal) inhibition assay

Based on their potent dual hCA IX/XII inhibitory activities, remarkable selectivity profiles, and promising antiproliferative effects under hypoxic conditions, compounds 4a, 4b, 4d, and 4g were further evaluated for their inhibitory activities against the N-terminal ATP-binding domain of HSP90α. The results obtained are summarized in Table 6 as IC_50_ values derived from five independent experiments.

**Table 6:** IC_50_ values of the most potent compounds (4a, 4b, 4d, and 4g) against HSP90α

Compounds	IC_50_ (nM)^a^
	HSP90α
4a	19.91 ± 1.00
4b	13.21 ± 1.54
4d	25.62 ± 1.19
4g	43.24 ± 2.01
Ganetespib	12.15 ± 0.70

a IC_50_ values are presented as the mean ± SD of five independent experiments.

As shown in Table 6, all tested compounds exhibited potent HSP90α inhibitory activities in the nanomolar range, with IC_50_ values ranging from 13.21 ± 1.54 to 43.24 ± 2.01 nM. Among them, compound 4b emerged as the most active derivative, displaying an IC_50_ value of 13.21 ± 1.54 nM, which is comparable to that of the reference inhibitor ganetespib (IC_50_ = 12.15 ± 0.70 nM). Compound 4a, which differs from 4b only by possessing a methyl group instead of an *n*-propyl substituent at the C-4 position of the coumarin moiety, exhibited lower potency (IC_50_ = 19.91 ± 1.00 nM). This observation indicates that alkyl chain elongation at C-4 of the coumarin scaffold enhances HSP90α inhibitory activity.

In contrast, the introduction of a chlorine substituent negatively affected the inhibitory activity. Compound 4d, bearing a chlorine atom at the C-7 position of the quinazolinone nucleus, exhibited an IC_50_ value of 25.62 ± 1.19 nM, whereas compound 4g, possessing a *para*-chloro substituent on the *N*3-phenyl ring, showed the weakest activity among the tested derivatives (IC_50_ = 43.24 ± 2.01 nM). Collectively, these findings suggest that the HSP90α inhibitory activity of alkyl coumarin-tethered phenyl quinazolinones is favored by alkyl chain elongation at the coumarin moiety, whereas chlorine substitution on either the quinazolinone or *N*3-phenyl rings exerts an unfavorable effect on activity. Notably, the superior activity of compound 4b against both hCA IX/XII and HSP90α highlights its potential as a promising dual-target anticancer agent.

#### Effects of compound 4b on the apoptotic markers

To further elucidate the mechanism underlying the antiproliferative activity of the lead compound 4b, its effect on the transcriptional levels of key apoptosis-related genes was investigated in MDA-MB-231 breast cancer cells by qRT-PCR following 72 h of treatment at its IC_50_ concentration under hypoxic conditions. As summarized in Table 7, treatment with 4b resulted in a significant upregulation of the pro-apoptotic markers p53, caspase-9, and BAX by 3.12-, 5.16-, and 3.12-fold, respectively, relative to the untreated control cells. In contrast, the anti-apoptotic gene Bcl-2 was markedly downregulated to 0.51-fold of the control level.

**Table 7:** Effect of compound 4b on levels of BAX, Bcl-2, p53, and caspase-9 genes expression in MDA-MB-231 cells treated for 72 h

Sample	Gene expression (fold change)^a^				
	p53	Caspase-9	BAX	Bcl-2	BAX/Bcl-2 ratio
MDA-MB-231	1.00 ± 0.05	1.00 ± 0.11	1.00 ± 0.08	1.00 ± 0.06	1.00 ± 0.07
4b/MDA-MB-231	3.12 ± 0.31**	5.16 ± 0.61**	3.12 ± 0.36*	0.51 ± 0.04**	6.12 ± 0.85**

a Values are given as changes from the corresponding control (MDA-MB-231) group, which is set to ‘1’. *p < 0.05, and **p < 0.01 indicate statistically significant differences from the corresponding control in unpaired *t*-tests.

Notably, the BAX/Bcl-2 ratio increased dramatically from 1.00 in the untreated cells to 6.12 following treatment with 4b. Since the BAX/Bcl-2 balance is a key determinant of mitochondrial outer membrane permeabilization and intrinsic apoptotic signaling, this pronounced shift toward a pro-apoptotic state strongly suggests activation of the mitochondrial apoptotic pathway. Furthermore, the substantial elevation of caspase-9 expression, an initiator caspase in the intrinsic apoptotic cascade, provides additional evidence supporting this mechanism. Collectively, these findings demonstrate that compound 4b induces apoptosis in MDA-MB-231 cells predominantly through activation of the p53-mediated intrinsic (mitochondrial) apoptotic pathway, thereby contributing to its potent antiproliferative activity under hypoxic conditions.

To provide a concise overview of the biological evaluation and lead-selection process, the experimental screening cascade is summarized in Fig. 3. The schematic illustrates the sequential selection of the most promising candidates from compounds 4a–l and the subsequent identification and biological characterization of compound 4b as the lead candidate.

**Fig. 3:**
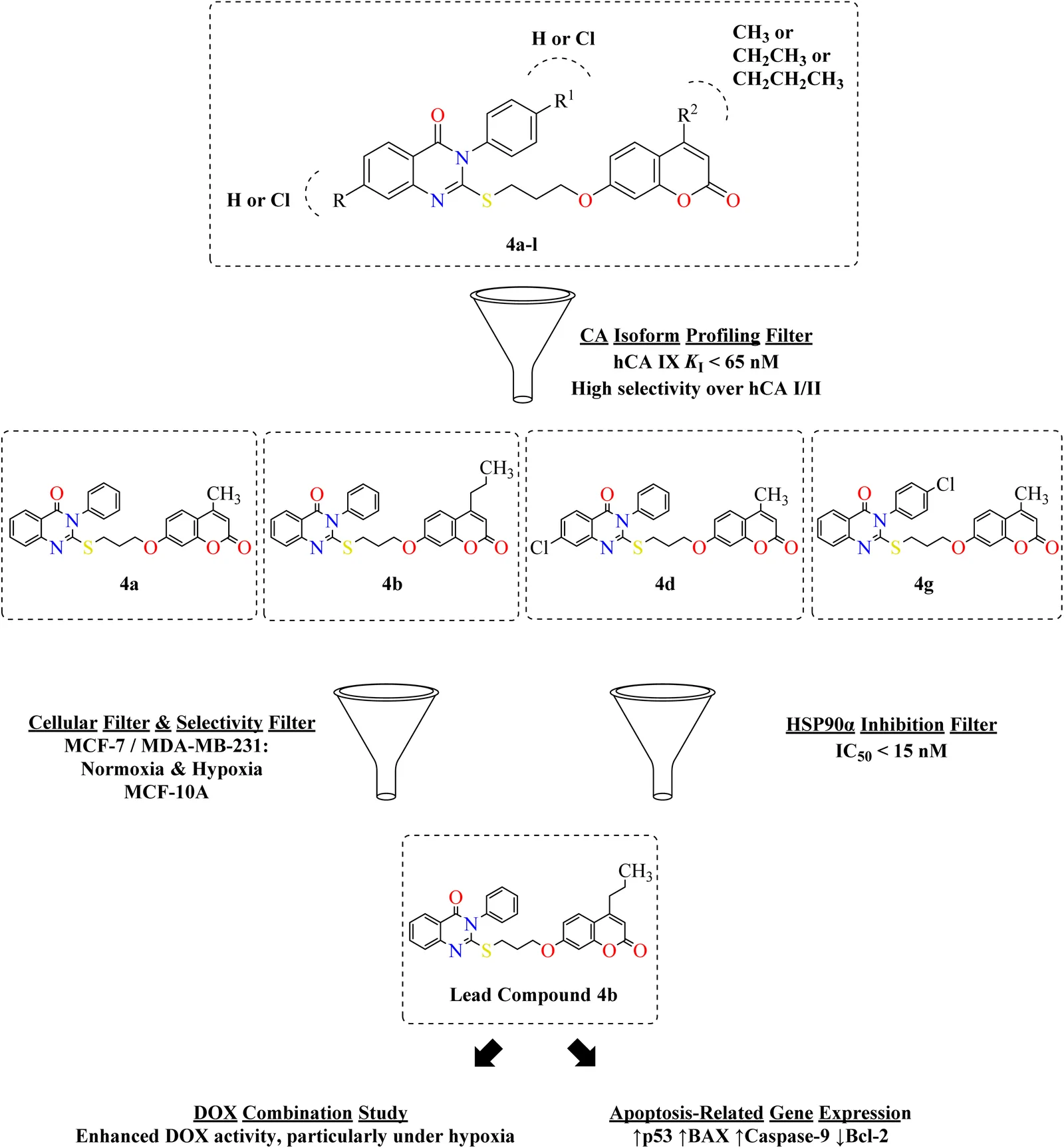
Schematic overview of the biological screening cascade and lead identification of compound 4b.

### *In silico* studies

#### Predicted ADME studies

Strong biochemical activity should be accompanied by favorable pharmacokinetic properties for a molecule to be considered a promising therapeutic candidate. Therefore, the predicted ADME profile of the lead compound 4b was evaluated using the ADMETlab 3.0 web server and compared with those of ganetespib, colladonin, and novobiocin, which were selected as pharmacologically relevant reference compounds previously used in the biological evaluation of the present study. This comparison was intended to contextualize the predicted physicochemical and pharmacokinetic characteristics of 4b, rather than to compare the inhibitory potencies of these compounds. The radar plots in Fig. 4 summarize the predicted physicochemical and ADME descriptors, including molecular weight (MW), lipophilicity (log *P* and log *D*), solubility (log *S*), hydrogen-bond donors and acceptors (*n*HD and *n*HA), topological polar surface area (TPSA), and ring-related descriptors. As shown in Fig. 4, compound 4b exhibited a well-balanced ADME profile that largely resides within the recommended property space and is comparable to that of ganetespib and the reference coumarin derivatives. Although slight deviations were observed for lipophilicity-related parameters, particularly log *P*, these values remained within an acceptable range for orally active anticancer agents.

**Fig. 4:**
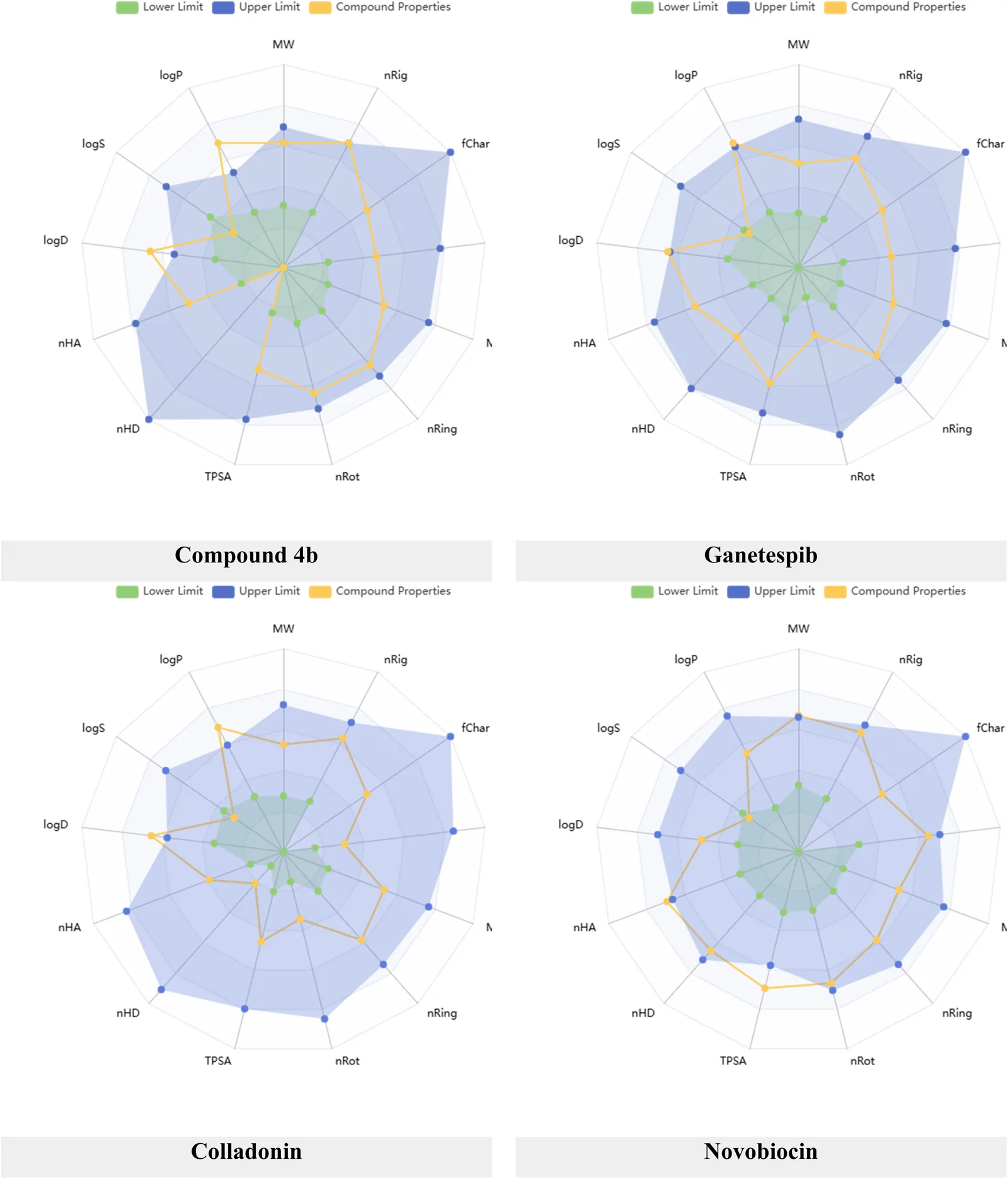
Radar plots of predicted ADME properties of compound 4b compared with ganetespib, colladonin, and novobiocin using ADMETlab 3.0. The orange lines represent the compounds' parameter values, while the blue regions indicate the upper acceptable limits and the green regions denote the lower acceptable limits.

Importantly, the predicted ADME profile of 4b was generally comparable to, and in some descriptors more favorable than, those of colladonin and novobiocin, suggesting that the incorporation of the phenyl quinazolinone moiety and alkyl linker did not markedly compromise the predicted druglike characteristics of the coumarin scaffold. Collectively, these findings indicate that compound 4b possesses a favorable balance between biological potency and predicted pharmacokinetic properties, supporting its further development as a hypoxia-targeted dual hCA IX/HSP90α inhibitor. Nevertheless, it should be emphasized that the present ADME assessment is based exclusively on *in silico* predictions and has not yet been experimentally validated. Accordingly, these predictions should be interpreted as preliminary estimates of the pharmacokinetic behavior of 4b, and dedicated experimental ADME and pharmacokinetic studies will be required to confirm its absorption, distribution, metabolism, and elimination profile.

#### *In silico* toxicity prediction

To gain preliminary insights into its safety profile, the lead compound 4b was subjected to an *in silico* toxicity assessment using the ProTox-II platform. The toxicity radar shown in Fig. 5 indicates that compound 4b exhibits an overall balanced safety profile with relatively low to moderate probabilities across most evaluated toxicity endpoints.

**Fig. 5:**
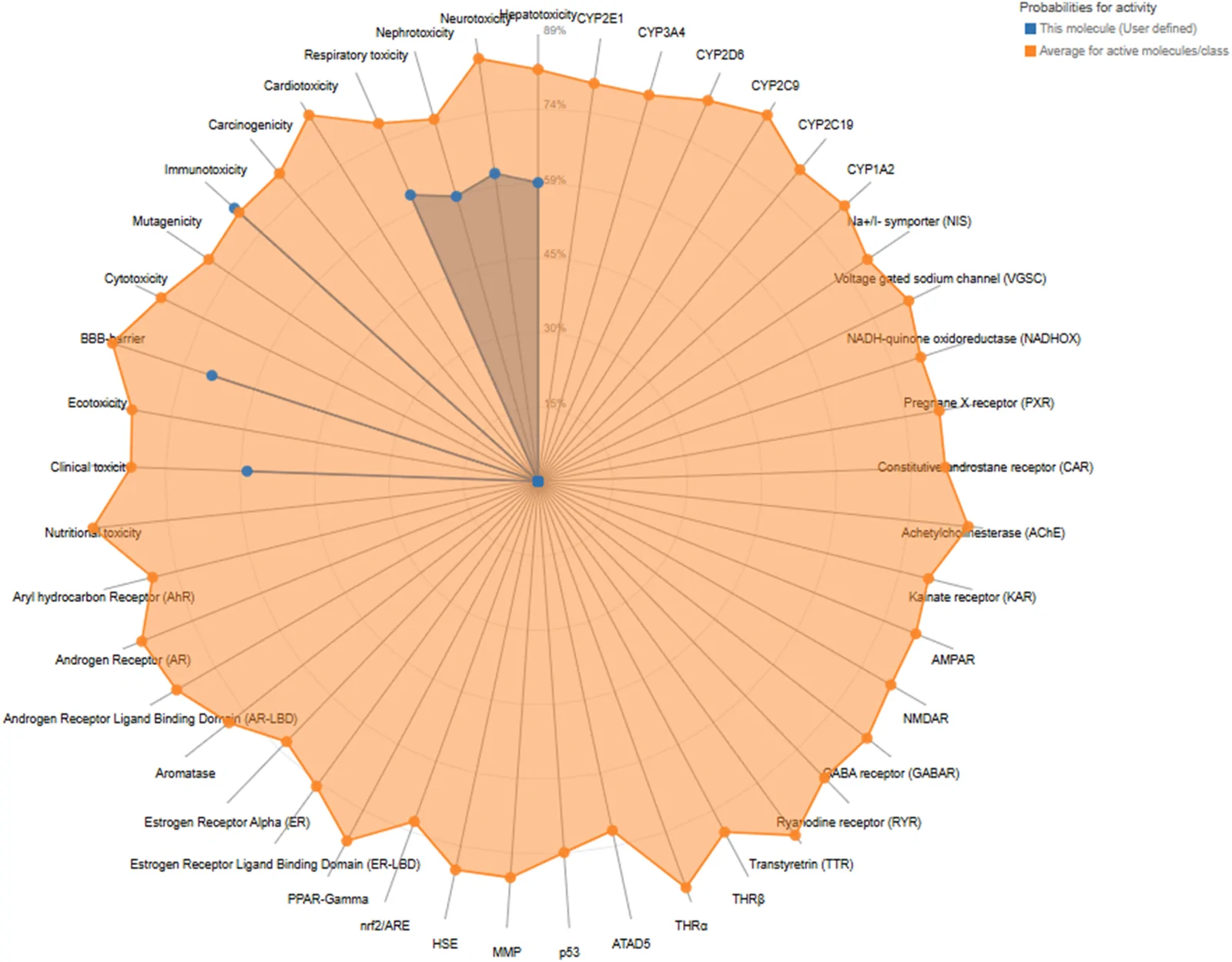
The toxicity radar chart for compound 4b.

In particular, 4b displayed no discernible neurotoxicity signal and only moderate probabilities of hepatotoxicity, nephrotoxicity, and respiratory toxicity. Moreover, no obvious toxicity liabilities were predicted across the investigated endpoints, although moderate probabilities were observed for a limited number of parameters. Overall, the predicted toxicity profile of 4b suggests a favorable safety profile and supports its continued investigation as a promising hypoxia-targeted dual hCA IX/HSP90α inhibitor. Nevertheless, additional experimental studies are required to validate these computational predictions.

#### Molecular docking

The X-ray structure of the human CA IX complex (PDB ID: 5FL6) was used for the molecular docking of the most promising compound 4b.^41^ The co-crystallized reference ligand was redocked into the CA IX active site to confirm that the docking protocol could reproduce the experimentally observed pose. The redocked pose closely overlapped with the experimentally observed pose, yielding a root-mean-square deviation (RMSD) of 0.51 Å, supporting the reliability of the applied docking protocol (Fig. 6).

**Fig. 6:**
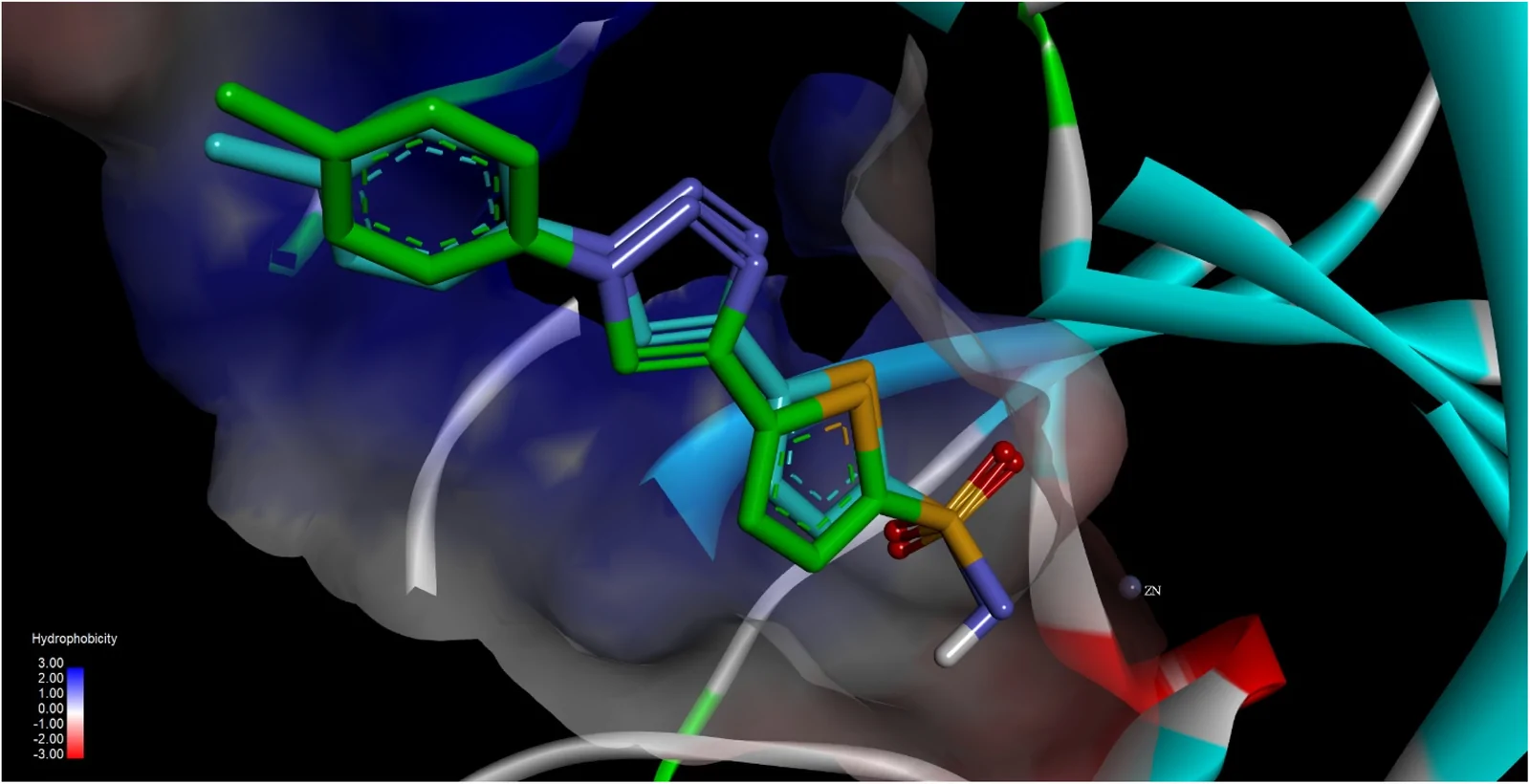
Overlay of the co-crystallized reference ligand (Y0R) (green color) with the redocked one (cyan color) in the CA IX active site (surface colors).

Carbonic anhydrases hydrolyze the coumarin ring *via* their esterase activity, yielding the corresponding 2-hydroxycinnamic acid as the active inhibitory compound.^42,43^ Crystallographic analysis of carbonic anhydrase in complex with substituted coumarin showed the hydrolysis product to be the *Z*-2-hydroxycinnamic acid derivative.^42,43^ On this basis, the *Z*-2-hydroxycinnamic acid form of compound 4b was selected for the docking study. The hydrolyzed *Z* product of compound 4b was docked into the CA IX active site, yielding a predicted binding pose with a docking score of −8.35 kcal mol^−1^. The carboxylate group, generated upon lactone hydrolysis, was oriented toward the catalytic Zn^2+^ ion, where its negatively charged oxygen atom engaged in attractive electrostatic interactions with Zn^2+^ ion, His68, and His94. The carboxylate group further participated in a conventional hydrogen bond with His68, while the central phenolic hydroxyl group was anchored by two conventional hydrogen bonds with Thr200 and Thr201. The distal quinazolinone ring formed a conventional hydrogen bond with Trp9, with the dentated phenyl ring participating in a π–π stacking interaction with His68. Finally, π–alkyl interactions were observed with multiple residues, including His94, His119, Leu199, Pro202, Pro203, and Trp210 (Fig. 7).

**Fig. 7:**
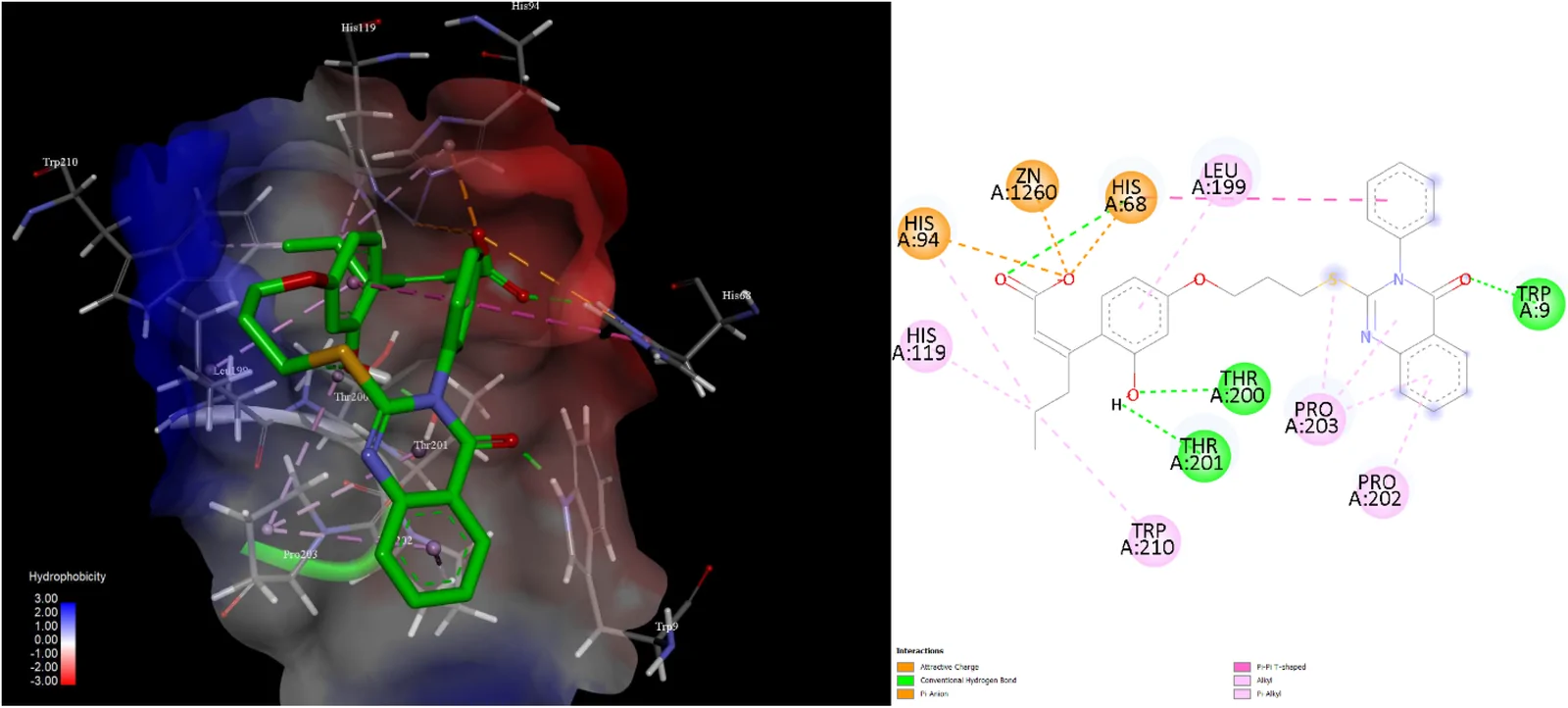
3D (left) and 2D (right) interaction plots of the hydrolyzed *Z* product of compound 4b in the CA IX active site.

For the HSP90α docking, the X-ray structure of the HSP90α-onalespib complex (PDB ID: 2XJX) was used.^44^ The co-crystallized onalespib was redocked into the N-terminal ATP-binding pocket to confirm that the docking protocol could reproduce the experimentally observed pose. The redocked pose was well aligned with the co-crystallized one, giving an RMSD of 1.89 Å, thereby supporting the reliability of the applied docking protocol (Fig. 8).

**Fig. 8:**
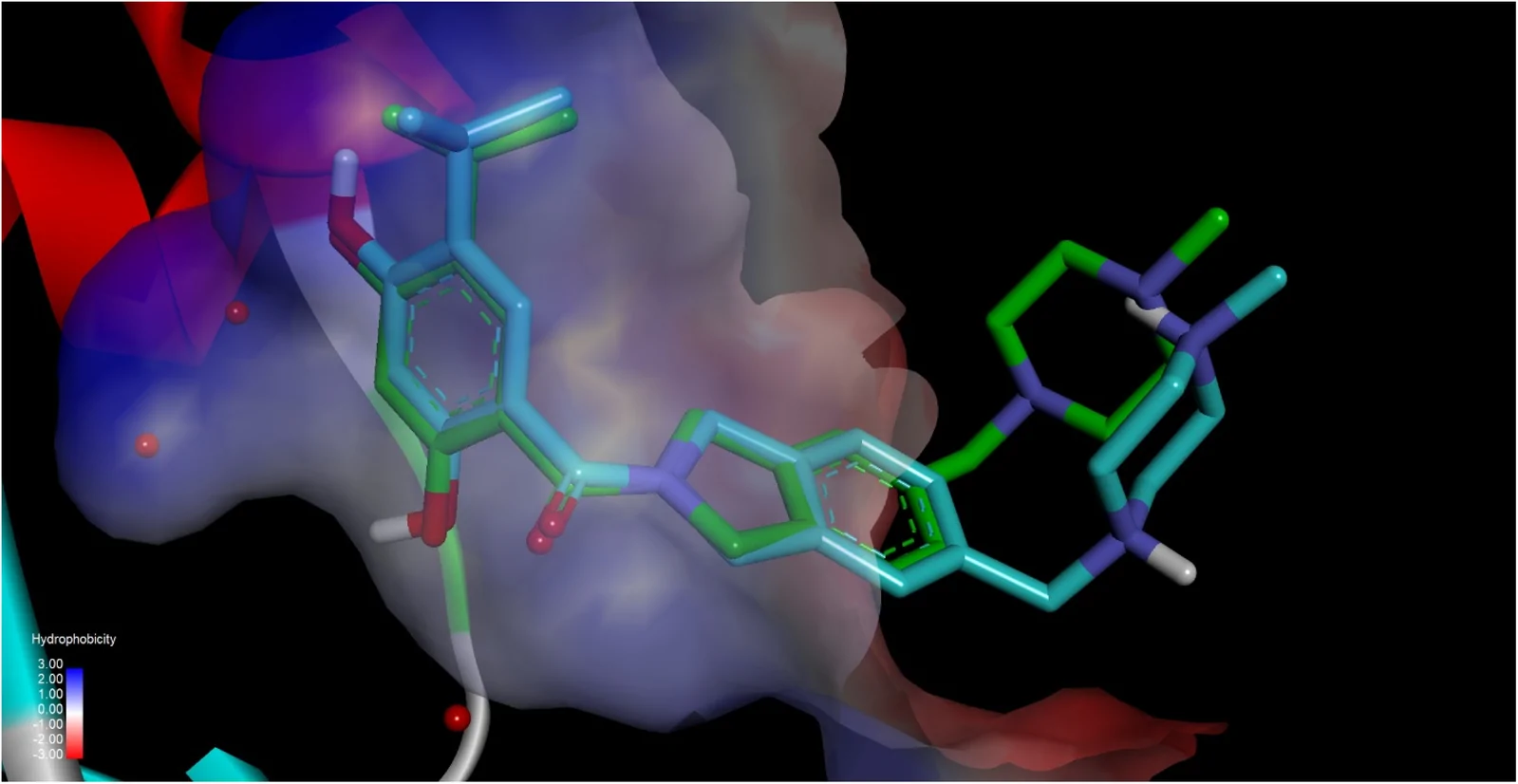
Overlay of the co-crystalized onalespib (green color) with the redocked one (cyan color) in the HSP90α N-terminal ATP-binding pocket (surface colors).

Compound 4b was predicted to adopt a favorable binding pose within the N-terminal ATP-binding pocket of HSP90α, with a docking score of −7.24 kcal mol^−1^. The coumarin carbonyl group engaged in a water-mediated hydrogen bond with Asp93 *via* HOH2084 and also participated in a carbon–hydrogen bond with Thr184. The ether oxygen of the linker formed a conventional hydrogen bond with Asn106. In addition, compound 4b established a π–sulfur interaction with Met98, an amide–π stacking interaction with Asn51, and a π–anion interaction with Asp54. Finally, several π–alkyl interactions were observed with Ala55, Lys58, Phe138, Val150, and Val186 (Fig. 9).

**Fig. 9:**
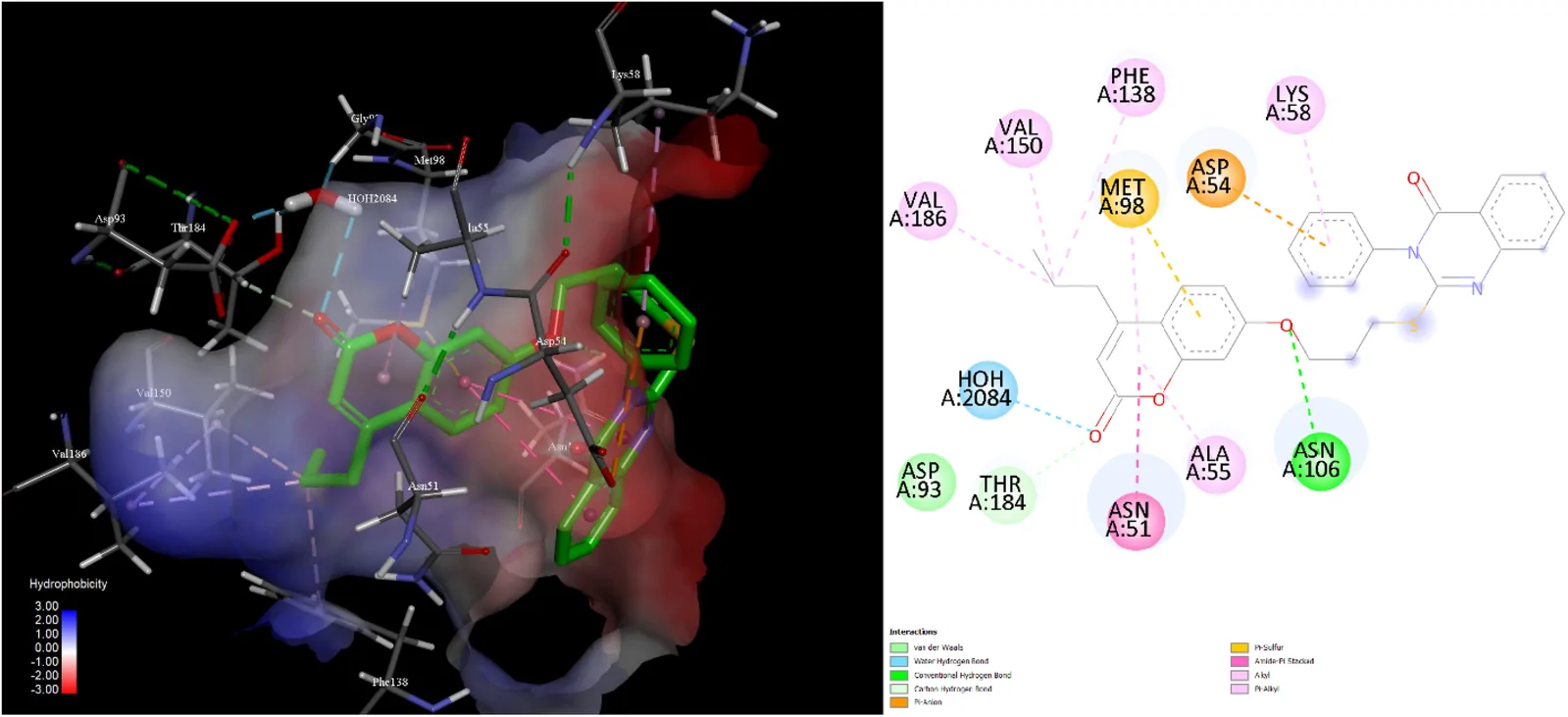
3D (left) and 2D (right) interaction plots of compound 4b in the HSP90α N-terminal ATP-binding pocket.

### Molecular dynamics simulation

To assess the dynamic stability of the docked complexes of compound 4b with CA IX and HSP90α,100 ns molecular dynamics simulations were performed and compared to those of the corresponding apo-CA IX and apo-HSP90α, respectively. Throughout the simulations, all four trajectories displayed stable temperature, pressure, and potential energy profiles (Fig. 10), indicating that all systems maintained stable simulation conditions.

**Fig. 10:**
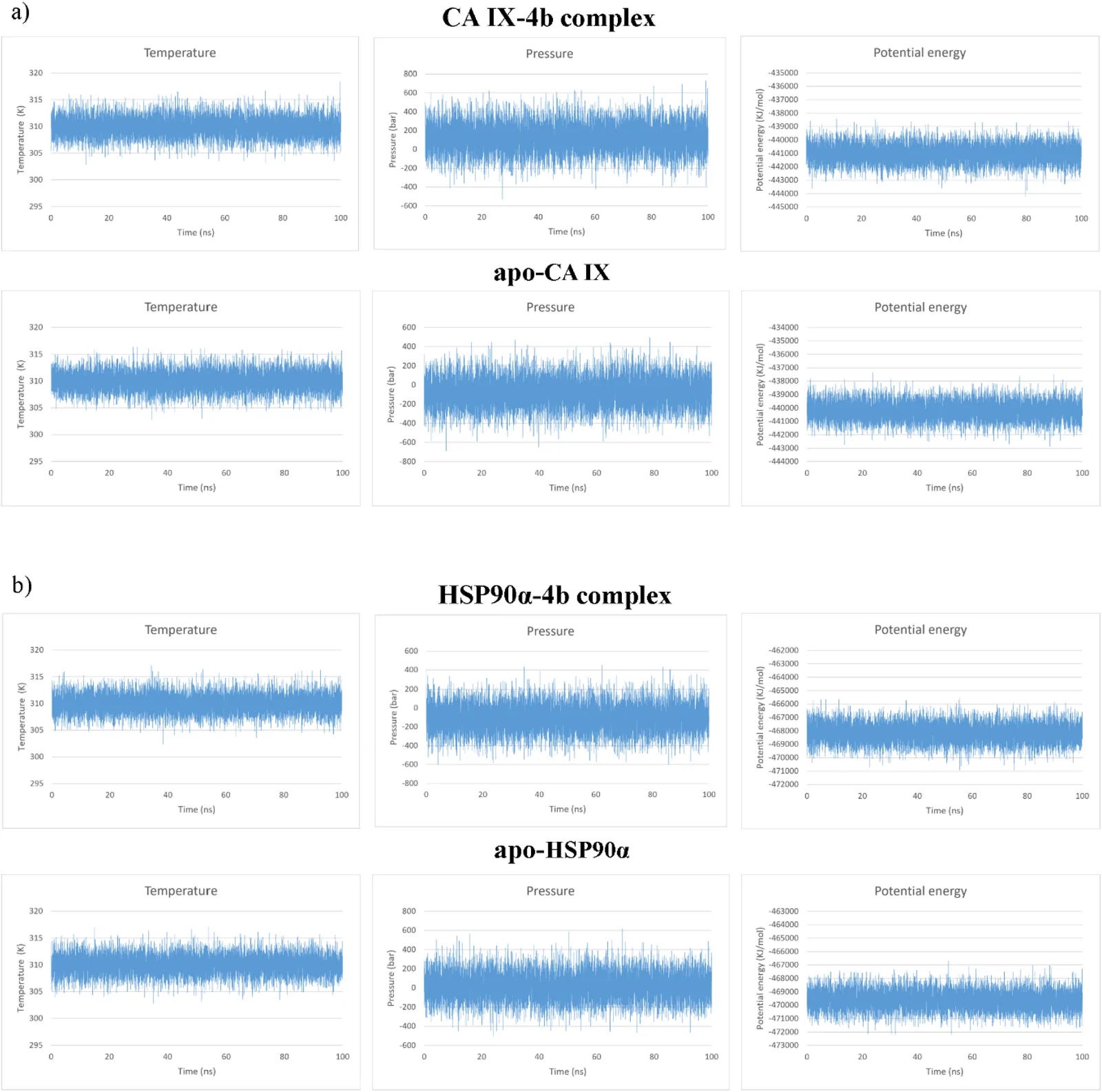
Temperature, pressure, and potential energy profiles for the CA IX-4b complex and apo-CA IX systems (a), and the HSP90α-4b complex and apo-HSP90α systems (b), over the 100 ns molecular dynamics simulations.

Regarding the RMSD analysis, the backbone RMSD of the CA IX-4b complex remained stable within approximately 0.9–1.7 Å, comparable to the backbone RMSD of the apo-CA IX (0.8–1.5 Å), while the RMSD of the overall complex stabilized within approximately 1.6–2.3 Å. The RMSD of compound 4b fluctuated within approximately 2.6–4.0 Å, due to the conformational flexibility of the linker. These findings support the stability of the CA IX-4b complex throughout the 100 ns simulation (Fig. 11a). For HSP90α, the backbone RMSD of the HSP90α-4b complex stabilized after an initial equilibration period and subsequently fluctuated mostly within the 3.0–4.0 Å range, while the apo-HSP90α backbone fluctuated within a lower range of approximately 2.5–3.5 Å. The higher RMSD observed in the backbone of the HSP90α-4b complex reflects conformational changes upon compound 4b binding rather than structural destabilization. The RMSD of the overall complex stabilized after the initial equilibration period and was mostly maintained within approximately 3.5–4.5 Å. The RMSD of compound 4b similarly stabilized after the initial equilibration period and fluctuated mostly within 2.5–4.0 Å, indicating the retention of compound 4b within the N-terminal ATP-binding pocket throughout the 100 ns simulation (Fig. 11b).

**Fig. 11:**
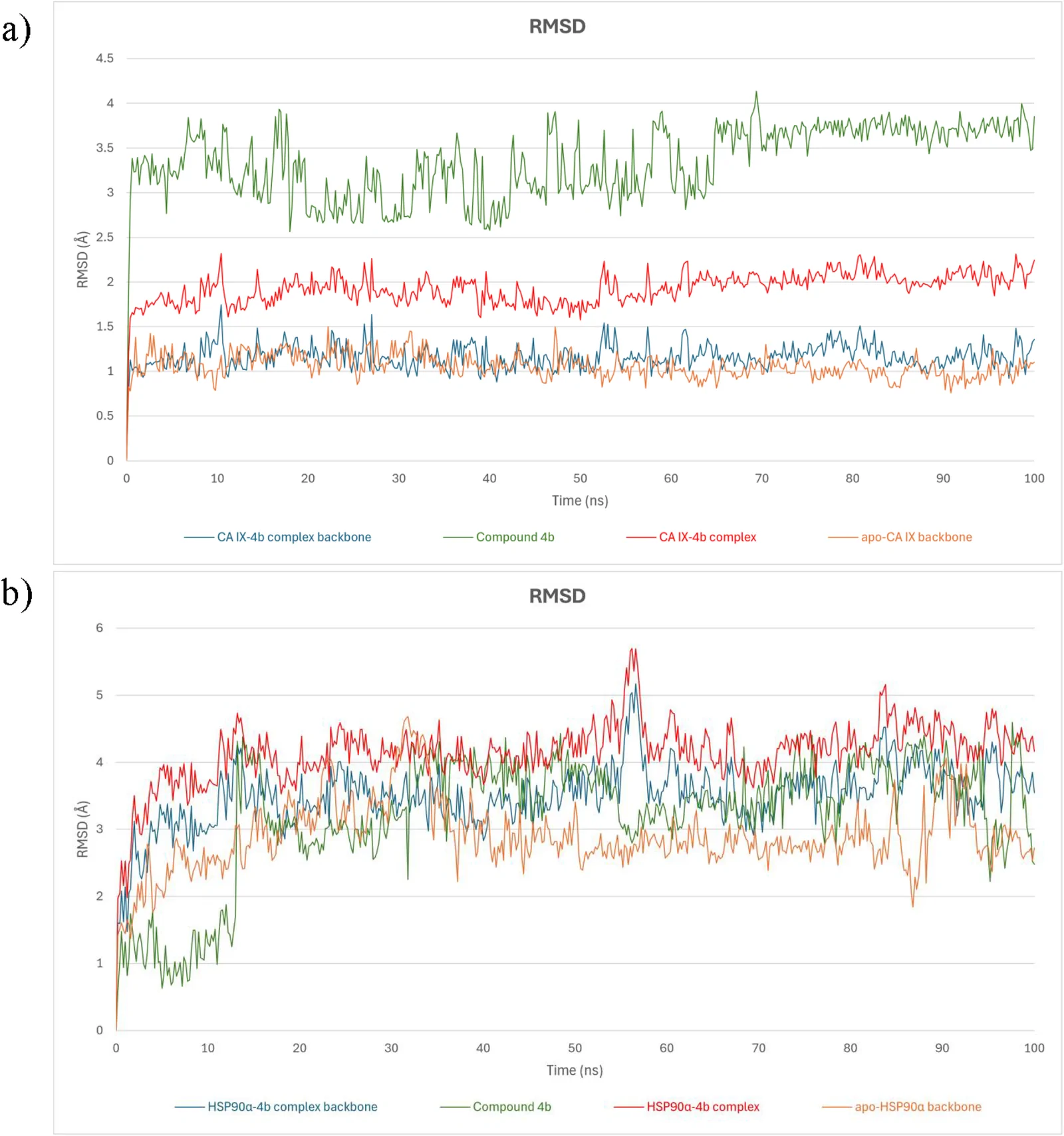
Root-mean-square deviation (RMSD) profiles over the 100 ns molecular dynamics simulation for the CA IX-4b complex backbone, compound 4b, CA IX-4b complex, and apo-CA IX backbone (a), and the HSP90α-4b complex backbone, compound 4b, HSP90α-4b complex, and apo-HSP90α backbone (b).

The root-mean-square fluctuation (RMSF) profiles for the CA IX-4b complex and apo-CA IX were comparable. The active-site residues involved in the docking pose, together with the catalytic Zn^2+^-coordinating residues His94, His96, and His119, exhibited low fluctuations below 1.5 Å, indicating that the binding of compound 4b didn't destabilize the CA IX active site (Fig. 12a). For HSP90α, the HSP90α-4b complex and apo-HSP90α profiles were largely overlapping. The key N-terminal ATP-binding pocket residues, Asp93 and Thr184, remained stable below 1 Å. In addition, the loop with residues 71–74, located at the rim of the ATP-binding cavity between the first and second α-helices of the HSP90α N-terminal domain, was stabilized in the HSP90α-4b complex relative to the apo-HSP90α, indicating that compound 4b induces ordering of this dynamic region upon binding (Fig. 12b).

**Fig. 12:**
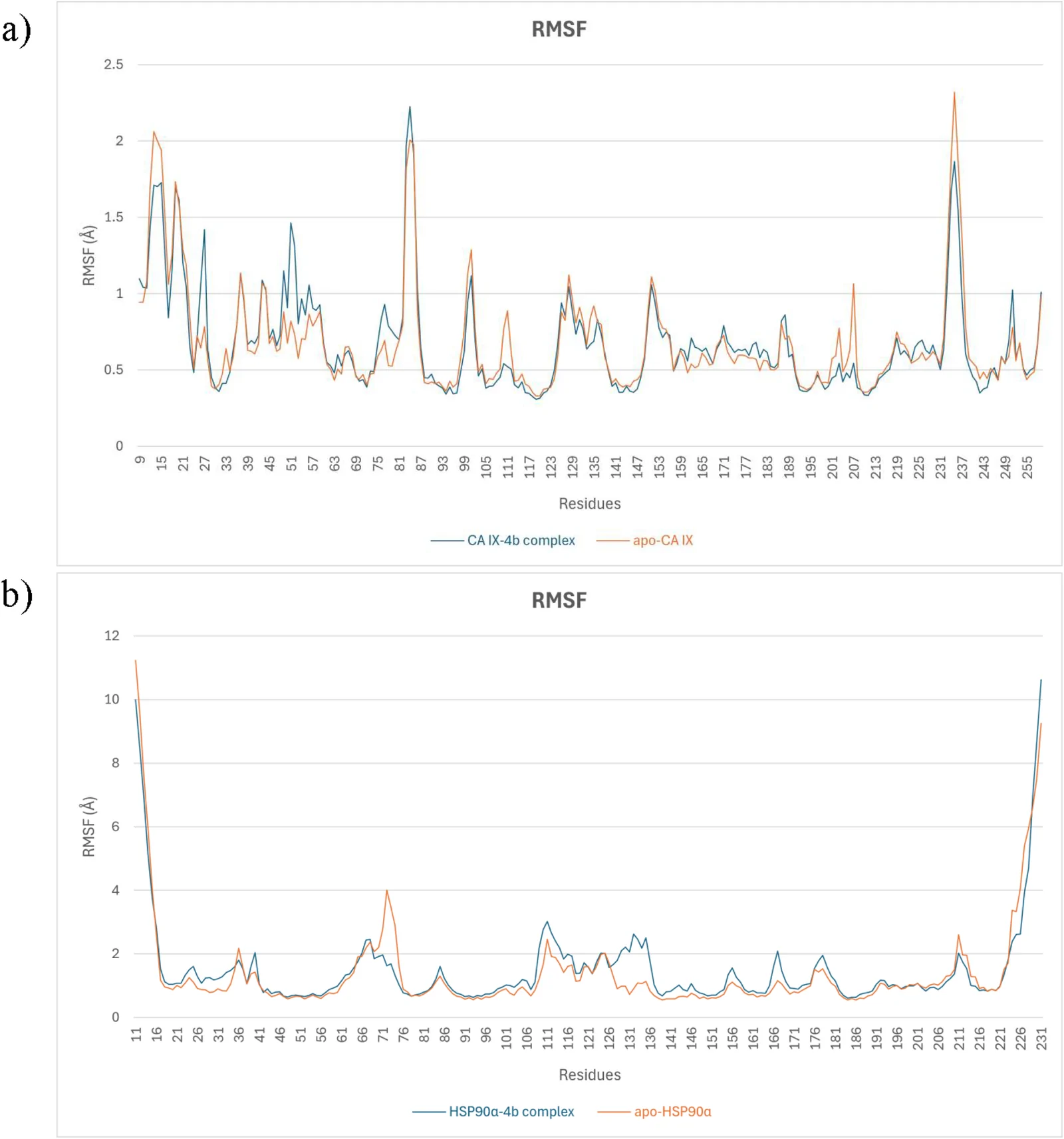
Root-mean-square fluctuation (RMSF) profiles over the 100 ns molecular dynamics simulation for the CA IX-4b complex and apo-CA IX (a), and the HSP90α-4b complex and apo-HSP90α (b).

The radius of gyration (*R*_g_) and solvent-accessible surface area (SASA) were evaluated to assess the overall compactness and solvent exposure of both targets (Fig. 13a and b). For CA IX, the *R*_g_ profile of the CA IX-4b complex and apo-CA IX were almost indistinguishable throughout the simulation, while the SASA profile for the CA IX-4b complex (mean 119.44 nm^2^) was slightly lower than that of the apo protein (mean 120.32 nm^2^), which is consistent with the partial occlusion of the active-site entrance upon compound 4b binding. For HSP90α, both the HSP90α-4b complex and apo-HSP90α systems showed stable and comparable *R*_g_ and SASA profiles throughout the 100 ns simulations, with SASA values that were closely similar, reflecting the partial accommodation of compound 4b at the entrance of the ATP-binding pocket rather than deep burial within the cavity. In a nutshell, these findings indicate that the binding of compound 4b didn't destabilize the overall structure of either target (Fig. 13a and b).

**Fig. 13:**
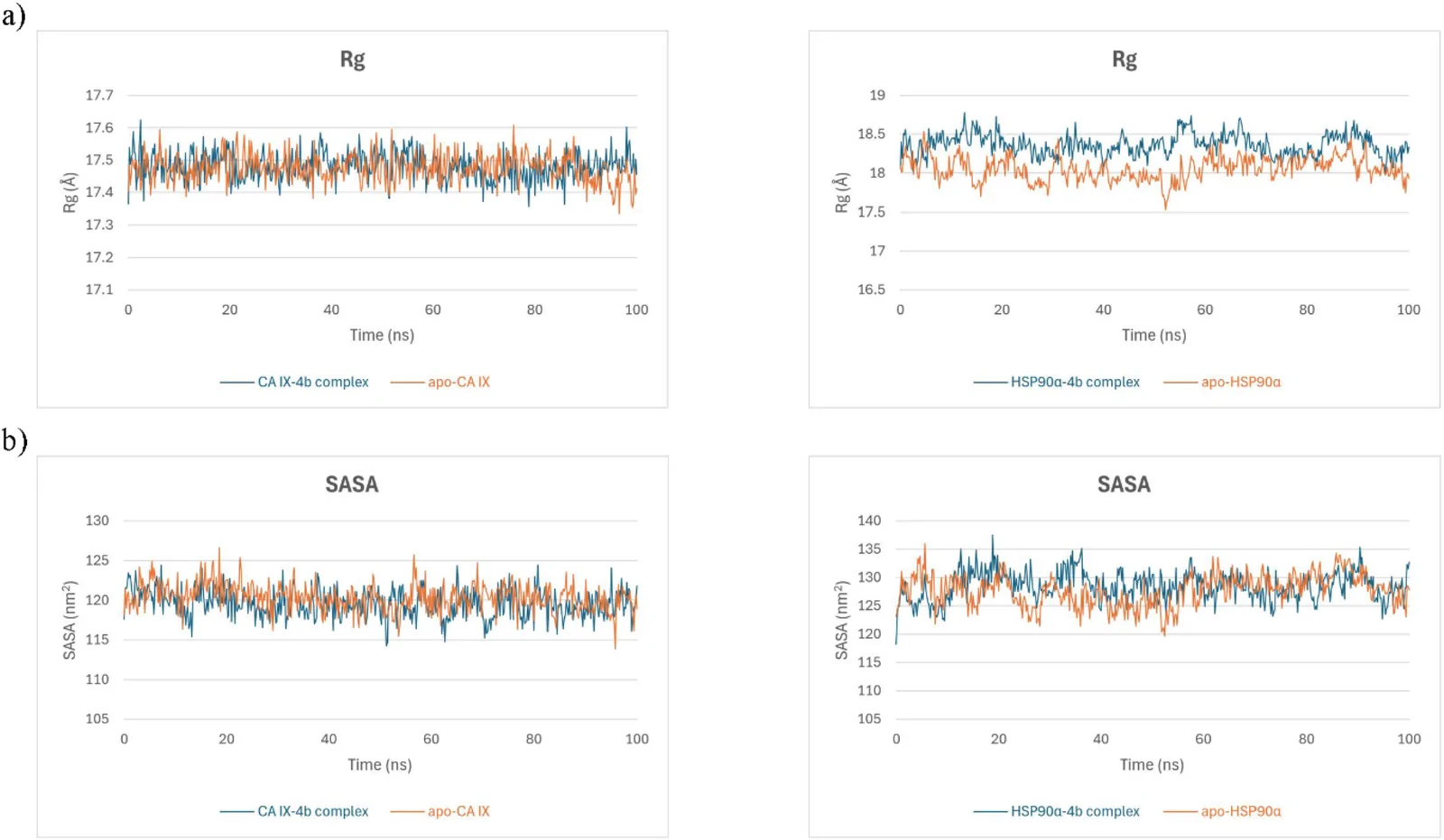
Radius of gyration (*R*_g_) (a) and solvent-accessible surface area (SASA) (b) profiles over the 100 ns molecular dynamics simulations for the CA IX-4b complex and apo-CA IX (left), and the HSP90α-4b complex and apo-HSP90α (right).

The MM/PBSA binding free energy calculations showed a favorable binding free energy for the CA IX-4b complex (Δ*G*_bind_ = −83.09 ± 0.28 kcal mol^−1^), primarily driven by electrostatic interactions (−153.10 ± 0.50 kcal mol^−1^). Similarly, the HSP90α-4b complex exhibited a favorable binding free energy (−21.75 ± 0.32 kcal mol^−1^), predominantly driven by van der Waals interactions (−33.65 ± 0.30 kcal mol^−1^). Collectively, these results indicate the energetically favorable complex formation for both targets (Table 8).

**Table 8:** MM/PBSA binding free energy components for the CA IX-4b and HSP90α-4b complexes (kcal mol^−1^)

Energy component (kcal mol^−1^)	Δ*G* (MM/PBSA) ± SE	
	CA IX-4b	HSP90α-4b
Electrostatic energy	−153.10 ± 0.50	−10.31 ± 0.41
van der Waals energy	−26.93 ± 0.22	−33.65 ± 0.30
Polar solvation energy	101.58 ± 0.43	26.73 ± 0.36
Nonpolar solvation energy	−4.66 ± 0.01	−4.52 ± 0.02
Solvation free energy	−180.02 ± 0.49	−43.96 ± 0.50
Gas-phase interaction energy	96.93 ± 0.43	22.21 ± 0.35
Overall MM/PBSA binding free energy	−83.09 ± 0.28	−21.75 ± 0.32

## Conclusion

A novel series of alkyl coumarin-tethered phenyl quinazolinones was successfully designed and synthesized as hypoxia-targeted dual hCA IX/HSP90α inhibitors. The synthesized hybrids exhibited potent, highly selective inhibition of hCA IX while maintaining negligible activity against the off-target hCA I and II isoforms. Among them, compound 4b emerged as the lead derivative, exhibiting nanomolar hCA IX inhibition (*K*_I_ = 51.7 nM), exceptional selectivity (>1934-fold over hCA I and hCA II), and potent HSP90α inhibitory activity (IC_50_ = 13.21 nM). Moreover, 4b preferentially suppressed hypoxic breast cancer cells, sensitized them to DOX treatment, and induced p53-mediated mitochondrial apoptosis. Notably, 4b exhibited consistently greater antiproliferative activity than 4a in both MCF-7 and MDA-MB-231 cells under normoxic and hypoxic conditions, further supporting its selection as the lead compound. Although these two cell lines represent biologically distinct breast cancer models, the present findings are insufficient to establish subtype-independent efficacy, and broader evaluation across additional breast cancer molecular subtypes is warranted. Molecular docking, molecular dynamics simulations, and *in silico* ADME/toxicity studies further supported its favorable binding behavior and druglike properties. Collectively, these findings demonstrate that rationally engineered alkyl coumarin-tethered phenyl quinazolinones can combine potent and selective biochemical inhibition of hCA IX and HSP90α with promising anticancer activity under hypoxic conditions, supporting further investigation of dual hCA IX/HSP90α inhibition as a potential strategy for combating breast cancer chemoresistance. However, direct mechanistic confirmation that the observed cellular effects are specifically mediated by simultaneous inhibition of hCA IX and HSP90α will require dedicated target-validation studies, such as genetic knockdown or analysis of cellular target engagement and downstream signaling. In addition, the absence of *in vivo* validation represents a limitation of the present study. Further mechanistic and *in vivo* investigations are therefore warranted to establish the target dependence, antitumor efficacy, safety, pharmacokinetic behavior, and target engagement of the lead compound 4b.

## Materials and methods

### Chemistry

The NRC, Thermo Fisher, SRL, Sigma-Aldrich, Alfa Aesar, or Merck Company provided the materials, which were utilized exactly as supplied. Every synthetic procedure was carried out at predetermined temperatures and under specified environmental conditions. Melting points were determined using the open capillary method with a Stuart SMP10, and the findings were reported without correction. The reactions were monitored using thin-layer chromatography (TLC) with *n*-hexane/ethyl acetate (2 : 1) as the developing system at 254 nm. The elemental analysis was performed using a PerkinElmer 2400 CHNS analyzer. A JEOL NMR spectrometer was used to record ^1^H NMR and ^13^C NMR-DEPTQ spectra at 500 MHz and 125 MHz, respectively, using DMSO-*d*_6_ as the solvent. A Bruker Maxis Impact Quadrupole Time-of-Flight (QTOF) spectrometer was used to obtain high-resolution mass spectra. According to HPLC analysis, each ingredient is more than 95% pure. The ZORBAX Eclipse plus C18 (150 × 4.6 mm, 3.5 µm) column was utilized in the HPLC. Acetonitrile (ACN) and phosphate buffer (pH 6.32) in varying ratios made up the mobile phase, which had a flow rate of 1.5 mL min^−1^ and a run time that doubled the elution time of each compound. The file contains the spectral data and their interpretation. Detailed synthetic procedures and characterization data for all intermediate compounds (1a–d, 2a–c, and 3a–c) are described in the SI.

### General steps for the preparation of (4a–l)

To synthesize target compound (4a), a mixture of 2-mercapto-3-phenylquinazolin-4(3*H*)-one (1a) (254 mg, 1 mmol), 7-(3-chloropropoxy)-4-methyl-2*H*-chromen-2-one (3a) (252 mg, 1 mmol), and anhydrous potassium carbonate (208 mg, 1.5 mmol) was refluxed in absolute ethanol (15 mL) at 80 °C for 48 h. The reaction mixture was poured into ice-cold water and then neutralized with glacial acetic acid to a pH of ∼5. After neutralization, the mixture was filtered. The solid obtained was washed with distilled water several times, then with absolute ethanol, diethyl ether, and finally dried to furnish compound (4a).

#### 2-((3-((4-Methyl-2-oxo-2*H*-chromen-7-yl)oxy)propyl)thio)-3-phenylquinazolin-4(3*H*)-one (4a)

White powder (68%). Mp: 176 °C. HPLC 99.90% purity (ACN/phosphate buffer, 51 : 49, *R*_T_ = 7.38 min). ^1^H NMR (500 MHz, DMSO-*d*_6_) *δ* (ppm): 2.09 (2H, s, OCH_2_**CH**_**2**_CH_2_S), 2.34 (3H, s, CH_3_), 3.23 (2H, s, OCH_2_CH_2_**CH**_**2**_S), 4.13 (2H, s, O**CH**_**2**_CH_2_CH_2_S), 6.16 (1H, s, 3-H_coumarin_), 6.91 (2H, s, H_Ar_), 7.42 (4H, s, H_Ar_), 7.51 (3H, s, H_Ar_), 7.61 (1H, s, H_Ar_), 7.72 (1H, s, H_Ar_), 8.01 (1H, s, H_Ar_). ^13^C NMR-DEPTQ (125 MHz, DMSO-*d*_6_) spectrum; CH and CH_3_ [positive (↑)], C_Q_ and CH_2_ [negative (↓)], revealed the following signals at *δ*: 18.68 (CH_3_ ↑), 28.35 (OCH_2_**CH**_**2**_CH_2_S ↓), 29.01 (OCH_2_CH_2_**CH**_**2**_S ↓), 67.38 (O**CH**_**2**_CH_2_CH_2_S ↓), 101.71 (CH_Ar_ ↑) (2C), 111.63 (3-C_coumarin_ ↑), 113.04 (CH_Ar_ ↑), 113.62 (C_Q_ ↓), 120.06 (C_Q_ ↓), 126.40 (CH_Ar_ ↑), 126.54 (CH_Ar_ ↑), 127.01 (CH_Ar_ ↑) (2C), 129.99 (CH_Ar_ ↑) (2C), 130.34 (CH_Ar_ ↑), 135.27 (CH_Ar_ ↑) (2C), 136.53 (C_Q_ ↓), 147.75 (C_Q_ ↓), 153.99 (C_Q_ ↓), 155.25 (C_Q_ ↓), 157.66 (C_Q_ ↓), 160.70 (C_Q_ ↓), 161.31 (C_Q_ ↓), 161.98 (C_Q_ ↓). HRMS (ESI/Q-TOF) *m*/*z* calculated for C_27_H_22_N_2_NaO_4_S^+^ [M + Na]^+^ 493.1192, found 493.1199. Anal. calcd for C_27_H_22_N_2_O_4_S: C, 68.92; H, 4.71; N, 5.95; S, 6.81. Found: C, 69.11; H, 4.69; N, 5.90; S, 6.80%.

#### 2-((3-((2-Oxo-4-propyl-2*H*-chromen-7-yl)oxy)propyl)thio)-3-phenylquinazolin-4(3*H*)-one (4b)

Compound (4b) was prepared following the procedure adopted for (4a), employing 2-mercapto-3-phenylquinazolin-4(3*H*)-one (1a) and 7-(3-chloropropoxy)-4-propyl-2*H*-chromen-2-one (3b). White powder (50%). Mp: 168 °C. HPLC 95.77% purity (ACN/phosphate buffer, 60 : 40, *R*_T_ = 6.74 min); ^1^H NMR (500 MHz, DMSO-*d*_6_) *δ* (ppm): 0.93 (3H, s, CH_2_CH_2_**CH**_**3**_), 1.58 (2H, s, CH_2_**CH**_**2**_CH_3_), 2.09 (2H, s, OCH_2_**CH**_**2**_CH_2_S), 2.69 (2H, s, **CH**_**2**_CH_2_CH_3_), 3.23 (2H, s, OCH_2_CH_2_**CH**_**2**_S), 4.13 (2H, s, O**CH**_**2**_CH_2_CH_2_S), 6.11 (1H, s, 3-H_coumarin_), 6.92 (2H, s, H_Ar_), 7.41 (4H, s, H_Ar_), 7.51 (3H, s, H_Ar_), 7.71 (2H, s, H_Ar_), 8.00 (1H, s, H_Ar_). ^13^C NMR-DEPTQ (125 MHz, DMSO-*d*_6_) spectrum; CH and CH_3_ [positive (↑)], C_Q_ and CH_2_ [negative (↓)], revealed the following signals at *δ*: 14.23 (CH_2_CH_2_**CH**_**3**_ ↑), 21.91 (CH_2_**CH**_**2**_CH_3_ ↓), 28.35 (OCH_2_**CH**_**2**_CH_2_S ↓), 29.00 (OCH_2_CH_2_**CH**_**2**_S ↓), 33.36 (**CH**_**2**_CH_2_CH_3_ ↓), 67.35 (O**CH**_**2**_CH_2_CH_2_S ↓), 101.92 (CH_Ar_ ↑), 110.74 (3-C_coumarin_ ↑), 112.81 (C_Q_ ↓), 113.11 (CH_Ar_ ↑) (2C), 120.06 (C_Q_ ↓), 126.39 (CH_Ar_ ↑), 126.53 (CH_Ar_ ↑), 126.76 (CH_Ar_ ↑) (2C), 127.03 (CH_Ar_ ↑), 129.99 (CH_Ar_ ↑) (2C), 130.34 (CH_Ar_ ↑), 135.26 (CH_Ar_ ↑), 136.53 (C_Q_ ↓), 147.75 (C_Q_ ↓), 155.54 (C_Q_ ↓), 157.34 (C_Q_ ↓), 157.66 (C_Q_ ↓), 160.84 (C_Q_ ↓), 161.31 (C_Q_ ↓), 161.85 (C_Q_ ↓). HRMS (ESI/Q-TOF) *m*/*z* calculated for C_29_H_26_N_2_NaO_4_S^+^ [M + Na]^+^ 521.1505, found 521.1515. Anal. calcd for C_29_H_26_N_2_O_4_S: C, 69.86; H, 5.26; N, 5.62; S, 6.43. Found: C, 69.54; H, 5.25; N, 5.66; S, 6.39%.

#### 2-((3-((4-Isopropyl-2-oxo-2*H*-chromen-7-yl)oxy)propyl)thio)-3-phenylquinazolin-4(3*H*)-one (4c)

Compound (4c) was prepared following the procedure adopted for (4a), employing 2-mercapto-3-phenylquinazolin-4(3*H*)-one (1a) and 7-(3-chloropropoxy)-4-isopropyl-2*H*-chromen-2-one (3c). White powder (55%). Mp: 179 °C. HPLC 96.40% purity (ACN/phosphate buffer, 60 : 40, *R*_T_ = 5.16 min); ^1^H NMR (500 MHz, DMSO-*d*_6_) *δ* (ppm): 1.19 (6H, s, CH(**CH**_**3**_)_2_), 2.09 (2H, s, OCH_2_**CH**_**2**_CH_2_S), 3.24 (3H, s, OCH_2_CH_2_**CH**_**2**_S and **CH**(CH_3_)_2_), 4.14 (2H, s, O**CH**_**2**_CH_2_CH_2_S), 6.10 (1H, s, 3-H_coumarin_), 6.93 (2H, s, H_Ar_), 7.41 (4H, s, H_Ar_), 7.51 (3H, s, H_Ar_), 7.71 (2H, s, H_Ar_), 8.00 (1H, s, H_Ar_). ^13^C NMR-DEPTQ (125 MHz, DMSO-*d*_6_) spectrum; CH and CH_3_ [positive (↑)], C_Q_ and CH_2_ [negative (↓)], revealed the following signals at *δ*: 22.08 (CH(**CH**_**3**_)_2_ ↑) (2C), 28.35 (OCH_2_**CH**_**2**_CH_2_S ↓), 28.49 (**CH**(CH_3_)_2_ ↑), 29.00 (OCH_2_CH_2_**CH**_**2**_S ↓), 67.34 (O**CH**_**2**_CH_2_CH_2_S ↓), 102.06 (CH_Ar_ ↑), 107.88 (CH_Ar_ ↑), 112.22 (C_Q_ ↓), 113.14 (3-C_coumarin_ ↑), 120.04 (C_Q_ ↓), 126.38 (CH_Ar_ ↑), 126.42 (CH_Ar_ ↑), 126.51 (CH_Ar_ ↑), 127.02 (CH_Ar_ ↑), 129.96 (CH_Ar_ ↑) (2C), 130.01 (CH_Ar_ ↑) (2C), 130.34 (CH_Ar_ ↑), 135.25 (CH_Ar_ ↑), 136.51 (C_Q_ ↓), 147.74 (C_Q_ ↓), 155.62 (C_Q_ ↓), 157.65 (C_Q_ ↓), 161.24 (C_Q_ ↓) (2C), 161.73 (C_Q_ ↓), 163.03 (C_Q_ ↓). HRMS (ESI/Q-TOF) *m*/*z* calculated for C_29_H_26_KN_2_O_4_S^+^ [M + K]^+^ 537.1245, found 537.1265. Anal. calcd for C_29_H_26_N_2_O_4_S: C, 69.86; H, 5.26; N, 5.62; S, 6.43. Found: C, 70.04; H, 5.19; N, 5.64; S, 6.45%.

#### 7-Chloro-2-((3-((4-methyl-2-oxo-2*H*-chromen-7-yl)oxy)propyl)thio)-3-phenylquinazolin-4(3*H*)-one (4d)

Compound (4d) was prepared following the procedure adopted for (4a), employing 7-chloro-2-mercapto-3-phenylquinazolin-4(3*H*)-one (1b) and 7-(3-chloropropoxy)-4-methyl-2*H*-chromen-2-one (3a). White powder (80%). Mp: 207 °C. HPLC 99.26% purity (ACN/phosphate buffer, 60 : 40, *R*_T_ = 5.08 min); ^1^H NMR (500 MHz, DMSO-*d*_6_) *δ* (ppm): 2.08 (2H, s, OCH_2_**CH**_**2**_CH_2_S), 2.34 (3H, s, CH_3_), 3.23 (2H, s, OCH_2_CH_2_**CH**_**2**_S), 4.13 (2H, s, O**CH**_**2**_CH_2_CH_2_S), 6.16 (1H, s, 3-H_coumarin_), 6.91 (2H, s, H_Ar_), 7.30 (1H, s, H_Ar_), 7.42 (3H, s, H_Ar_), 7.51 (3H, s, H_Ar_), 7.61 (1H, s, H_Ar_), 7.96 (1H, s, H_Ar_). ^13^C NMR-DEPTQ (125 MHz, DMSO-*d*_6_) spectrum; CH and CH_3_ [positive (↑)], C_Q_ and CH_2_ [negative (↓)], revealed the following signals at *δ*: 18.68 (CH_3_ ↑), 28.68 (OCH_2_**CH**_**2**_CH_2_S ↓), 29.05 (OCH_2_CH_2_**CH**_**2**_S ↓), 67.23 (O**CH**_**2**_CH_2_CH_2_S ↓), 101.67 (CH_Ar_ ↑), 111.61 (3-C_coumarin_ ↑), 112.93 (CH_Ar_ ↑), 113.60 (C_Q_ ↓), 118.90 (C_Q_ ↓), 125.53 (CH_Ar_ ↑), 126.43 (CH_Ar_ ↑), 126.97 (CH_Ar_ ↑), 129.05 (CH_Ar_ ↑), 129.88 (CH_Ar_ ↑) (2C), 130.04 (CH_Ar_ ↑) (2C), 130.45 (CH_Ar_ ↑), 136.30 (C_Q_ ↓), 139.75 (C_Q_ ↓), 148.64 (C_Q_ ↓), 153.99 (C_Q_ ↓), 155.25 (C_Q_ ↓), 159.73 (C_Q_ ↓), 160.71 (C_Q_ ↓) (2C), 161.99 (C_Q_ ↓). HRMS (ESI/Q-TOF) *m*/*z* calculated for C_27_H_21_^35^ClKN_2_O_4_S^+^ [M + K]^+^ 543.0542, found 543.0558, and calculated for C_27_H_21_^37^ClKN_2_O_4_S^+^ [M + K]^+^ (M + 2 isotopic peak) 545.0512, found 545.0536. Anal. calcd for C_27_H_21_ClN_2_O_4_S: C, 64.22; H, 4.19; N, 5.55; S, 6.35. Found: C, 63.99; H, 4.11; N, 5.49; S, 6.33%.

#### 7-Chloro-2-((3-((2-oxo-4-propyl-2*H*-chromen-7-yl)oxy)propyl)thio)-3-phenylquinazolin-4(3*H*)-one (4e)

Compound (4e) was prepared following the procedure adopted for (4a), employing 7-chloro-2-mercapto-3-phenylquinazolin-4(3*H*)-one (1b) and 7-(3-chloropropoxy)-4-propyl-2*H*-chromen-2-one (3b). White powder (85%). Mp: 178 °C. HPLC 99.40% purity (ACN/phosphate buffer, 65 : 35, *R*_T_ = 7.43 min); ^1^H NMR (500 MHz, DMSO-*d*_6_) *δ* (ppm): 0.93 (3H, s, CH_2_CH_2_**CH**_**3**_), 1.58 (2H, s, CH_2_**CH**_**2**_CH_3_), 2.09 (2H, s, OCH_2_**CH**_**2**_CH_2_S), 2.68 (2H, s, **CH**_**2**_CH_2_CH_3_), 3.24 (2H, s, OCH_2_CH_2_**CH**_**2**_S), 4.14 (2H, s, O**CH**_**2**_CH_2_CH_2_S), 6.10 (1H, s, 3-H_coumarin_), 6.91 (2H, s, H_Ar_), 7.32 (1H, s, H_Ar_), 7.42 (3H, s, H_Ar_), 7.52 (3H, s, H_Ar_), 7.65 (1H, s, H_Ar_), 7.97 (1H, s, H_Ar_). ^13^C NMR-DEPTQ (125 MHz, DMSO-*d*_6_) spectrum; CH and CH_3_ [positive (↑)], C_Q_ and CH_2_ [negative (↓)], revealed the following signals at *δ*: 14.23 (CH_2_CH_2_**CH**_**3**_↑), 21.84 (CH_2_**CH**_**2**_CH_3_ ↓), 28.68 (OCH_2_**CH**_**2**_CH_2_S ↓), 29.05 (OCH_2_CH_2_**CH**_**2**_S ↓), 33.36 (**CH**_**2**_CH_2_CH_3_ ↓), 67.22 (O**CH**_**2**_CH_2_CH_2_S ↓), 101.93 (CH_Ar_ ↑), 110.70 (3-C_coumarin_ ↑), 112.80 (C_Q_ ↓), 112.92 (CH_Ar_ ↑), 118.88 (C_Q_ ↓), 125.55 (CH_Ar_ ↑), 126.42 (CH_Ar_ ↑), 126.71 (CH_Ar_ ↑), 129.05 (CH_Ar_ ↑), 129.87 (CH_Ar_ ↑) (2C), 130.05 (CH_Ar_ ↑) (2C), 130.46 (CH_Ar_ ↑), 136.30 (C_Q_ ↓), 139.75 (C_Q_ ↓), 148.64 (C_Q_ ↓), 155.52 (C_Q_ ↓), 157.31 (C_Q_ ↓), 159.73 (C_Q_ ↓), 160.73 (C_Q_ ↓), 160.84 (C_Q_ ↓), 161.86 (C_Q_ ↓). HRMS (ESI/Q-TOF) *m*/*z* calculated for C_29_H_25_^35^ClN_2_NaO_4_S^+^ [M + Na]^+^ 555.1115, found 555.1125, and calculated for C_29_H_25_^37^ClN_2_NaO_4_S^+^ [M + Na]^+^ (M + 2 isotopic peak) 557.1086, found 557.1114. Anal. calcd for C_29_H_25_ClN_2_O_4_S: C, 65.35; H, 4.73; N, 5.26; S, 6.01. Found: C, 65.09; H, 4.76; N, 5.22; S, 5.97%.

#### 7-Chloro-2-((3-((4-isopropyl-2-oxo-2*H*-chromen-7-yl)oxy)propyl)thio)-3-phenylquinazolin-4(3*H*)-one (4f)

Compound (4f) was prepared following the procedure adopted for (4a), employing 7-chloro-2-mercapto-3-phenylquinazolin-4(3*H*)-one (1b) and 7-(3-chloropropoxy)-4-isopropyl-2*H*-chromen-2-one (3c). White powder (70%). Mp: 182 °C. HPLC 98.37% purity (ACN/phosphate buffer, 65 : 35, *R*_T_ = 7.16 min). ^1^H NMR (500 MHz, DMSO-*d*_6_) *δ* (ppm): 1.17 (6H, s, CH(**CH**_**3**_)_2_), 2.07 (2H, s, OCH_2_**CH**_**2**_CH_2_S), 3.23 (3H, s, OCH_2_CH_2_**CH**_**2**_S and **CH**(CH_3_)_2_), 4.13 (2H, s, O**CH**_**2**_CH_2_CH_2_S), 6.09 (1H, s, 3-H_coumarin_), 6.91 (2H, s, H_Ar_), 7.41 (4H, s, H_Ar_), 7.51 (3H, s, H_Ar_), 7.70 (1H, s, H_Ar_), 7.94 (1H, s, H_Ar_). ^13^C NMR-DEPTQ (125 MHz, DMSO-*d*_6_) spectrum; CH and CH_3_ [positive (↑)], C_Q_ and CH_2_ [negative (↓)], revealed the following signals at *δ*: 22.07 (CH(**CH**_**3**_)_2_ ↑) (2C), 28.49 (**CH**(CH_3_)_2_ ↑), 28.71 (OCH_2_**CH**_**2**_CH_2_S ↓), 29.05 (OCH_2_CH_2_**CH**_**2**_S ↓), 67.20 (O**CH**_**2**_CH_2_CH_2_S ↓), 102.11 (CH_Ar_ ↑), 107.88 (CH_Ar_ ↑), 112.23 (C_Q_ ↓), 112.91 (3-C_coumarin_ ↑), 118.90 (C_Q_ ↓), 125.53 (CH_Ar_ ↑), 126.39 (CH_Ar_ ↑) (2C), 129.04 (CH_Ar_ ↑), 129.88 (CH_Ar_ ↑) (2C), 130.03 (CH_Ar_ ↑) (2C), 130.44 (CH_Ar_ ↑), 136.31 (C_Q_ ↓), 139.71 (C_Q_ ↓), 148.64 (C_Q_ ↓), 155.51 (C_Q_ ↓), 159.73 (C_Q_ ↓), 160.70 (C_Q_ ↓), 161.22 (C_Q_ ↓), 161.75 (C_Q_ ↓), 162.99 (C_Q_ ↓). HRMS (ESI/Q-TOF) *m*/*z* calculated for C_29_H_25_^35^ClKN_2_O_4_S^+^ [M + K]^+^ 571.0855, found 571.0864, and calculated for C_29_H_25_^37^ClKN_2_O_4_S^+^ [M + K]^+^ (M + 2 isotopic peak) 573.0825, found 573.0843. Anal. calcd for C_29_H_25_ClN_2_O_4_S: C, 65.35; H, 4.73; N, 5.26; S, 6.01. Found: C, 65.04; H, 4.69; N, 5.29; S, 6.04%.

#### 3-(4-Chlorophenyl)-2-((3-((4-methyl-2-oxo-2*H*-chromen-7-yl)oxy)propyl)thio)quinazolin-4(3*H*)-one (4g)

Compound (4g) was prepared following the procedure adopted for (4a), employing 3-(4-chlorophenyl)-2-mercaptoquinazolin-4(3*H*)-one (1c) and 7-(3-chloropropoxy)-4-methyl-2*H*-chromen-2-one (3a). White powder (62%). Mp: 177 °C. HPLC 97.52% purity (ACN/phosphate buffer, 50 : 50, *R*_T_ = 5.32 min); ^1^H NMR (500 MHz, DMSO-*d*_6_) *δ* (ppm): 2.10 (2H, s, OCH_2_**CH**_**2**_CH_2_S), 2.34 (3H, s, CH_3_), 3.25 (2H, s, OCH_2_CH_2_**CH**_**2**_S), 4.14 (2H, s, O**CH**_**2**_CH_2_CH_2_S), 6.17 (1H, s, 3-H_coumarin_), 6.92 (2H, s, H_Ar_), 7.49 (4H, s, H_Ar_), 7.61 (3H, s, H_Ar_), 7.72 (1H, s, H_Ar_), 8.00 (1H, s, H_Ar_). ^13^C NMR-DEPTQ (125 MHz, DMSO-*d*_6_) spectrum; CH and CH_3_ [positive (↑)], C_Q_ and CH_2_ [negative (↓)], revealed the following signals at *δ*: 18.68 (CH_3_ ↑), 28.33 (OCH_2_**CH**_**2**_CH_2_S ↓), 29.03 (OCH_2_CH_2_**CH**_**2**_S ↓), 67.33 (O**CH**_**2**_CH_2_CH_2_S ↓), 101.69 (CH_Ar_ ↑), 111.63 (3-C_coumarin_ ↑), 113.07 (CH_Ar_ ↑), 113.62 (C_Q_ ↓), 120.05 (C_Q_ ↓), 126.45 (CH_Ar_ ↑), 126.55 (CH_Ar_ ↑), 126.99 (CH_Ar_ ↑), 127.03 (CH_Ar_ ↑), 130.10 (CH_Ar_ ↑) (2C), 132.03 (CH_Ar_ ↑) (2C), 135.06 (C_Q_ ↓), 135.33 (CH_Ar_ ↑), 135.48 (C_Q_ ↓), 147.74 (C_Q_ ↓), 154.00 (C_Q_ ↓), 155.25 (C_Q_ ↓), 157.29 (C_Q_ ↓), 160.71 (C_Q_ ↓), 161.29 (C_Q_ ↓), 161.97 (C_Q_ ↓). HRMS (ESI/Q-TOF) *m*/*z* calculated for C_27_H_21_^35^ClKN_2_O_4_S^+^ [M + K]^+^ 543.0542, found 543.0548, and calculated for C_27_H_21_^37^ClKN_2_O_4_S^+^ [M + K]^+^ (M + 2 isotopic peak) 545.0512, found 545.0517. Anal. calcd for C_27_H_21_ClN_2_O_4_S: C, 64.22; H, 4.19; N, 5.55; S, 6.35. Found: C, 63.96; H, 4.24; N, 5.58; S, 6.36%.

#### 3-(4-Chlorophenyl)-2-((3-((2-oxo-4-propyl-2*H*-chromen-7-yl)oxy)propyl)thio)quinazolin-4(3*H*)-one (4h)

Compound (4h) was prepared following the procedure adopted for (4a), employing 3-(4-chlorophenyl)-2-mercaptoquinazolin-4(3*H*)-one (1c) and 7-(3-chloropropoxy)-4-propyl-2*H*-chromen-2-one (3b). White powder (80%). Mp: 168 °C. HPLC 98.49% purity (ACN/phosphate buffer, 65 : 35, *R*_T_ = 6.00 min); ^1^H NMR (500 MHz, DMSO-*d*_6_) *δ* (ppm): 0.91 (3H, s, CH_2_CH_2_**CH**_**3**_), 1.57 (2H, s, CH_2_**CH**_**2**_CH_3_), 2.09 (2H, s, OCH_2_**CH**_**2**_CH_2_S), 2.67 (2H, s, **CH**_**2**_CH_2_CH_3_), 3.23 (2H, s, OCH_2_CH_2_**CH**_**2**_S), 4.13 (2H, s, O**CH**_**2**_CH_2_CH_2_S), 6.10 (1H, s, 3-H_coumarin_), 6.90 (2H, s, H_Ar_), 7.46–7.99 (9H, m, H_Ar_). ^13^C NMR-DEPTQ (125 MHz, DMSO-*d*_6_) spectrum; CH and CH_3_ [positive (↑)], C_Q_ and CH_2_ [negative (↓)], revealed the following signals at *δ*: 14.22 (CH_2_CH_2_**CH**_**3**_ ↑), 21.90 (CH_2_**CH**_**2**_CH_3_ ↓), 28.33 (OCH_2_**CH**_**2**_CH_2_S ↓), 29.04 (OCH_2_CH_2_**CH**_**2**_S ↓), 33.36 (**CH**_**2**_CH_2_CH_3_ ↓), 67.30 (O**CH**_**2**_CH_2_CH_2_S ↓), 101.91 (CH_Ar_ ↑), 110.73 (3-C_coumarin_ ↑), 112.80 (C_Q_ ↓), 113.12 (CH_Ar_ ↑), 120.05 (C_Q_ ↓), 126.42 (CH_Ar_ ↑), 126.53 (CH_Ar_ ↑), 126.74 (CH_Ar_ ↑), 127.03 (CH_Ar_ ↑), 130.09 (CH_Ar_ ↑) (2C), 132.03 (CH_Ar_ ↑) (2C), 135.05 (C_Q_ ↓), 135.31 (CH_Ar_ ↑), 135.48 (C_Q_ ↓) (2C), 147.72 (C_Q_ ↓), 157.30 (C_Q_ ↓) (2C), 160.82 (C_Q_ ↓), 161.28 (C_Q_ ↓), 161.83 (C_Q_ ↓). HRMS (ESI/Q-TOF) *m*/*z* calculated for C_29_H_25_^35^ClKN_2_O_4_S^+^ [M + K]^+^ 571.0855, found 571.0861, and calculated for C_29_H_25_^37^ClKN_2_O_4_S^+^ [M + K]^+^ (M + 2 isotopic peak) 573.0825, found 573.0842. Anal. calcd for C_29_H_25_ClN_2_O_4_S: C, 65.35; H, 4.73; N, 5.26; S, 6.01. Found: C, 65.65; H, 4.65; N, 5.31; S, 6.07%.

#### 3-(4-Chlorophenyl)-2-((3-((4-isopropyl-2-oxo-2*H*-chromen-7-yl)oxy)propyl)thio)quinazolin-4(3*H*)-one (4i)

Compound (4i) was prepared following the procedure adopted for (4a), employing 3-(4-chlorophenyl)-2-mercaptoquinazolin-4(3*H*)-one (1c) and 7-(3-chloropropoxy)-4-isopropyl-2*H*-chromen-2-one (3c). White powder (65%). Mp: 165 °C. HPLC 98.41% purity (ACN/phosphate buffer, 65 : 35, *R*_T_ = 6.44 min); ^1^H NMR (500 MHz, DMSO-*d*_6_) *δ* (ppm): 1.19 (6H, s, CH(**CH**_**3**_)_2_), 2.10 (2H, s, OCH_2_**CH**_**2**_CH_2_S), 3.25 (3H, s, OCH_2_CH_2_**CH**_**2**_S and **CH**(CH_3_)_2_), 4.14 (2H, s, O**CH**_**2**_CH_2_CH_2_S), 6.11 (1H, s, 3-H_coumarin_), 6.93 (2H, s, H_Ar_), 7.41 (2H, s, H_Ar_), 7.50 (2H, s, H_Ar_), 7.60 (2H, s, H_Ar_), 7.72 (2H, s, H_Ar_), 8.00 (1H, s, H_Ar_). ^13^C NMR-DEPTQ (125 MHz, DMSO-*d*_6_) spectrum; CH and CH_3_ [positive (↑)], C_Q_ and CH_2_ [negative (↓)], revealed the following signals at *δ*: 22.09 (CH(**CH**_**3**_)_2_ ↑) (2C), 28.33 (OCH_2_**CH**_**2**_CH_2_S ↓), 28.50 (**CH**(CH_3_)_2_ ↑), 29.03 (OCH_2_CH_2_**CH**_**2**_S ↓), 67.30 (O**CH**_**2**_CH_2_CH_2_S ↓), 102.07 (CH_Ar_ ↑), 107.90 (CH_Ar_ ↑), 112.22 (C_Q_ ↓), 113.16 (3-C_coumarin_ ↑), 120.03 (C_Q_ ↓), 126.42 (CH_Ar_ ↑)) (2C), 126.51 (CH_Ar_ ↑), 127.04 (CH_Ar_ ↑), 130.10 (CH_Ar_ ↑) (2C), 132.03 (CH_Ar_ ↑) (2C), 135.06 (C_Q_ ↓), 135.31 (CH_Ar_ ↑), 135.46 (C_Q_ ↓), 147.72 (C_Q_ ↓), 155.66 (C_Q_ ↓), 157.29 (C_Q_ ↓), 161.28 (C_Q_ ↓) (2C), 161.73 (C_Q_ ↓), 163.06 (C_Q_ ↓). HRMS (ESI/Q-TOF) *m*/*z* calculated for C_29_H_25_^35^ClKN_2_O_4_S^+^ [M + K]^+^ 571.0855, found 571.0864, and calculated for C_29_H_25_^37^ClKN_2_O_4_S^+^ [M + K]^+^ (M + 2 isotopic peak) 573.0825, found 573.0859. Anal. calcd for C_29_H_25_ClN_2_O_4_S: C, 65.35; H, 4.73; N, 5.26; S, 6.01. Found: C, 65.51; H, 4.77; N, 5.21; S, 6.00%.

#### 7-Chloro-3-(4-chlorophenyl)-2-((3-((4-methyl-2-oxo-2*H*-chromen-7-yl)oxy)propyl)thio) quinazolin-4(3*H*)-one (4j)

Compound (4j) was prepared following the procedure adopted for (4a), employing 7-chloro-3-(4-chlorophenyl)-2-mercaptoquinazolin-4(3*H*)-one (1d) and 7-(3-chloropropoxy)-4-methyl-2*H*-chromen-2-one (3a). White powder (61%). Mp: 188 °C. HPLC 99.28% purity (ACN/phosphate buffer, 60 : 40, *R*_T_ = 5.04 min); ^1^H NMR (500 MHz, DMSO-*d*_6_) *δ* (ppm): 2.09 (2H, s, OCH_2_**CH**_**2**_CH_2_S), 2.35 (3H, s, CH_3_), 3.25 (2H, s, OCH_2_CH_2_**CH**_**2**_S), 4.15 (2H, s, O**CH**_**2**_CH_2_CH_2_S), 6.17 (1H, s, 3-H_coumarin_), 6.92 (2H, s, H_Ar_), 7.31 (1H, s, H_Ar_), 7.40 (1H, s, H_Ar_), 7.51 (2H, s, H_Ar_), 7.61 (3H, s, H_Ar_), 7.98 (1H, s, H_Ar_).^13^C NMR-DEPTQ (125 MHz, DMSO-*d*_6_) spectrum; CH and CH_3_ [positive (↑)], C_Q_ and CH_2_ [negative (↓)], revealed the following signals at *δ*: 18.68 (CH_3_ ↑), 28.68 (OCH_2_**CH**_**2**_CH_2_S ↓), 29.07 (OCH_2_CH_2_**CH**_**2**_S ↓), 67.17 (O**CH**_**2**_CH_2_CH_2_S ↓), 101.67 (CH_Ar_ ↑), 111.61 (3-C_coumarin_ ↑), 112.98 (CH_Ar_ ↑), 113.61 (C_Q_ ↓), 118.90 (C_Q_ ↓), 125.54 (CH_Ar_ ↑), 126.48 (CH_Ar_ ↑), 126.98 (CH_Ar_ ↑), 129.05 (CH_Ar_ ↑), 130.15 (CH_Ar_ ↑) (2C), 131.94 (CH_Ar_ ↑) (2C), 135.19 (C_Q_ ↓), 135.26 (C_Q_ ↓), 139.81 (C_Q_ ↓), 148.62 (C_Q_ ↓), 154.01 (C_Q_ ↓), 155.26 (C_Q_ ↓), 159.38 (C_Q_ ↓), 160.72 (C_Q_ ↓) (2C), 161.99 (C_Q_ ↓). HRMS (ESI/Q-TOF) *m*/*z* calculated for C_27_H_20_^35^Cl^35^ClKN_2_O_4_S^+^ [M + K]^+^ 577.0152, found 577.0160, calculated for C_27_H_20_^35^Cl^37^ClKN_2_O_4_S^+^ [M + K]^+^ (M + 2 isotopic peak) 579.0122, found 579.0136, and calculated for C_27_H_20_^37^Cl^37^ClKN_2_O_4_S^+^ [M + K]^+^ (M + 4 isotopic peak) 581.0093, found 581.0119. Anal. calcd for C_27_H_20_Cl_2_N_2_O_4_S: C, 60.12; H, 3.74; N, 5.19; S, 5.94. Found: C, 59.90; H, 3.80; N, 5.22; S, 5.99%.

#### 7-Chloro-3-(4-chlorophenyl)-2-((3-((2-oxo-4-propyl-2*H*-chromen-7-yl)oxy)propyl)thio) quinazolin-4(3*H*)-one (4k)

Compound (4k) was prepared following the procedure adopted for (4a), employing 7-chloro-3-(4-chlorophenyl)-2-mercaptoquinazolin-4(3*H*)-one (1d) and 7-(3-chloropropoxy)-4-propyl-2*H*-chromen-2-one (3b). White powder (73%). Mp: 179 °C. HPLC 98.54% purity (ACN/phosphate buffer, 70 : 30, *R*_T_ = 6.70 min). ^1^H NMR (500 MHz, DMSO-*d*_6_) *δ* (ppm): 0.92 (3H, s, CH_2_CH_2_**CH**_**3**_), 1.57 (2H, s, CH_2_**CH**_**2**_CH_3_), 2.08 (2H, s, OCH_2_**CH**_**2**_CH_2_S), 2.67 (2H, s, **CH**_**2**_CH_2_CH_3_), 3.24 (2H, s, OCH_2_CH_2_**CH**_**2**_S), 4.13 (2H, s, O**CH**_**2**_CH_2_CH_2_S), 6.10 (1H, s, 3-H_coumarin_), 6.91 (2H, s, H_Ar_), 7.32 (1H, s, H_Ar_), 7.38 (1H, s, H_Ar_), 7.49 (2H, s, H_Ar_), 7.59 (2H, s, H_Ar_), 7.65 (1H, s, H_Ar_), 7.95 (1H, s, H_Ar_). ^13^C NMR-DEPTQ (125 MHz, DMSO-*d*_6_) spectrum; CH and CH_3_ [positive (↑)], C_Q_ and CH_2_ [negative (↓)], revealed the following signals at *δ*: 14.23 (CH_2_CH_2_**CH**_**3**_ ↑), 21.85 (CH_2_**CH**_**2**_CH_3_ ↓), 28.67 (OCH_2_**CH**_**2**_CH_2_S ↓), 29.08 (OCH_2_CH_2_**CH**_**2**_S ↓), 33.36 (**CH**_**2**_CH_2_CH_3_ ↓), 67.16 (O**CH**_**2**_CH_2_CH_2_S ↓), 101.93 (CH_Ar_ ↑), 110.71 (3-C_coumarin_ ↑), 112.82 (C_Q_ ↓), 112.95 (CH_Ar_ ↑), 118.89 (C_Q_ ↓), 125.56 (CH_Ar_ ↑), 126.46 (CH_Ar_ ↑), 126.70 (CH_Ar_ ↑), 129.05 (CH_Ar_ ↑), 130.14 (CH_Ar_ ↑) (2C), 131.94 (CH_Ar_ ↑) (2C), 135.18 (C_Q_ ↓), 135.26 (C_Q_ ↓), 139.80 (C_Q_ ↓), 148.62 (C_Q_ ↓), 155.53 (C_Q_ ↓), 157.30 (C_Q_ ↓), 159.38 (C_Q_ ↓), 160.70 (C_Q_ ↓), 160.83 (C_Q_ ↓), 161.85 (C_Q_ ↓). HRMS (ESI/Q-TOF) *m*/*z* calculated for C_29_H_24_^35^Cl^35^ClKN_2_O_4_S^+^ [M + K]^+^ 605.0465, found 605.0481, calculated for C_29_H_24_^35^Cl^37^ClKN_2_O_4_S^+^ [M + K]^+^ (M + 2 isotopic peak) 607.0435, found 607.0452, and calculated for C_29_H_24_^37^Cl^37^ClKN_2_O_4_S^+^ [M + K]^+^ (M + 4 isotopic peak) 609.0406, found 609.0452. Anal. calcd for C_29_H_24_Cl_2_N_2_O_4_S: C, 61.38; H, 4.26; N, 4.94; S, 5.65. Found: C, 61.69; H, 4.31; N, 4.96; S, 5.69%.

#### 7-Chloro-3-(4-chlorophenyl)-2-((3-((4-isopropyl-2-oxo-2*H*-chromen-7-yl)oxy)propyl)thio) quinazolin-4(3*H*)-one (4l)

Compound (4l) was prepared following the procedure adopted for (4a), employing 7-chloro-3-(4-chlorophenyl)-2-mercaptoquinazolin-4(3*H*)-one (1d) and 7-(3-chloropropoxy)-4-isopropyl-2*H*-chromen-2-one (3c). White powder (62%). Mp: 154 °C. HPLC 99.84% purity (ACN/phosphate buffer, 60 : 40, *R*_T_ = 15.23 min); ^1^H NMR (500 MHz, DMSO-*d*_6_) *δ* (ppm): 1.19 (6H, s, CH(**CH**_**3**_)_2_), 2.09 (2H, s, OCH_2_**CH**_**2**_CH_2_S), 3.26 (3H, s, OCH_2_CH_2_**CH**_**2**_S and **CH**(CH_3_)_2_), 4.14 (2H, s, O**CH**_**2**_CH_2_CH_2_S), 6.10 (1H, s, 3-H_coumarin_), 6.92 (2H, s, H_Ar_), 7.31 (1H, s, H_Ar_), 7.39 (1H, s, H_Ar_), 7.50 (2H, s, H_Ar_), 7.60 (2H, s, H_Ar_), 7.72 (1H, s, H_Ar_), 7.96 (1H, s, H_Ar_). ^13^C NMR-DEPTQ (125 MHz, DMSO-*d*_6_) spectrum; CH and CH_3_ [positive (↑)], C_Q_ and CH_2_ [negative (↓)], revealed the following signals at *δ*: 22.07 (CH(**CH**_**3**_)_2_ ↑) (2C), 28.51 (**CH**(CH_3_)_2_ ↑), 28.71 (OCH_2_**CH**_**2**_CH_2_S ↓), 29.06 (OCH_2_CH_2_**CH**_**2**_S ↓), 67.14 (O**CH**_**2**_CH_2_CH_2_S ↓), 102.09 (CH_Ar_ ↑), 107.87 (CH_Ar_ ↑), 112.24 (C_Q_ ↓), 112.97 (3-C_coumarin_ ↑), 118.85 (C_Q_ ↓), 125.54 (CH_Ar_ ↑), 126.39 (CH_Ar_ ↑) (2C), 127.70 (C_Q_ ↓), 129.05 (CH_Ar_ ↑), 130.16 (CH_Ar_ ↑) (2C), 131.92 (CH_Ar_ ↑) (2C), 135.23 (C_Q_ ↓) (2C), 139.79 (C_Q_ ↓), 148.60 (C_Q_ ↓), 159.38 (C_Q_ ↓), 160.72 (C_Q_ ↓), 161.27 (C_Q_ ↓), 161.76 (C_Q_ ↓), 163.05 (C_Q_ ↓). HRMS (ESI/Q-TOF) *m*/*z* calculated for C_29_H_24_^35^Cl^35^ClKN_2_O_4_S^+^ [M + K]^+^ 605.0465, found 605.0477, calculated for C_29_H_24_^35^Cl^37^ClKN_2_O_4_S^+^ [M + K]^+^ (M + 2 isotopic peak) 607.0435, found 607.0460, and calculated for C_29_H_24_^37^Cl^37^ClKN_2_O_4_S^+^ [M + K]^+^ (M + 4 isotopic peak) 609.0406, found 609.0453. Anal. calcd for C_29_H_24_Cl_2_N_2_O_4_S: C, 61.38; H, 4.26; N, 4.94; S, 5.65. Found: C, 61.14; H, 4.30; N, 5.00; S, 5.63%.

## Biology protocols

### Human carbonic anhydrases (I, II, IX, and XII) inhibitory assay

Carbonic anhydrase inhibition was evaluated using a stopped-flow CO_2_ hydration assay as previously described.^45–49^ Enzymatic activity was monitored by following the absorbance change of phenol red at 557 nm in HEPES buffer (pH 7.5). Initial reaction velocities were determined from multiple kinetic traces after correction for the non-enzymatic hydration of CO_2_. Inhibitor stock solutions were prepared in DMSO, serially diluted with assay buffer, and preincubated with the enzyme for 15 min prior to the measurements. Inhibition constants (*K*_I_) were determined by nonlinear regression analysis using the Cheng–Prusoff equation. All reported *K*_I_ values represent the mean of three independent experiments, with experimental errors within ±5–10% of the reported values.^45,50–53^

#### *In vitro* antiproliferative assay

The antiproliferative activities of the synthesized compounds were assessed against the human breast cancer cell lines MCF-7 and MDA-MB-231 using the MTT colorimetric assay, with the non-tumorigenic human breast epithelial cell line MCF-10A serving as the normal control.^54,55^ Cells were seeded into 96-well plates at a density of 2.1–2.3 × 10^4^ cells per well and cultured in RPMI-1640 medium supplemented with 10% fetal calf serum (FCS), bovine insulin, and antibiotics under a humidified atmosphere containing 5% CO_2_ at 37 °C. Test compounds were initially dissolved in DMSO and subsequently diluted to obtain eight serial concentrations ranging from 0.1 to 300 µM. Following 72 h of treatment, MTT solution (0.5 µg µL^−1^) was added, and the plates were incubated for an additional 4 h. The resulting formazan crystals were dissolved in a DMSO/isopropanol mixture (1 : 1, v/v), and absorbance was measured at 590 nm using a microplate reader. All experiments were performed in triplicate, and the results are expressed as the mean ± SD.

#### HSP90α (N-terminal) inhibition assay

The inhibitory activities of the selected compounds against the N-terminal ATP-binding domain of human HSP90α were determined using the HSP90α (N-Terminal) Geldanamycin Competitive Inhibitor Assay Kit (BPS Bioscience, San Diego, CA, USA; Cat. No. 50293) following the manufacturer's protocol. Briefly, recombinant human HSP90α was incubated with serial concentrations of the test compounds in the presence of a fluorescein (FITC)-labeled geldanamycin tracer. Fluorescence polarization was subsequently measured using a BioTek Synergy Hybrid Multi-Mode Microplate Reader (excitation: 475 nm; emission: 518 nm). The half-maximal inhibitory concentrations (IC_50_) were calculated by nonlinear regression analysis using GraphPad Prism version 8.0. All measurements were performed in five independent experiments, and the IC_50_ values are presented as the mean ± standard deviation (SD).

#### Quantitative reverse transcription PCR (qRT-PCR)

The effects of the lead compound 4b on the expression of apoptosis-related genes were investigated in MDA-MB-231 cells treated at its IC_50_ concentration. The mRNA expression levels of BAX, Bcl-2, p53, and caspase-9 were quantified by quantitative real-time PCR (qRT-PCR) and compared with those of the vehicle-treated control group (0.01% DMSO).^56,57^ Total RNA was isolated using the RNeasy Mini Kit (Qiagen, Germany), followed by reverse transcription into complementary DNA (cDNA) using the RevertAid First Strand cDNA Synthesis Kit (Thermo Scientific, USA). Quantitative PCR amplification was subsequently performed using SYBR® Green Master Mix (Bio-Rad, USA) on a Rotor-Gene Q Real-Time PCR System. GAPDH was used as the internal reference gene for normalization, and relative gene expression levels were calculated from five independent experiments performed in triplicate.

#### Molecular docking

The X-ray structures of the human CA IX complex (PDB ID: 5FL6) and HSP90α-onalespib complex (PDB ID: 2XJX) were downloaded from the Research Collaboratory for Structural Bioinformatics (RCSB) Protein Data Bank.^41,44,58^ Compound 4b was drawn using MarvinSketch 22.9.^59^ Regarding CA IX, the AutoDock4Zn protocol, which is implemented in AutoDock Vina 1.2, was used while keeping the catalytic Zn^2+^ ion in the active site.^60^ AutoDockTools 1.5.7 was used to define the grid box.^61^ The grid box dimensions were 48 × 44 × 36 points along the *x*-, *y*-, and *z*-axes, respectively, and the center coordinates *x* = −33, *y* = −1, and *z* = −2. Regarding HSP90α, the docking was performed by AutoDock Vina 1.2, utilizing the UCSF Chimera graphical user interface for preparation of both the ligand and the protein.^62–64^ The grid box dimensions were 10 × 12 × 21 points along the *x*-, *y*-, and *z*-axes, respectively, and grid center coordinates were *x* = 15, *y* = −3, and *z* = 2. For both targets, the exhaustiveness was set to 1000. The 2D and 3D interaction plots were generated using Discovery Studio (DS) visualizer.^65^

#### Molecular dynamics simulation protocol

The docked complexes of compound 4b with CA IX and HSP90α were prepared for dynamic simulation using the web-based graphical user interface for CHARMM (CHARMM-GUI).^66–70^ The molecular dynamics simulations for both docked complexes were performed with GROMACS 2026, using a cubic box encompassing the TIP3P water model, Na^+^ and Cl^−^ ions.^71^ The CHARMM General Force Field (CGenFF) was utilized for compound 4b parametrization, while the CHARMM36 forcefield was used for the parametrization of CA IX and HSP90α.^69,72^ The energy minimization algorithm was steepest descent using 50 000 steps. The molecular dynamics simulation for 100 ns was run with an integration step of 2 fs, following NVT equilibration for 125 ps and NPT equilibration for 500 ps. The temperature was adjusted to 310 K, and the pressure to 1 bar. Temperature and pressure coupling was achieved using the stochastic cell-rescaling (c-rescale) barostat and the velocity-rescale (v-rescale) thermostat, respectively. The LINCS method was used to constrain the hydrogen atoms, while the Verlet cutoff was used to generate the neighbor list. Long-range electrostatic interactions were computed using the particle-mesh Ewald (PME) method. The coulombic and van der Waals interaction cutoffs were set to 12 Å. The data frames were saved every 200 ps. Trajectory analyses for both CA IX and HSP90α were performed using the GROMACS tools.^73^ MM/PBSA (Molecular Mechanics/Poisson–Boltzmann Surface Area) computations were done *via* the gmx_MMPBSA tool version (v1.6.4).^74^

## Conflicts of interest

There are no conflicts to declare.

## Supplementary Material

RA-016-D6RA06511B-s001

## Data Availability

All data generated or analyzed during this study are included in this manuscript and its supplementary information (SI) file. Supplementary information is available. See DOI: https://doi.org/10.1039/d6ra06511b.
