# Supplementary material for: Harnessing coumarin privilege to achieve potency and selectivity: hypoxia-targeted alkyl coumarin-tethered phenyl quinazolinones as dual hCA IX/HSP90α inhibitors with chemosensitizing activity
Source: RSC Adv. 2026 Sep 9;16(43):53613–39. doi: 10.1039/d6ra06511b (PMC13555529; doi:10.1039/d6ra06511b)
Supplement: Supplementary file 1 [file RA-016-D6RA06511B-s001.pdf]

Supplementary Information (SI) for RSC Advances.
This journal is © The Royal Society of Chemistry 2026

Supporting File

Harnessing Coumarin Privilege to Achieve Potency and Selectivity: Hypoxia-Targeted Alkyl Coumarin-Tethered Phenyl quinazolinones as Dual hCA IX/HSP90α Inhibitors with Chemosensitizing Activity

Amal S. Elgohary [1], Mervat H. El-Hamamsy [1], Nabaweya Sharafeldin [1], Simone Giovannuzzi [2], Triantafyllos-Dimitrios Gerokonstantis [3], Nikolaos S. Thomaidis [3], Claudiu T. Supuran [2,*], Haytham O. Tawfik [1,4,*]

[1] *Department of Pharmaceutical Chemistry, Faculty of Pharmacy, Tanta University, Tanta, 31527, Egypt*

[2] *Department of NEUROFARBA, Section of Pharmaceutical and Nutraceutical Sciences, University of Florence, Polo Scientifico, Via U. Schiff 6, 50019, Sesto Fiorentino, Firenze, Italy*

[3] *Laboratory of Analytical Chemistry, Department of Chemistry, National and Kapodistrian University of Athens, Panepistimiopolis Zografou, 15771 Athens, Greece*

[4] *Department of Pharmaceutical Chemistry, Faculty of Pharmacy, Alsalam University, Tanta, 31611, Egypt*

*Corresponding author: C. T. Supuran; claudiu.supuran@unifi.it, H. O. Tawfik; haytham.omar.mahmoud@pharm.tanta.edu.eg.

Table of Contents

Title	Page
**Yields and elemental analysis**	**S4**
**Table S1.** Yields and elemental analysis of intermediate compounds (**1a-d**, **2a-c**, and **3a-c**) and target compounds (**4a-l**)	**S4**
**Figure S1.** Elemental analysis of intermediate compounds (**1a-d**, **2a-c**, and **3a-c**)	**S5**
**Figure S2.** Elemental analysis of target compounds (**4a-l**)	**S5**
**Proposed mechanism**	**S5**
**Scheme S1.** Proposed mechanism for synthesis of 7-(3-chloropropoxy)-4-methyl-2*H*-chromen-2-one **(3a).**	**S5**
**^{1}H NMR and ^{13}C NMR-DEPTQ spectral data of the intermediates (2a, 3b, 3c) and the target compounds (4a-l)**	**S6**
**Figure S3.** ^{1}H NMR (500 MHz, DMSO-d_6) spectrum of compound **2a**	**S6**
**Figure S4.** ^{1}H NMR (500 MHz, DMSO-d_6) spectrum of compound **3b**	**S6**
**Figure S5.** ^{1}H NMR (500 MHz, DMSO-d_6) spectrum of compound **3c**	**S7**
**Figure S6.** ^{1}H NMR (500 MHz, DMSO-d_6) spectrum of compound **4a**	**S8**
**Figure S7.** ^{13}C NMR-DEPTQ (125 MHz, DMSO-d_6) spectrum of compound **4a**	**S8**
**Figure S8.** ^{1}H NMR (500 MHz, DMSO-d_6) spectrum of compound **4b**	**S9**
**Figure S9.** ^{13}C NMR-DEPTQ (125 MHz, DMSO-d_6) spectrum of compound **4b**	**S9**
**Figure S10.** HMQC spectrum of compound **4b** recorded in DMSO-d_6 (500/125 MHz)	**S10**
**Figure S11.** ^{1}H NMR (500 MHz, DMSO-d_6) spectrum of compound **4c**	**S11**
**Figure S12.** ^{13}C NMR-DEPTQ (125 MHz, DMSO-d_6) spectrum of compound **4c**	**S11**
**Figure S13.** ^{1}H NMR (500 MHz, DMSO-d_6) spectrum of compound **4d**	**S12**
**Figure S14.** ^{13}C NMR-DEPTQ (125 MHz, DMSO-d_6) spectrum of compound **4d**	**S12**
**Figure S15.** ^{1}H NMR (500 MHz, DMSO-d_6) spectrum of compound **4e**	**S13**
**Figure S16.** ^{13}C NMR-DEPTQ (125 MHz, DMSO-d_6) spectrum of compound **4e**	**S13**
**Figure S17.** ^{1}H NMR (500 MHz, DMSO-d_6) spectrum of compound **4f**	**S14**
**Figure S18.** ^{13}C NMR-DEPTQ (125 MHz, DMSO-d_6) spectrum of compound **4f**	**S14**
**Figure S19.** ^{1}H NMR (500 MHz, DMSO-d_6) spectrum of compound **4g**	**S15**
**Figure S20.** ^{13}C NMR-DEPTQ (125 MHz, DMSO-d_6) spectrum of compound **4g**	**S15**
**Figure S21.** ^{1}H NMR (500 MHz, DMSO-d_6) spectrum of compound **4h**	**S16**
**Figure S22.** ^{13}C NMR-DEPTQ (125 MHz, DMSO-d_6) spectrum of compound **4h**	**S16**
**Figure S23.** ^{1}H NMR (500 MHz, DMSO-d_6) spectrum of compound **4i**	**S17**
**Figure S24.** ^{13}C NMR-DEPTQ (125 MHz, DMSO-d_6) spectrum of compound **4i**	**S17**
**Figure S25.** ^{1}H NMR (500 MHz, DMSO-d_6) spectrum of compound **4j**	**S18**
**Figure S26.** ^{13}C NMR-DEPTQ (125 MHz, DMSO-d_6) spectrum of compound **4j**	**S18**

**Figure S27.** ^{1}H NMR (500 MHz, DMSO-d_6) spectrum of compound **4k**	**S19**
**Figure S28.** ^{13}C NMR-DEPTQ (125 MHz, DMSO-d_6) spectrum of compound **4k**	**S19**
**Figure S29.** ^{1}H NMR (500 MHz, DMSO-d_6) spectrum of compound **4l**	**S20**
**Figure S30.** ^{13}C NMR-DEPTQ (125 MHz, DMSO-d_6) spectrum of compound **4l**	**S20**
**Mass spectral data of the target compounds (4a-l)**	**S21**
**Figure S31. (A)** Mass spectrum of compound **4a**, and **(B)** Zoom in spectra for range 460-540.	**S21**
**Figure S32. (A)** Mass spectrum of compound **4b**, and **(B)** Zoom in spectra for range 480-580.	**S22**
**Figure S33. (A)** Mass spectrum of compound **4c**, and **(B)** Zoom in spectra for range 500-540.	**S23**
**Figure S34. (A)** Mass spectrum of compound **4d**, **(B)** Zoom in spectra for range 510-590, and **(C)** Isotopic profile of chloride.	**S24**
**Figure S35. (A)** Mass spectrum of compound **4e**, **(B)** Zoom in spectra for range 510-590, and **(C)** Isotopic profile of chloride.	**S25**
**Figure S36. (A)** Mass spectrum of compound **4f**, **(B)** Zoom in spectra for range 500-600, and **(C)** Isotopic profile of chloride.	**S26**
**Figure S37. (A)** Mass spectrum of compound **4g**, **(B)** Zoom in spectra for range 460-580, and **(C)** Isotopic profile of chloride.	**S27**
**Figure S38. (A)** Mass spectrum of compound **4h**, **(B)** Zoom in spectra for range 520-590, and **(C)** Isotopic profile of chloride.	**S28**
**Figure S39. (A)** Mass spectrum of compound **4i**, **(B)** Zoom in spectra for range 500-600, and **(C)** Isotopic profile of chloride.	**S29**
**Figure S40. (A)** Mass spectrum of compound **4j**, **(B)** Zoom in spectra for range 500-660, and **(C)** Isotopic profile of chloride.	**S30**
**Figure S41. (A)** Mass spectrum of compound **4k**, **(B)** Zoom in spectra for range 550-630, and **(C)** Isotopic profile of chloride.	**S31**
**Figure S42. (A)** Mass spectrum of compound **4l**, **(B)** Zoom in spectra for range 565-610, and **(C)** Isotopic profile of chloride.	**S32**
**HPLC purity**	**S33**
**Table S2.** HPLC purity of target compounds **(4a-l)**	**S33**
**Figure S43.** HPLC chromatogram of compound **4a**	**S34**
**Figure S44.** HPLC chromatogram of compound **4b**	**S35**
**Figure S45.** HPLC chromatogram of compound **4c**	**S36**
**Figure S46.** HPLC chromatogram of compound **4d**	**S37**
**Figure S47.** HPLC chromatogram of compound **4e**	**S38**
**Figure S48.** HPLC chromatogram of compound **4f**	**S39**
**Figure S49.** HPLC chromatogram of compound **4g**	**S40**
**Figure S50.** HPLC chromatogram of compound **4h**	**S41**
**Figure S51.** HPLC chromatogram of compound **4i**	**S42**
**Figure S52.** HPLC chromatogram of compound **4j**	**S43**
**Figure S53.** HPLC chromatogram of compound **4k**	**S44**
**Figure S54.** HPLC chromatogram of compound **4l**	**S45**

Yields and elemental analysis

Table S1. Yields and elemental analysis of intermediate compounds (**1a-d**, **2a-c**, and **3a-c**) and target compounds (**4a-l**)

Code	Yield (%)	Elemental analysis							
		Calculated				Found			
		C	H	N	S	C	H	N	S
**1a**	81	66.12	3.96	11.02	12.61	66.25	3.97	10.94	12.55
**1b**	55	58.24	3.14	9.70	11.10	58.01	3.11	9.70	11.01
**1c**	87	58.24	3.14	9.70	11.10	58.49	3.15	9.56	11.14
**1d**	66	52.03	2.50	8.67	9.92	51.89	2.49	8.59	9.99
**2a**	64	68.18	4.58	-	-	68.34	4.61	-	-
**2b**	75	70.58	5.92	-	-	70.77	5.85	-	-
**2c**	66	70.58	5.92	-	-	70.75	5.89	-	-
**3a**	72	61.79	5.19	-	-	61.49	5.23	-	-
**3b**	85	64.17	6.10	-	-	63.94	6.14	-	-
**3c**	72	64.17	6.10	-	-	64.43	6.09	-	-
**4a**	68	68.92	4.71	5.95	6.81	69.11	4.69	5.90	6.80
**4b**	50	69.86	5.26	5.62	6.43	69.54	5.25	5.66	6.39
**4c**	55	69.86	5.26	5.62	6.43	70.04	5.19	5.64	6.45
**4d**	80	64.22	4.19	5.55	6.35	63.99	4.11	5.49	6.33
**4e**	85	65.35	4.73	5.26	6.01	65.09	4.76	5.22	5.97
**4f**	70	65.35	4.73	5.26	6.01	65.04	4.69	5.29	6.04
**4g**	62	64.22	4.19	5.55	6.35	63.96	4.24	5.58	6.36
**4h**	80	65.35	4.73	5.26	6.01	65.65	4.65	5.31	6.07
**4i**	65	65.35	4.73	5.26	6.01	65.51	4.77	5.21	6.00
**4j**	61	60.12	3.74	5.19	5.94	59.90	3.80	5.22	5.99
**4k**	73	61.38	4.26	4.94	5.65	61.69	4.31	4.96	5.69
**4l**	62	61.38	4.26	4.94	5.65	61.14	4.30	5.00	5.63

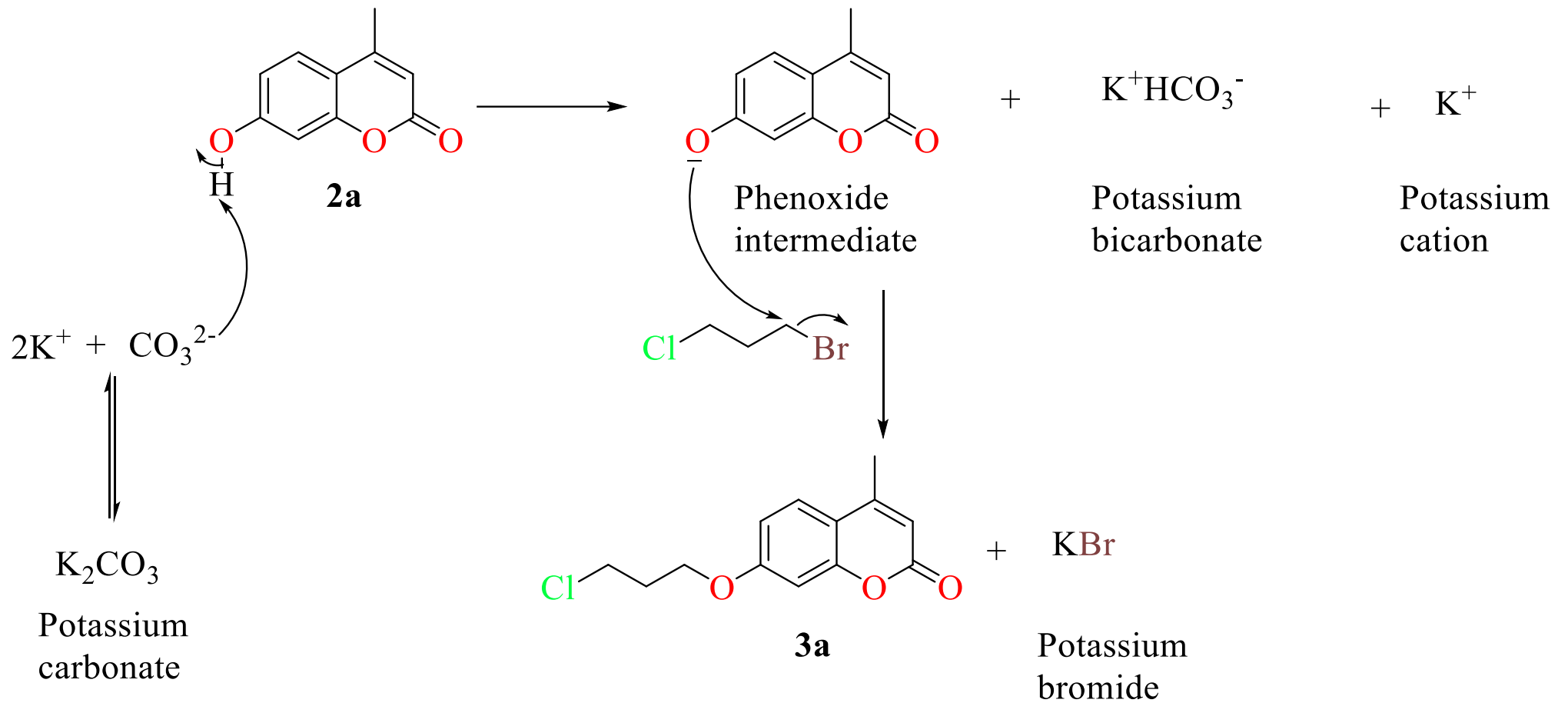

Scheme S1. Proposed mechanism for synthesis of 7-(3-chloropropoxy)-4-methyl-2*H*-chromen-2-one (**3a**).

Analysis Report

No.	Code	C%	H%	N%	S%
1	**1a**	66.25	3.97	10.94	12.55
2	**1b**	58.01	3.11	9.70	11.01
3	**1c**	58.49	3.15	9.56	11.14
4	**1d**	51.89	2.49	8.59	9.99
5	**2a**	68.34	4.61	-	-
6	**2b**	70.77	5.85	-	-
7	**2c**	70.75	5.89	-	-
8	**3a**	61.49	5.23	-	-
9	**3b**	63.94	6.14	-	-
10	**3c**	64.43	6.09	-	-

Figure S1. Elemental analysis of intermediate compounds (**1a-d**, **2a-c**, and **3a-c**)

Analysis Report

No.	Code	C%	H%	N%	S%
1	**4a**	69.11	4.69	5.90	6.80
2	**4b**	69.54	5.25	5.66	6.39
3	**4c**	70.04	5.19	5.64	6.45
4	**4d**	63.99	4.11	5.49	6.33
5	**4e**	65.09	4.76	5.22	5.97
6	**4f**	65.04	4.69	5.29	6.04
7	**4g**	63.96	4.24	5.58	6.36
8	**4h**	65.65	4.65	5.31	6.07
9	**4i**	65.51	4.77	5.21	6.00
10	**4j**	59.90	3.80	5.22	5.99
11	**4k**	61.69	4.31	4.96	5.69
12	**4l**	61.14	4.30	5.00	5.63

Figure S2. Elemental analysis of target compounds (**4a-l**)

^{1}H NMR and ^{13}C NMR-DEPTQ spectral data of the intermediates (2a, 3b, 3c) and the target compounds (4a-l)

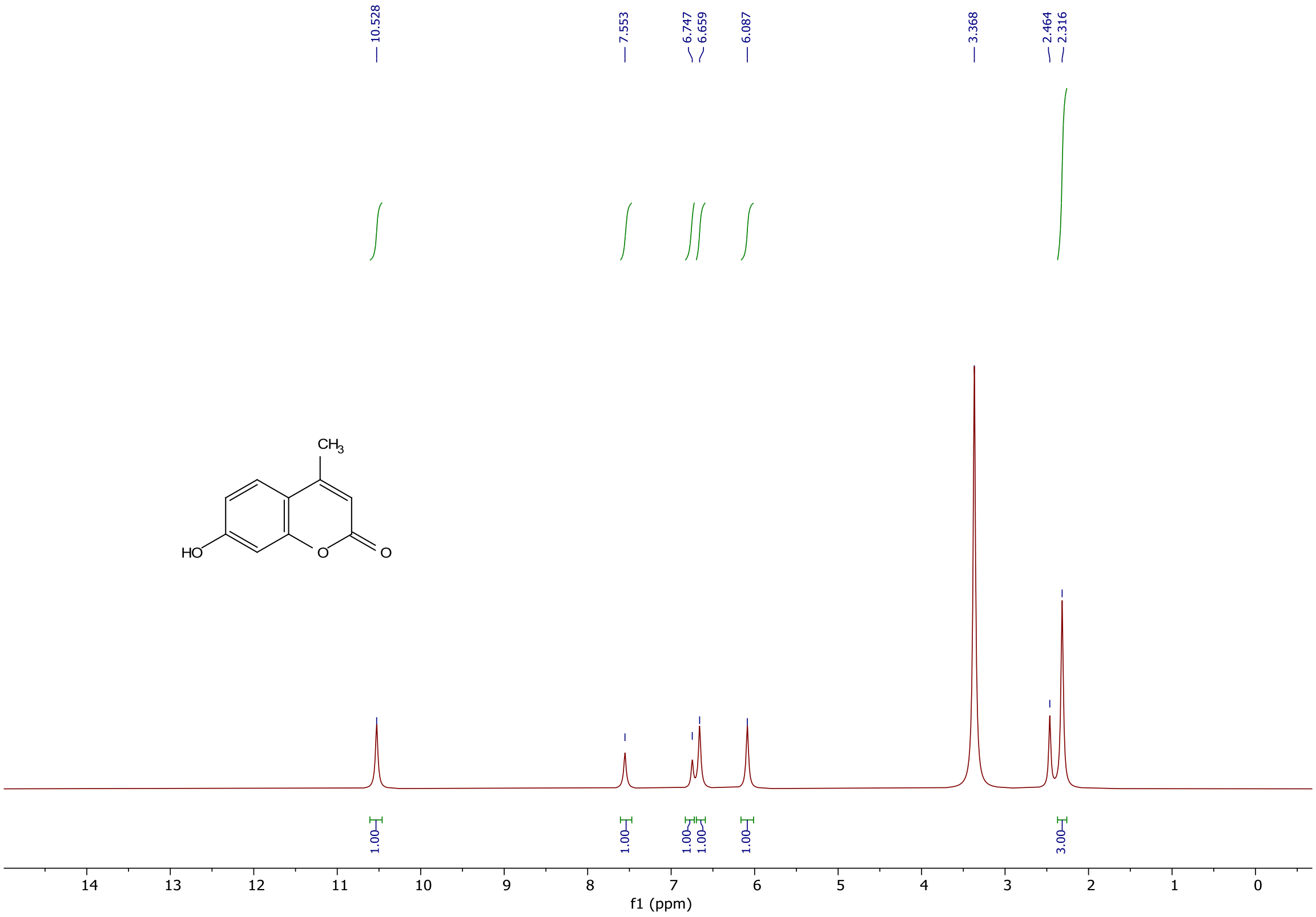

Figure S3. ^{1}H NMR (500 MHz, DMSO-d_6) spectrum of compound **2a**

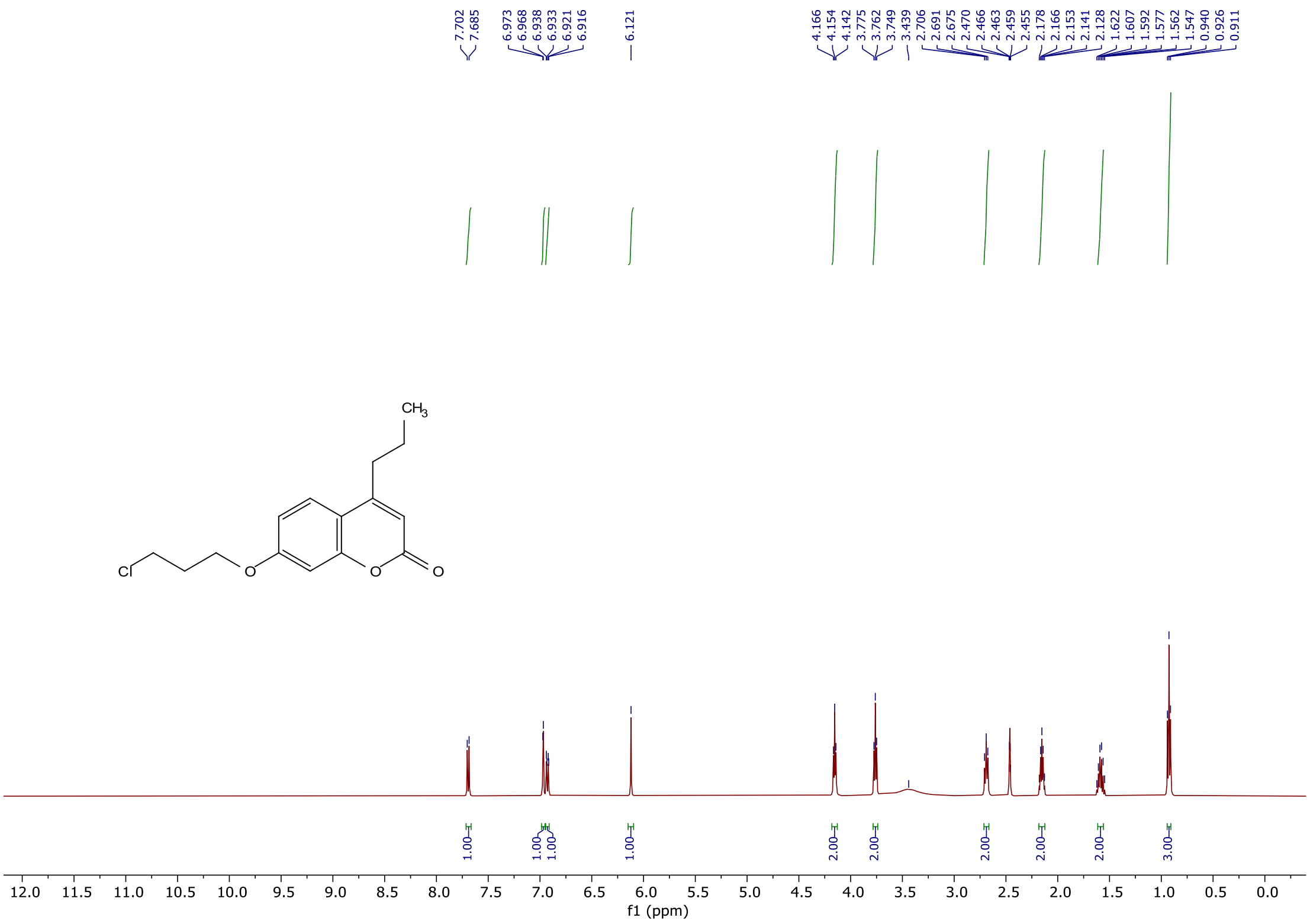

Figure S4. ^{1}H NMR (500 MHz, DMSO-d_6) spectrum of compound **3b**

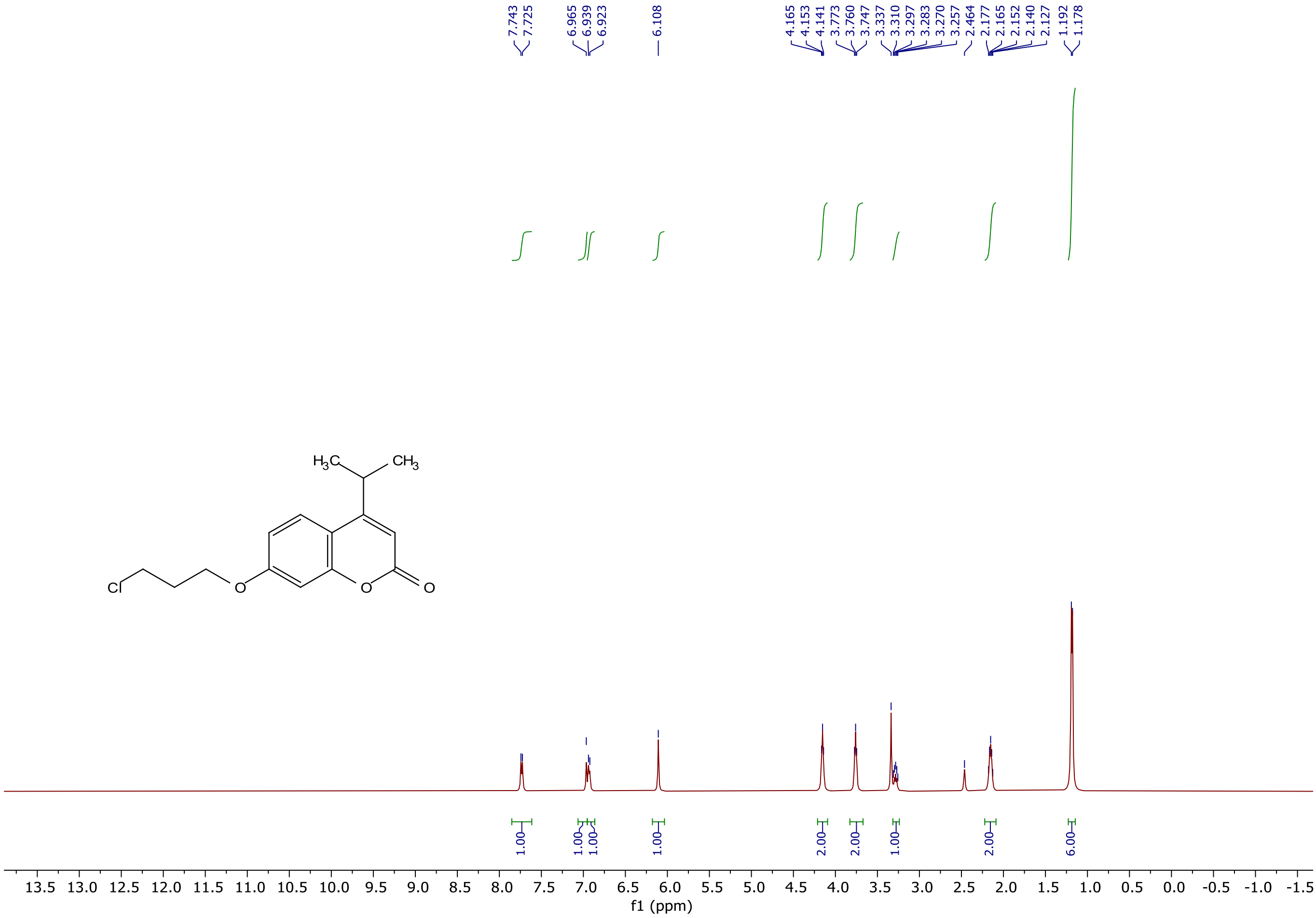

Figure S5. ^{1}H NMR (500 MHz, DMSO-d_6) spectrum of compound **3c**

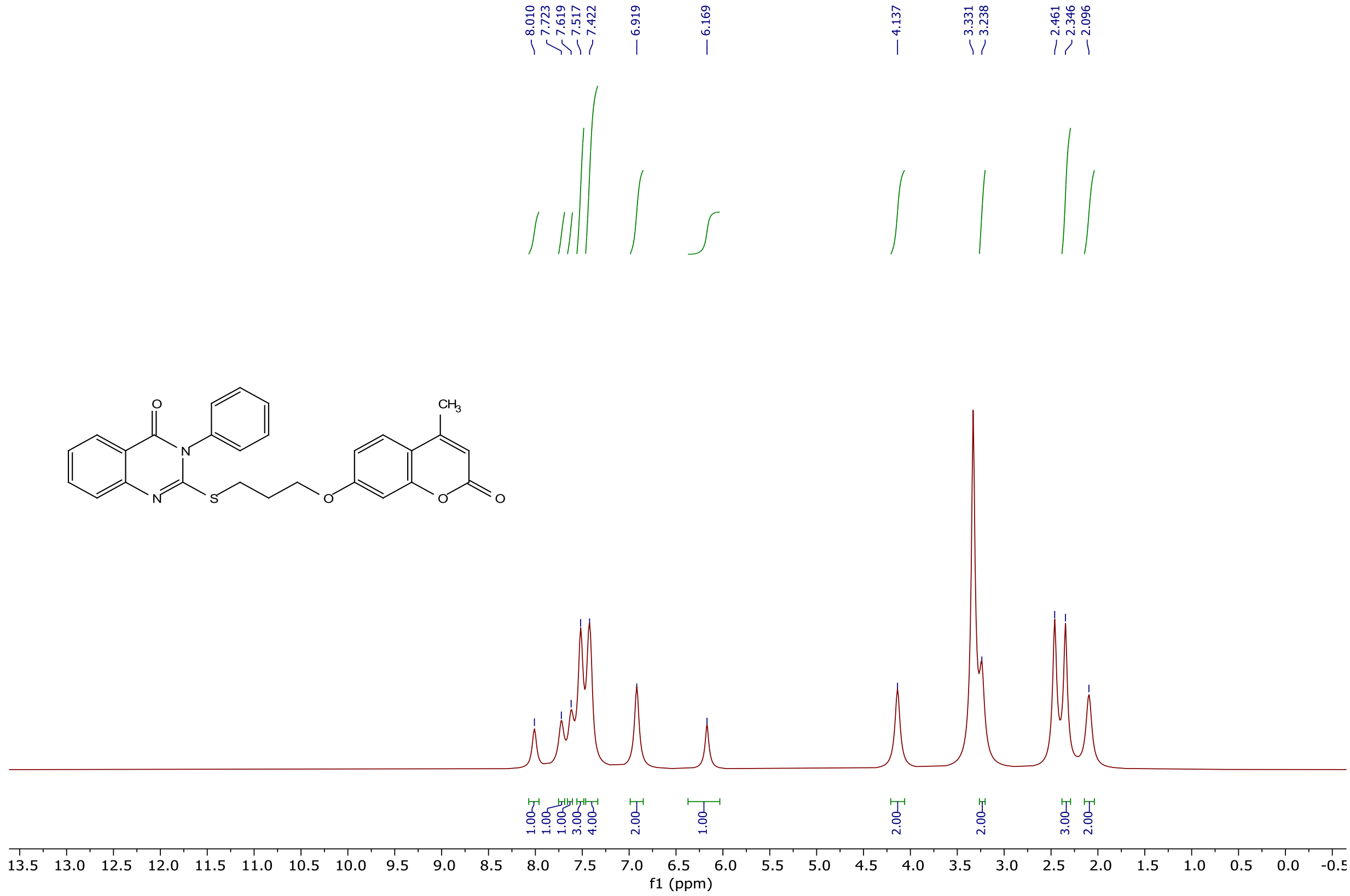

Figure S6. ^{1}H NMR (500 MHz, DMSO-d_6) spectrum of compound **4a**

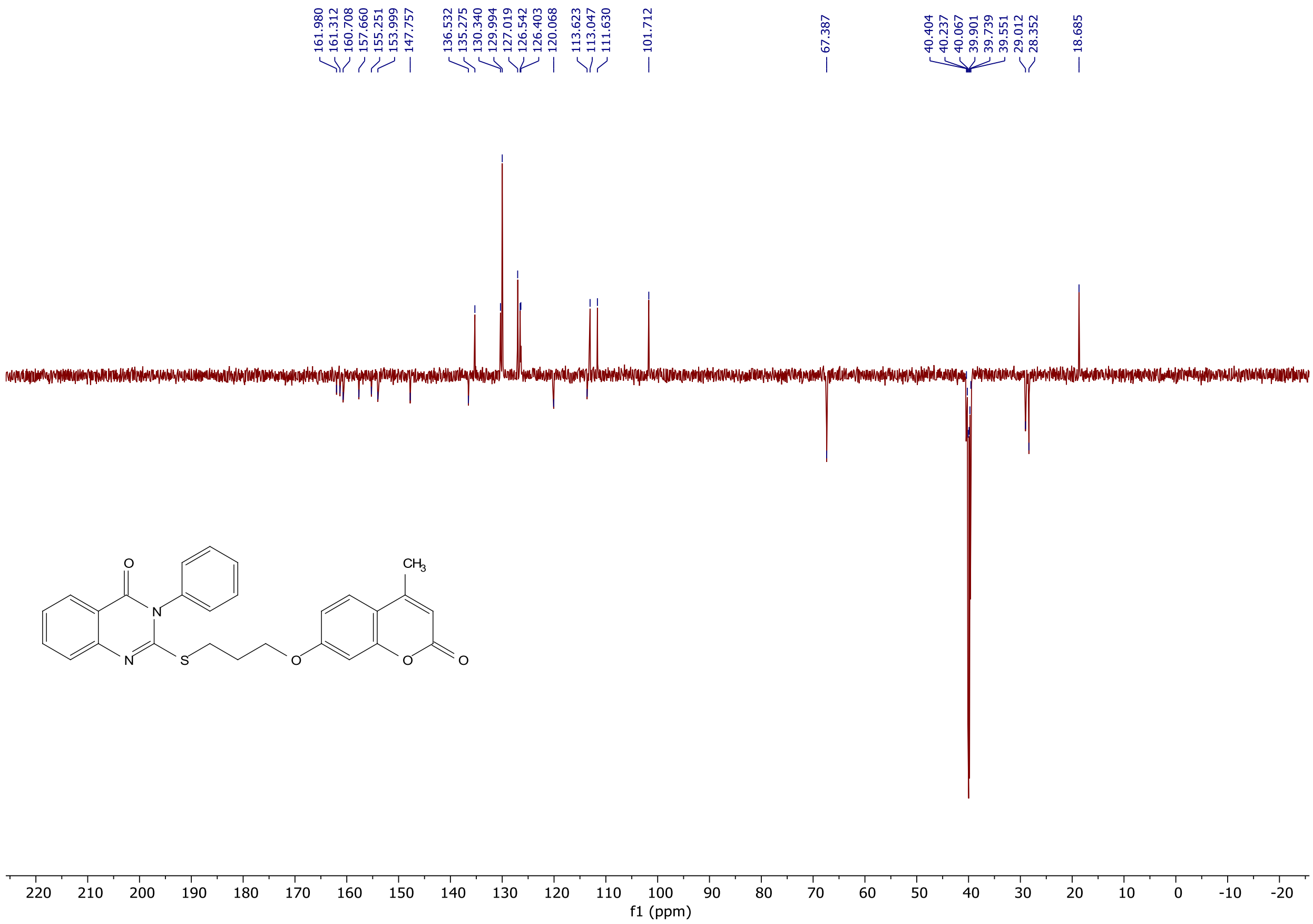

Figure S7. ^{13}C NMR-DEPTQ (125 MHz, DMSO-d_6) spectrum of compound **4a**

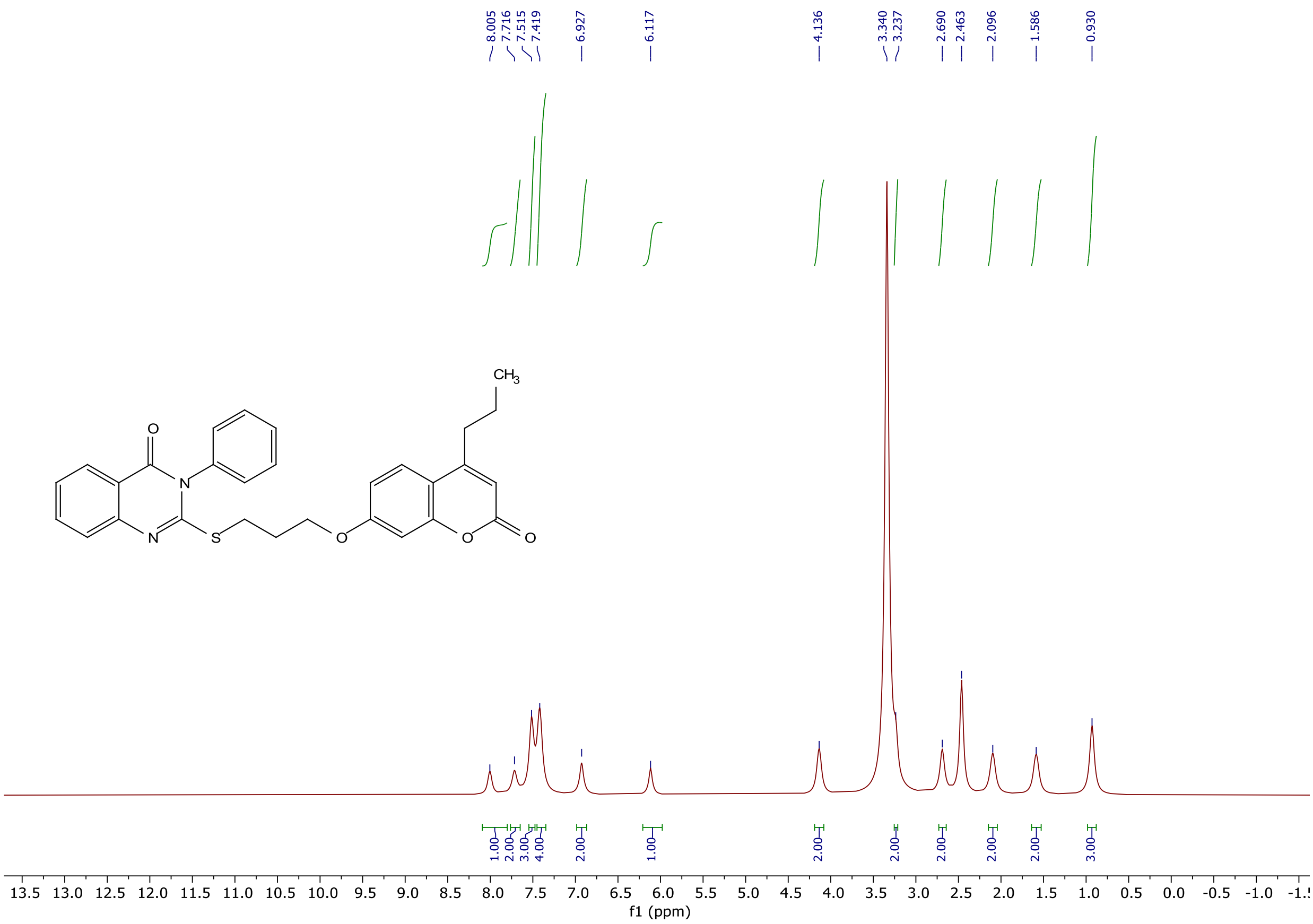

Figure S8. ^{1}H NMR (500 MHz, DMSO-d_6) spectrum of compound **4b**

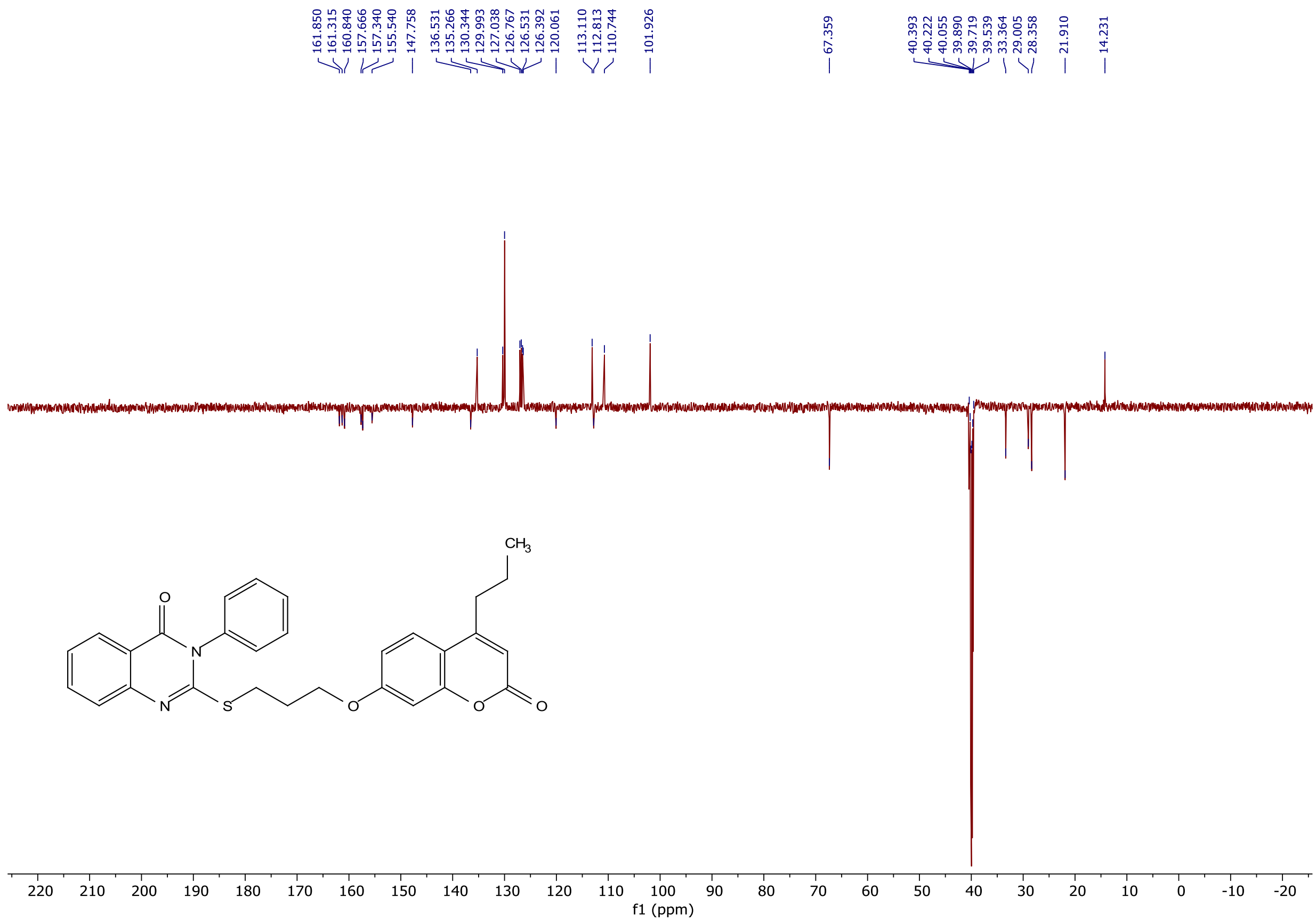

Figure S9. ^{13}C NMR-DEPTQ (125 MHz, DMSO-d_6) spectrum of compound **4b**

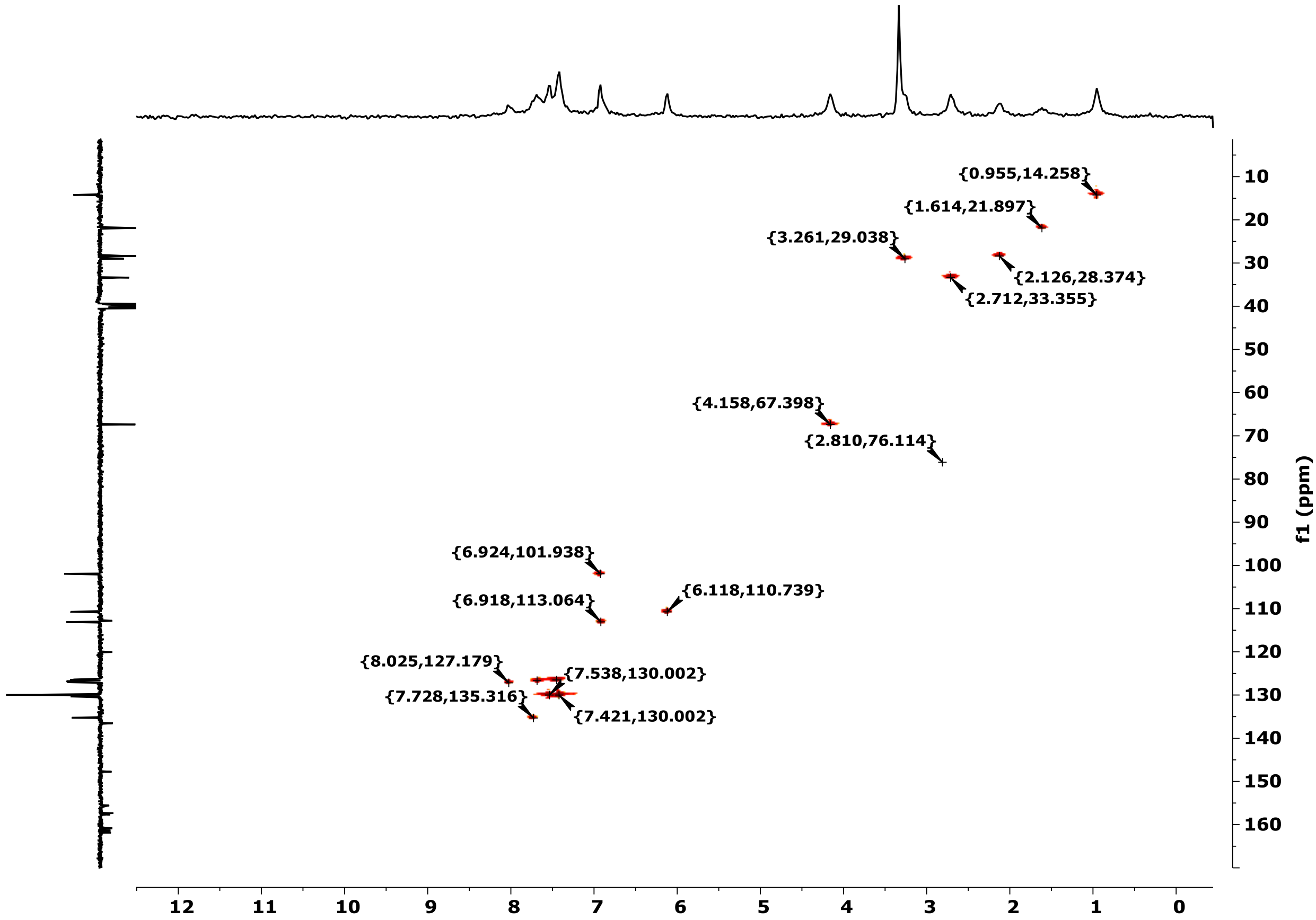

Figure S10. HMQC spectrum of compound **4b** recorded in DMSO-d_6 (500/125 MHz)

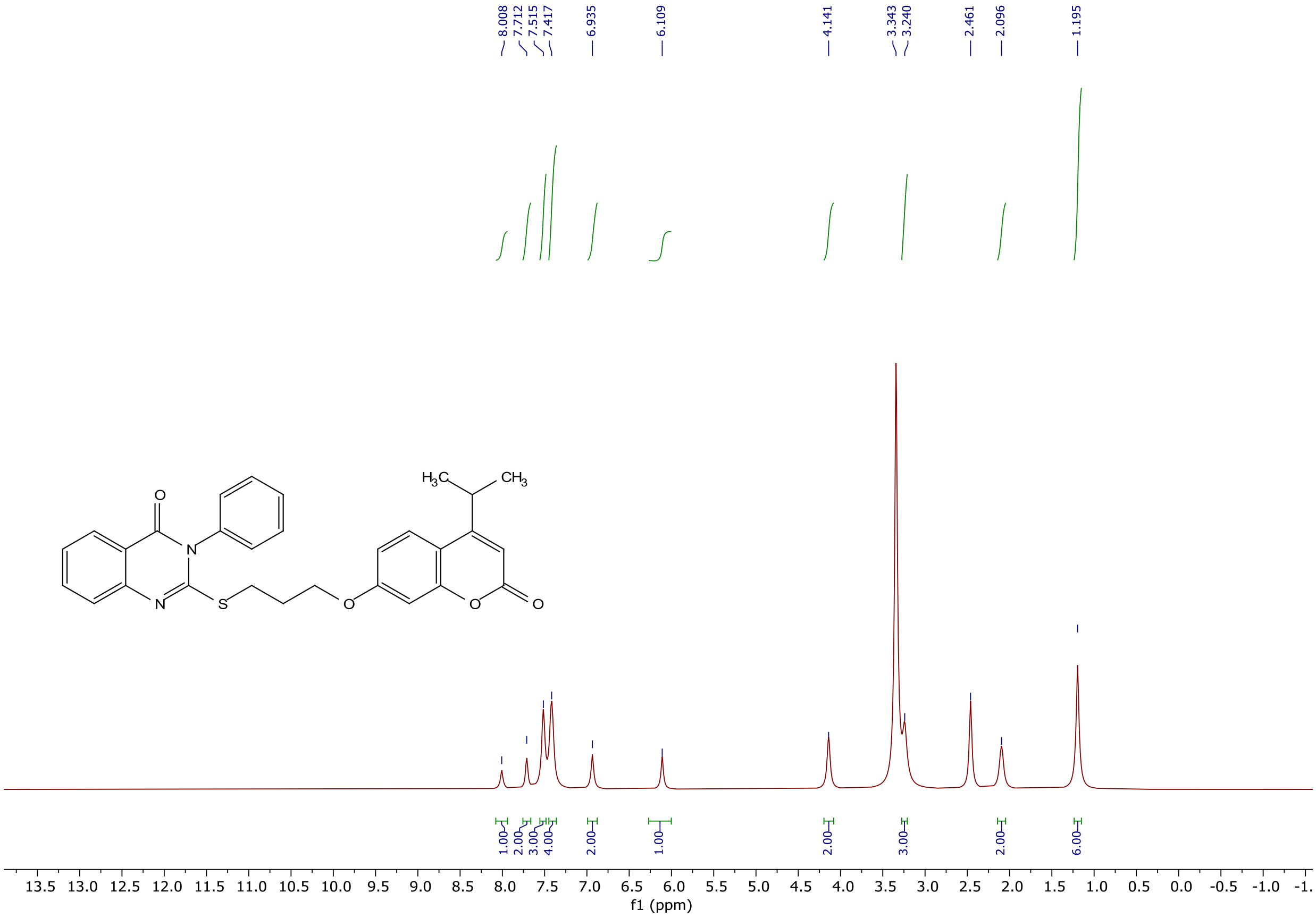

Figure S11. ^{1}H NMR (500 MHz, DMSO-d_6) spectrum of compound **4c**

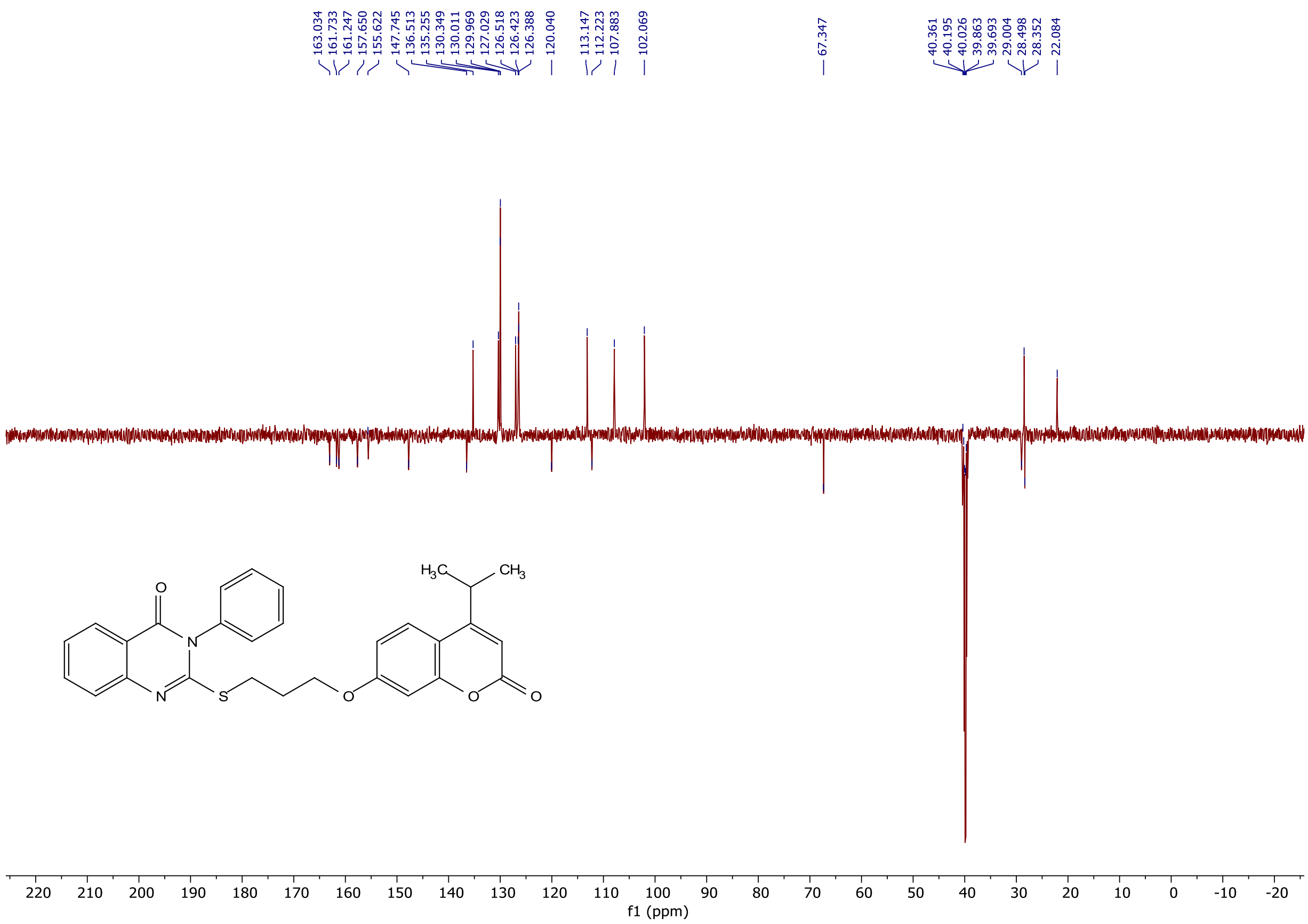

Figure S12. ^{13}C NMR-DEPTQ (125 MHz, DMSO-d_6) spectrum of compound **4c**

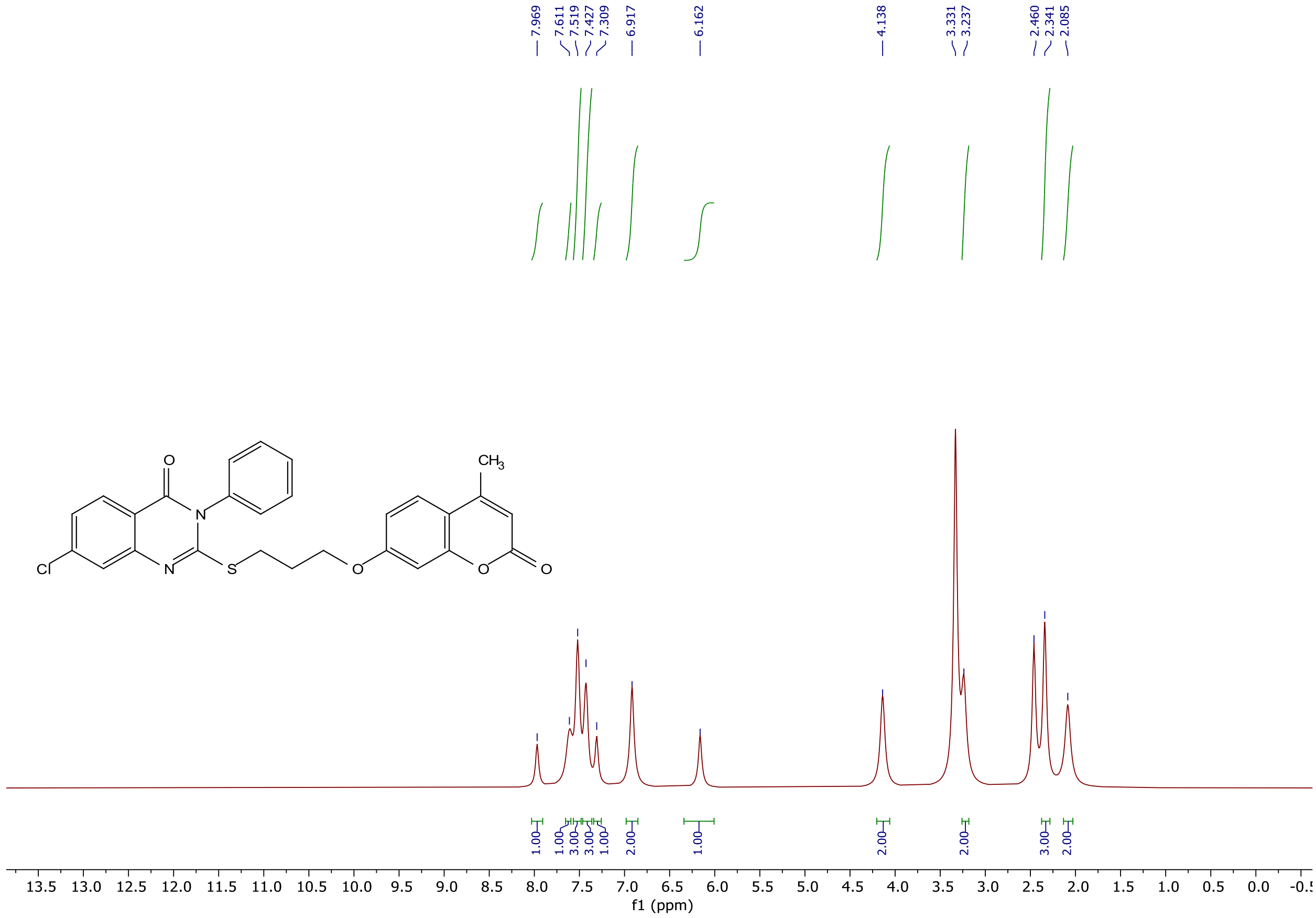

Figure S13. ^{1}H NMR (500 MHz, DMSO-d_6) spectrum of compound **4d**

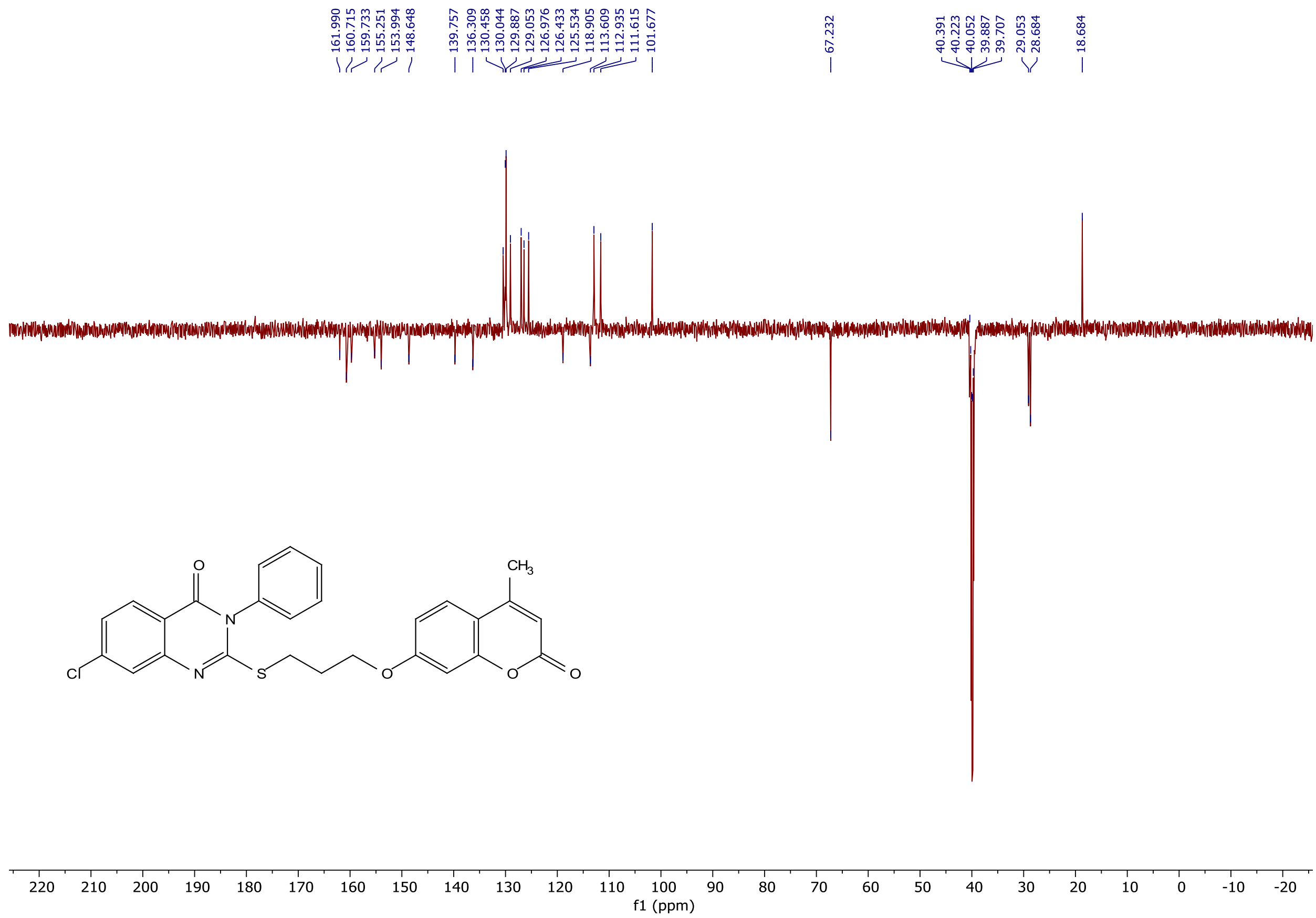

Figure S14. ^{13}C NMR-DEPTQ (125 MHz, DMSO-d_6) spectrum of compound **4d**

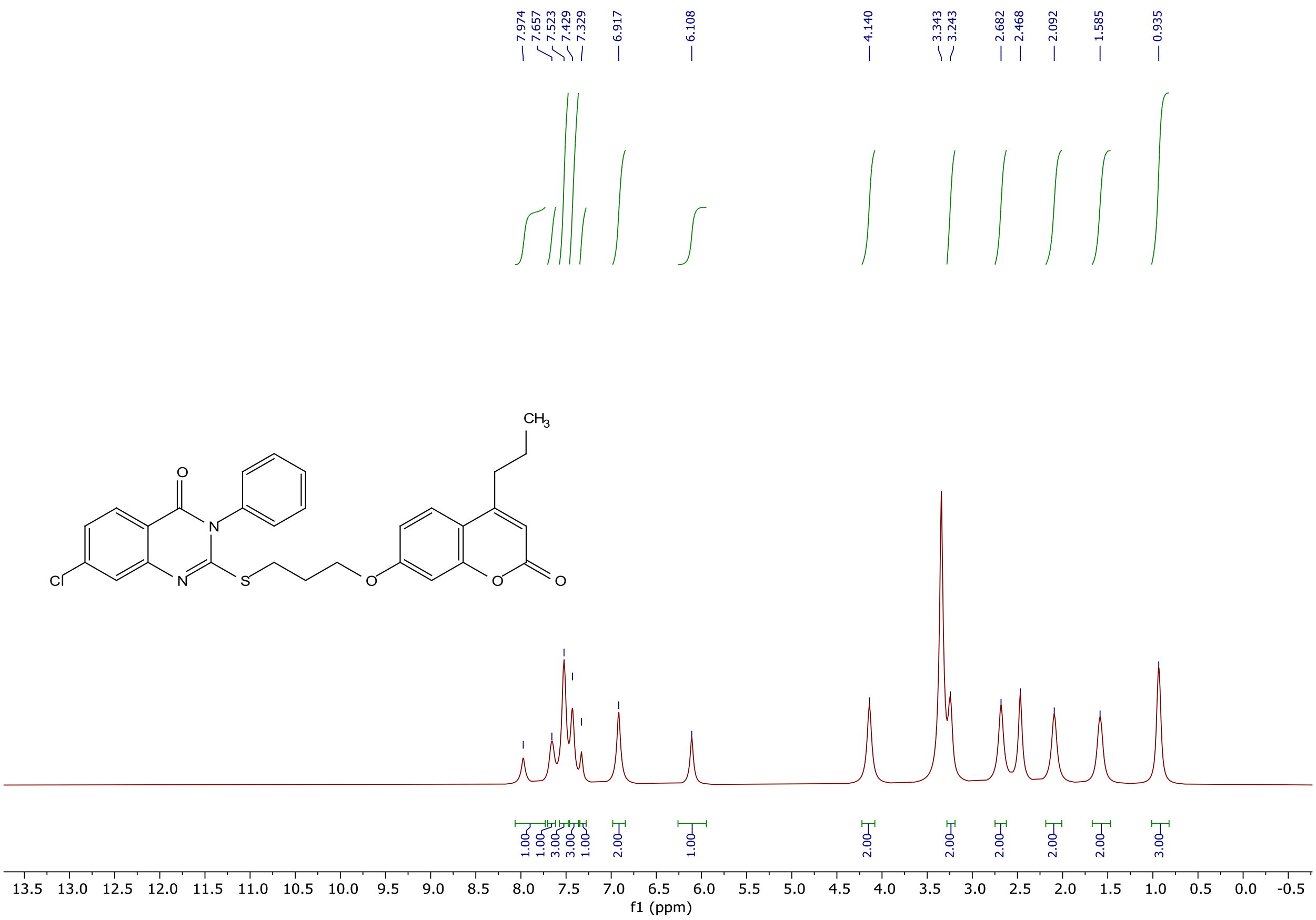

Figure S15. ^{1}H NMR (500 MHz, DMSO-d_6) spectrum of compound **4e**

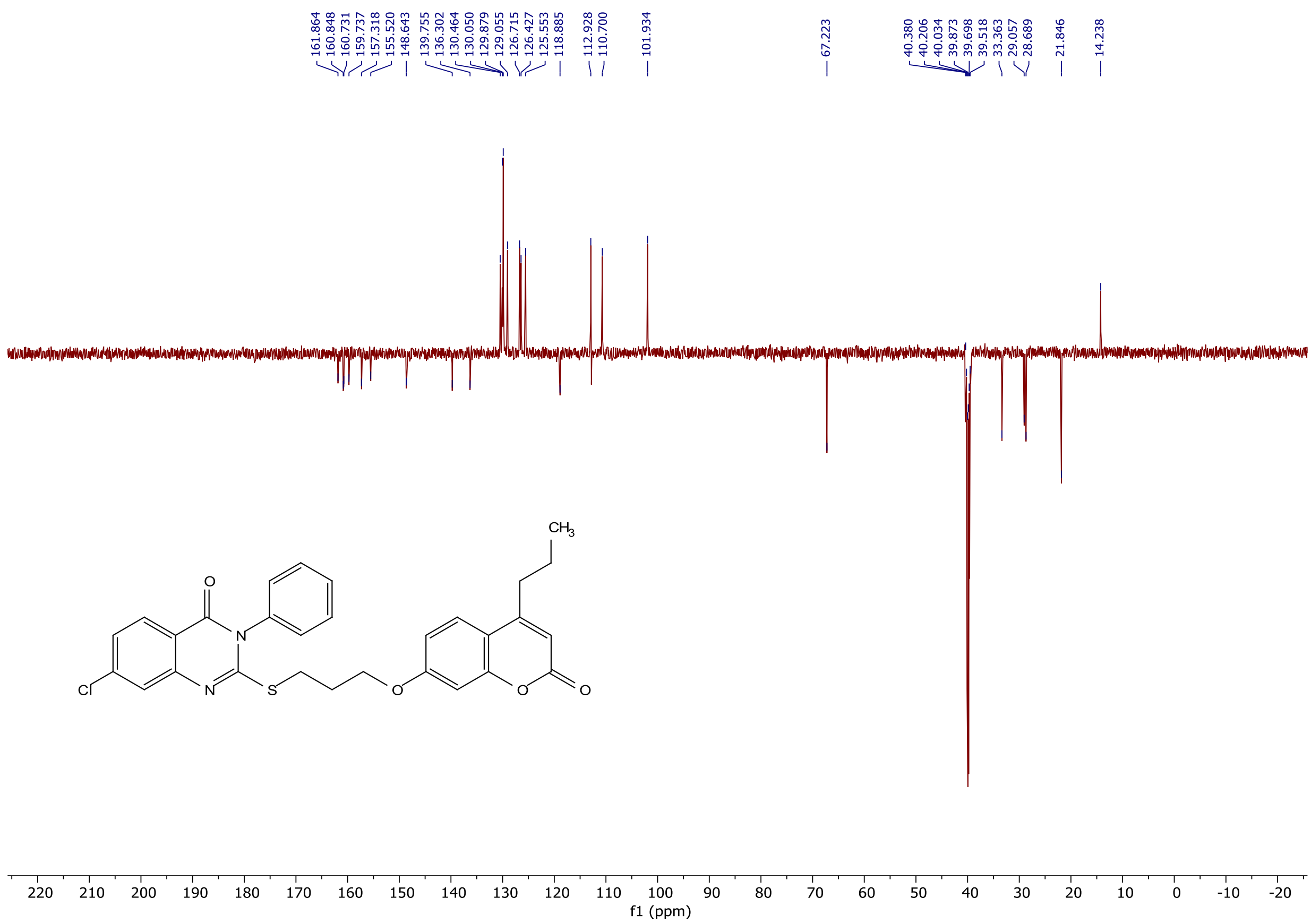

Figure S16. ^{13}C NMR-DEPTQ (125 MHz, DMSO-d_6) spectrum of compound **4e**

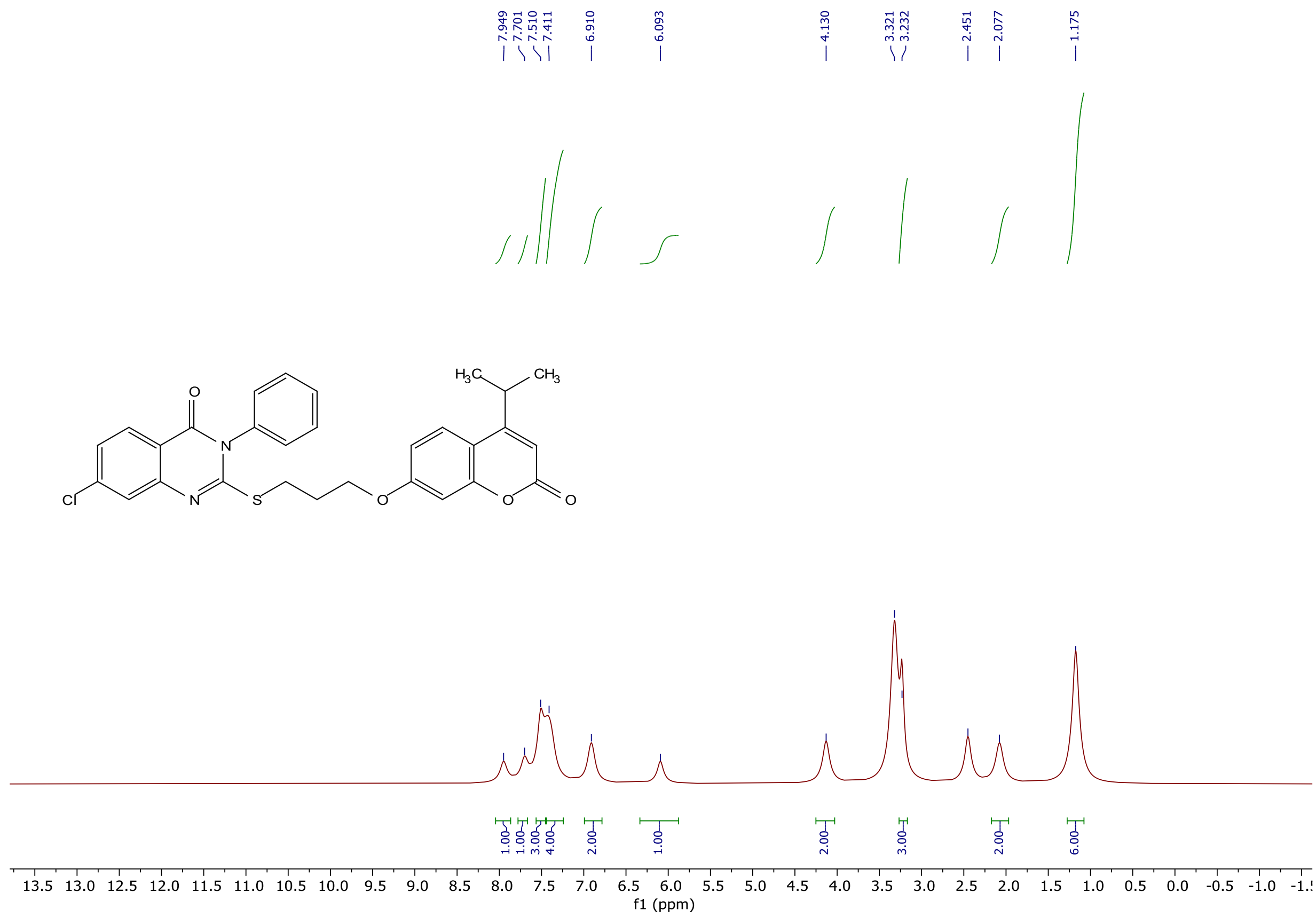

Figure S17. ^{1}H NMR (500 MHz, DMSO-d_6) spectrum of compound **4f**

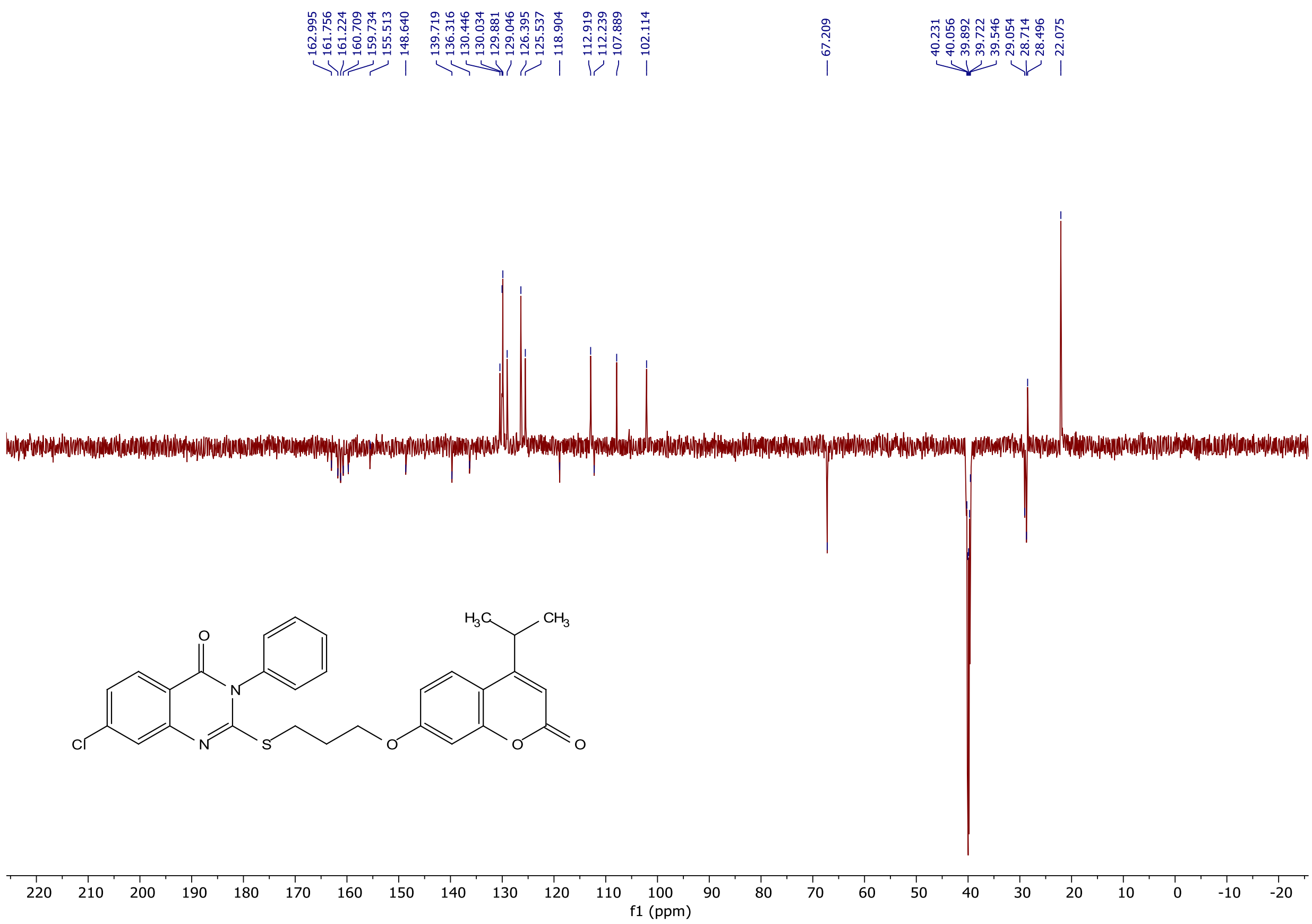

Figure S18. ^{13}C NMR-DEPTQ (125 MHz, DMSO-d_6) spectrum of compound **4f**

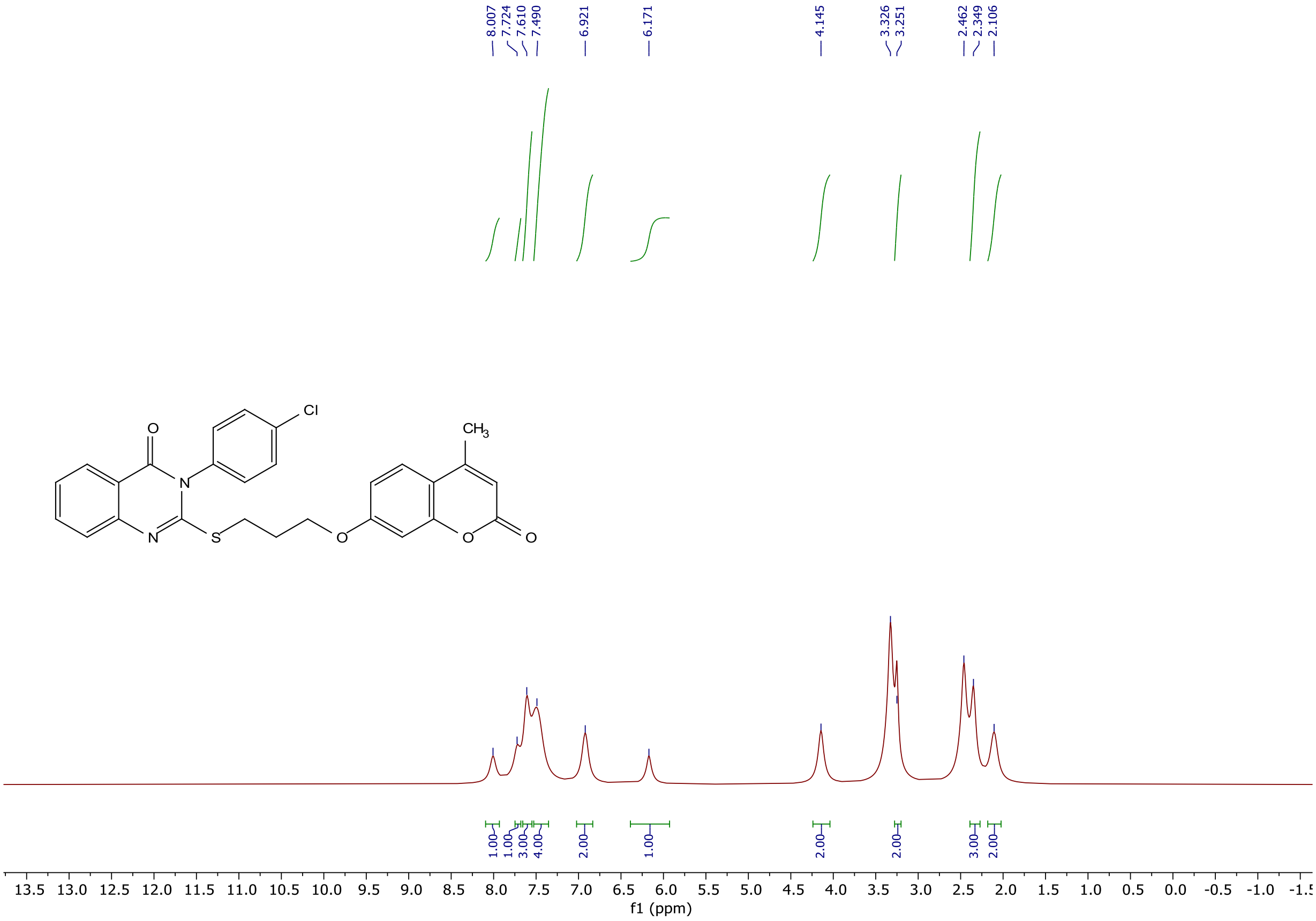

Figure S19. ^{1}H NMR (500 MHz, DMSO-d_6) spectrum of compound **4g**

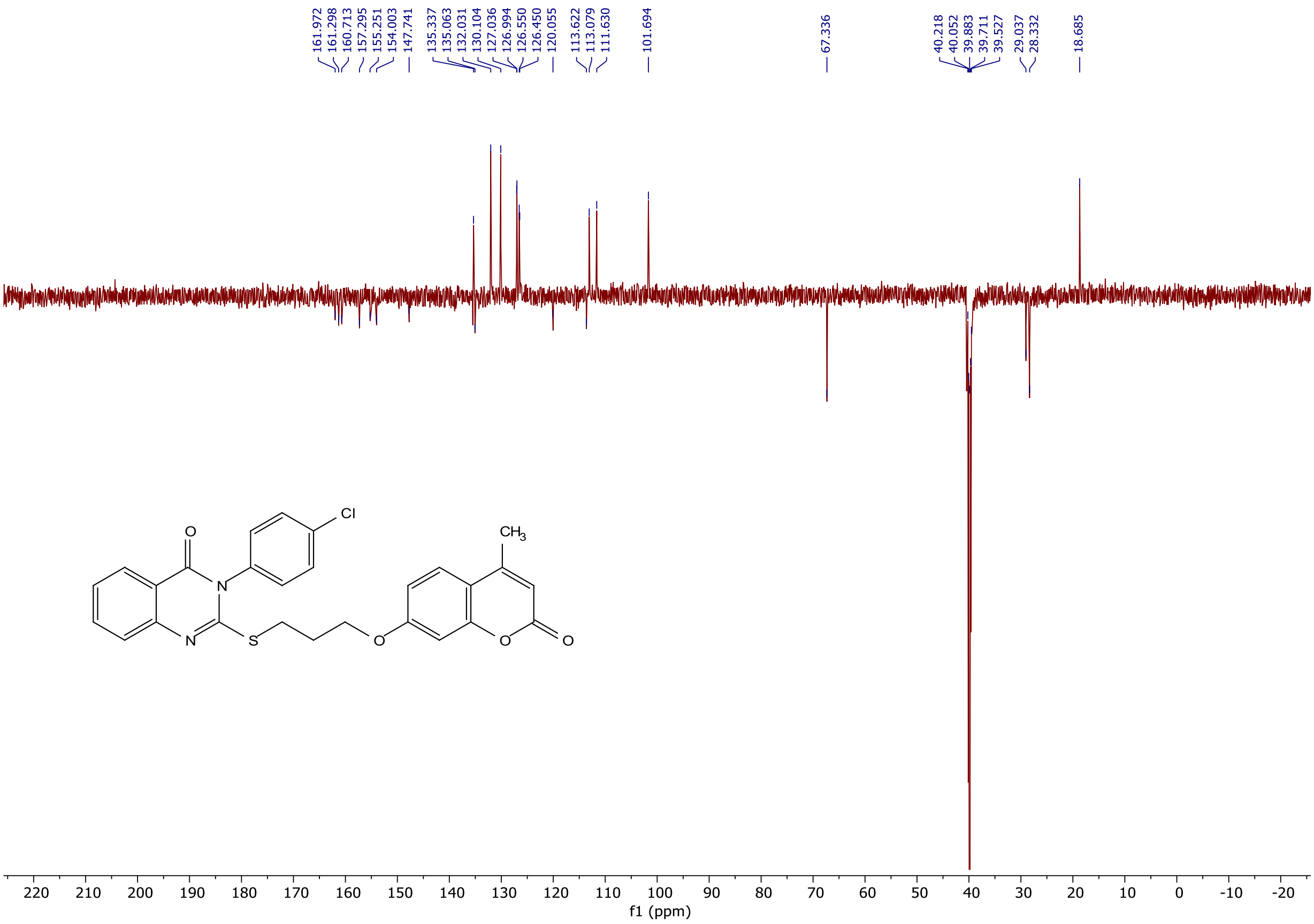

Figure S20. ^{13}C NMR-DEPTQ (125 MHz, DMSO-d_6) spectrum of compound **4g**

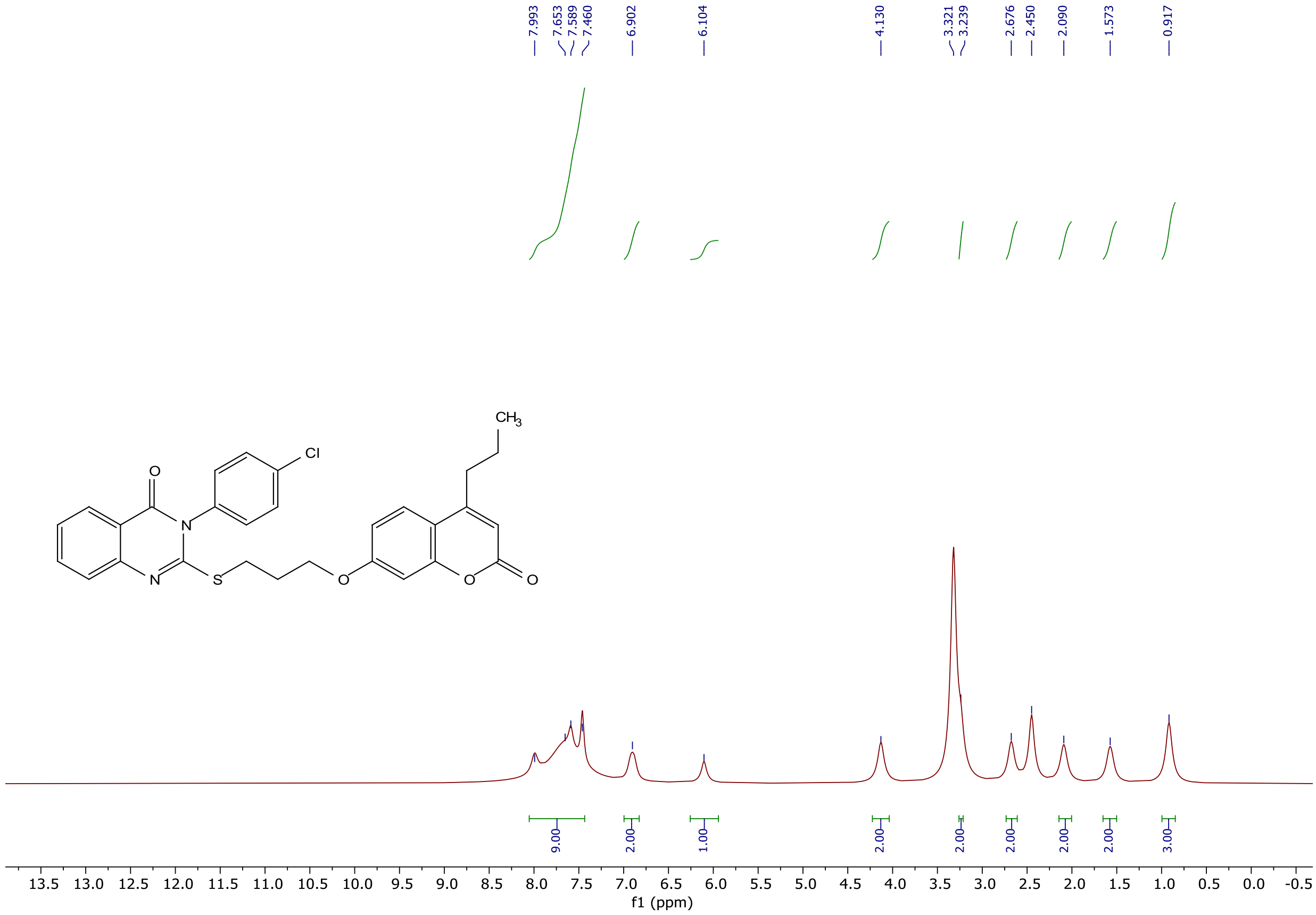

Figure S21. ^{1}H NMR (500 MHz, DMSO-d_6) spectrum of compound **4h**

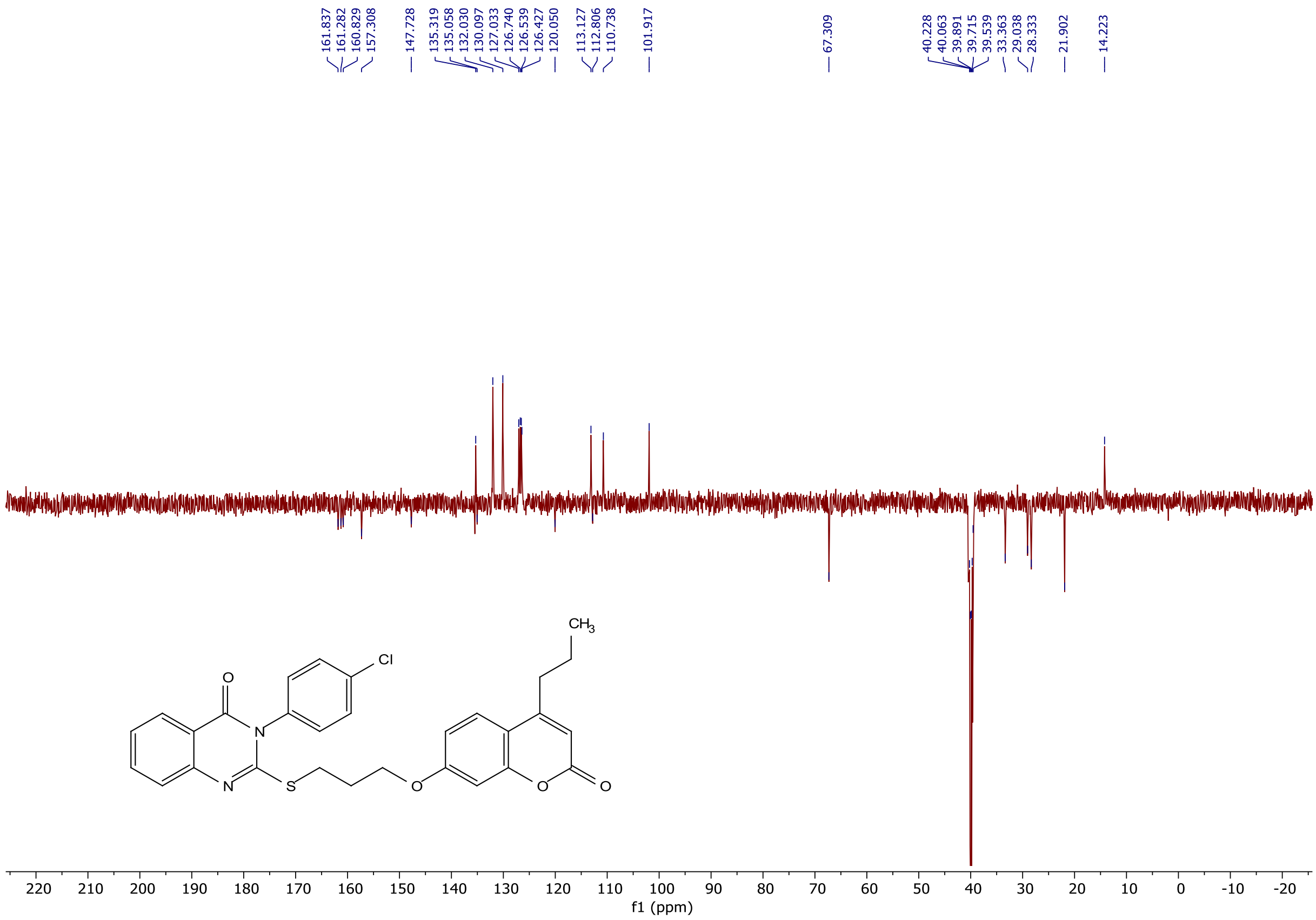

Figure S22. ^{13}C NMR-DEPTQ (125 MHz, DMSO-d_6) spectrum of compound **4h**

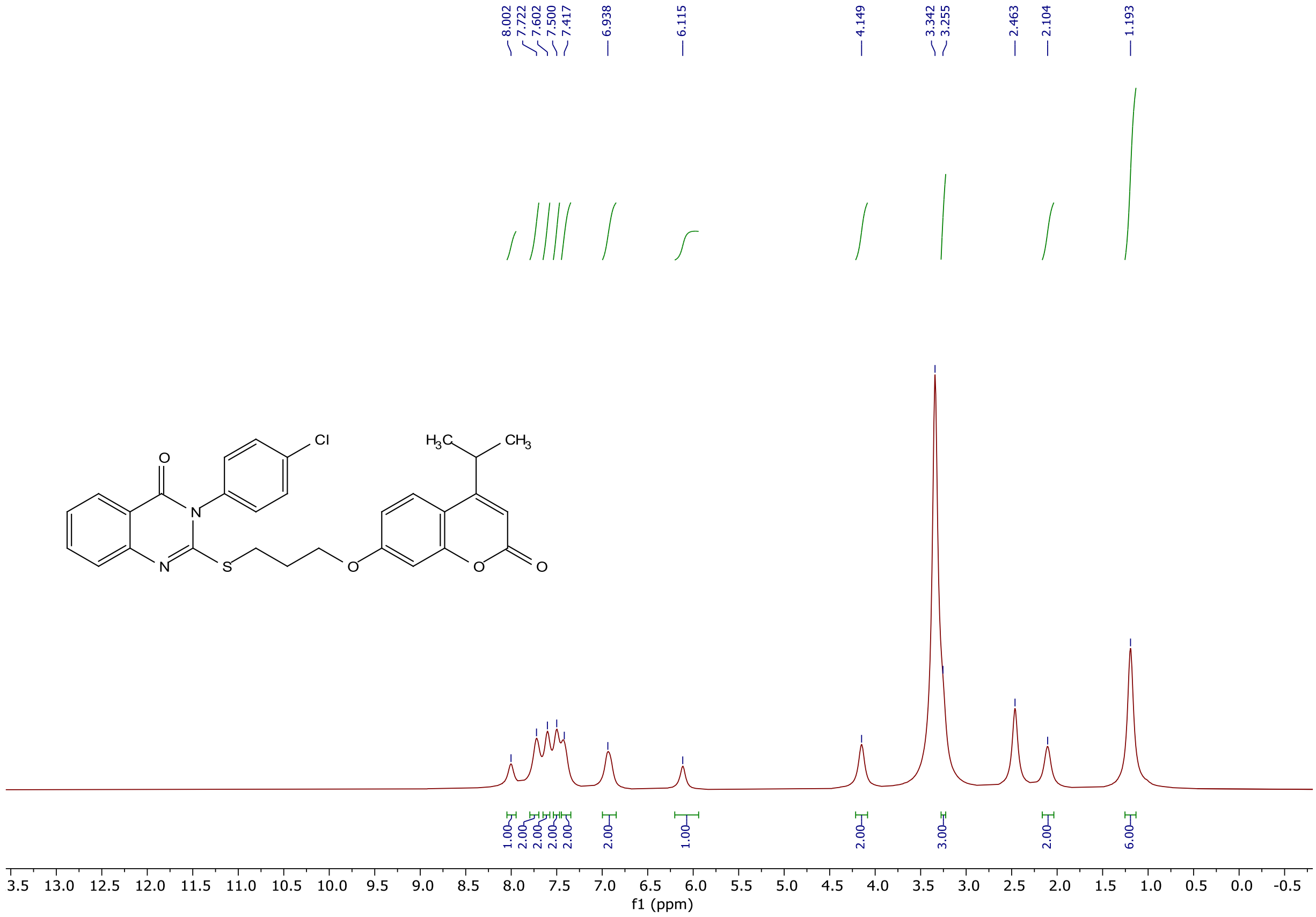

Figure S23. ^{1}H NMR (500 MHz, DMSO-d_6) spectrum of compound **4i**

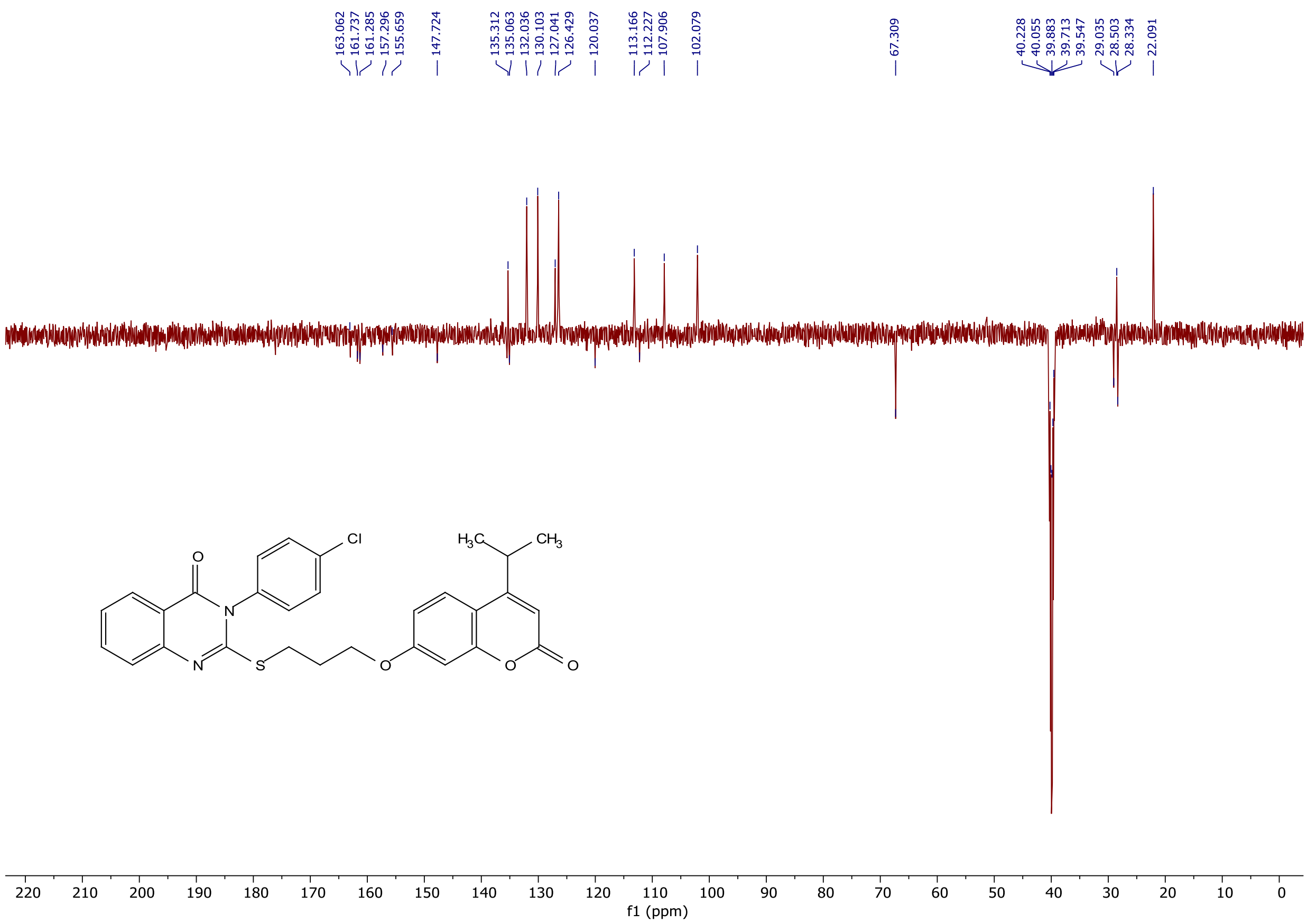

Figure S24. ^{13}C NMR-DEPTQ (125 MHz, DMSO-d_6) spectrum of compound **4i**

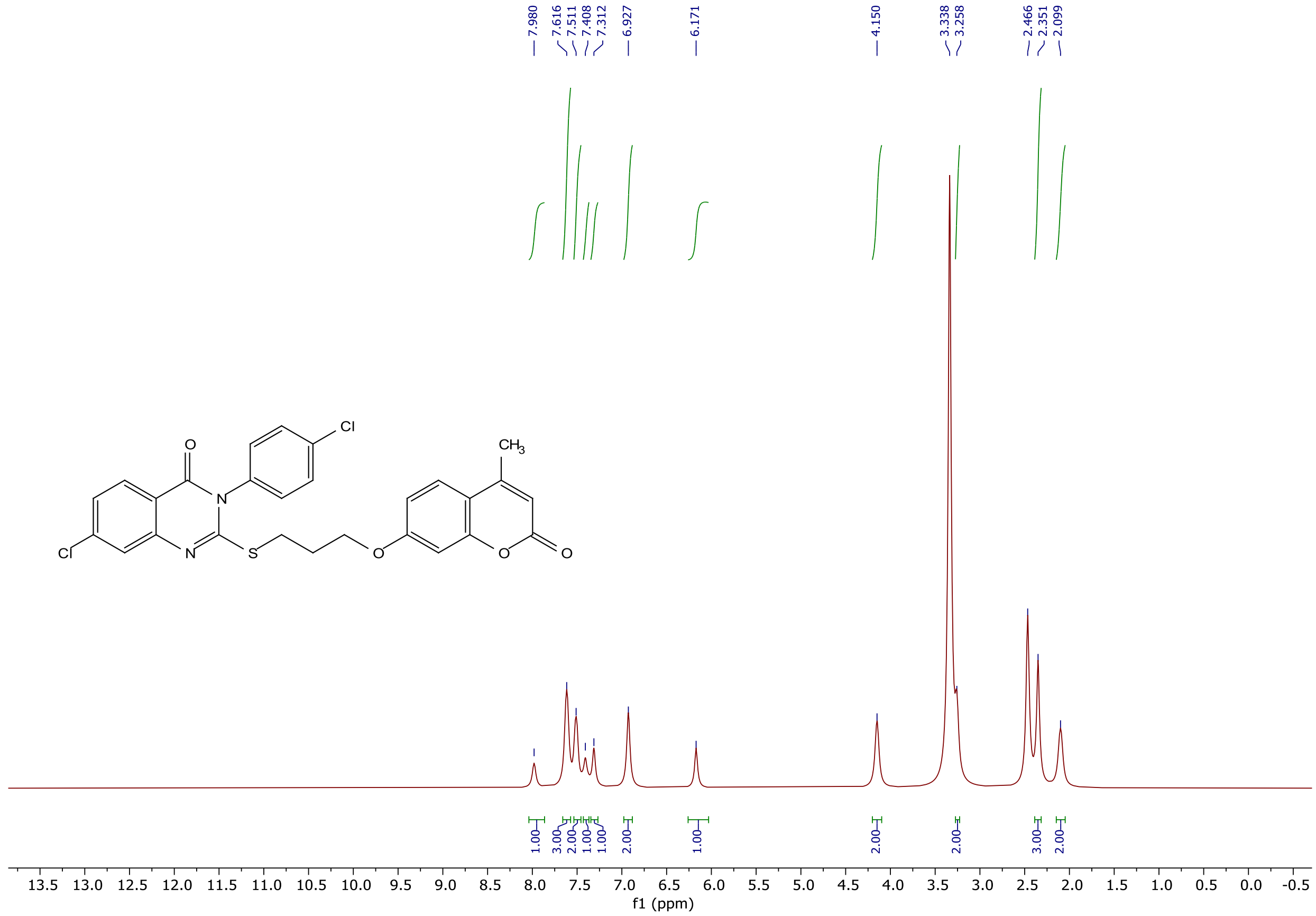

Figure S25. ^{1}H NMR (500 MHz, DMSO-d_6) spectrum of compound **4j**

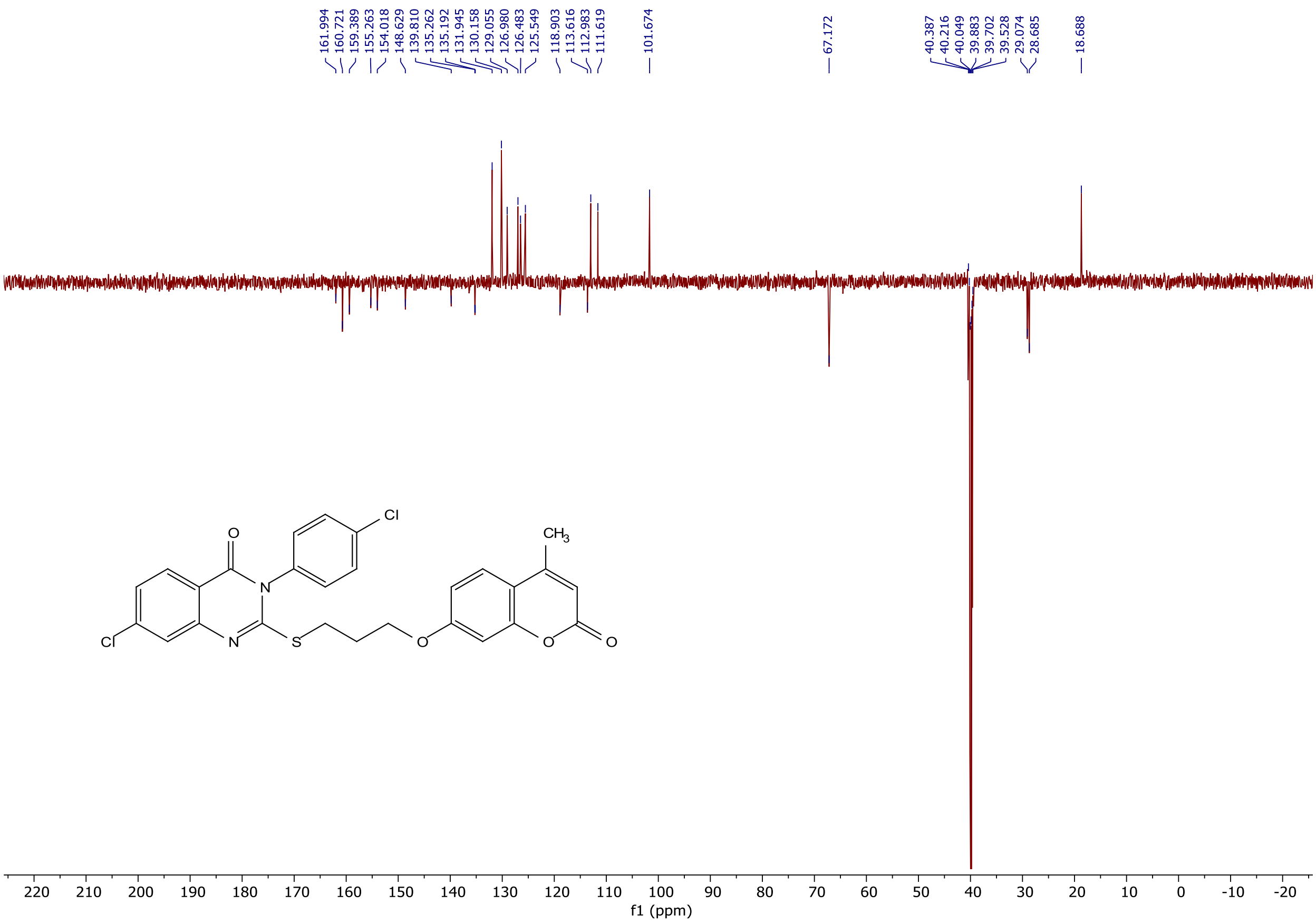

Figure S26. ^{13}C NMR-DEPTQ (125 MHz, DMSO-d_6) spectrum of compound **4j**

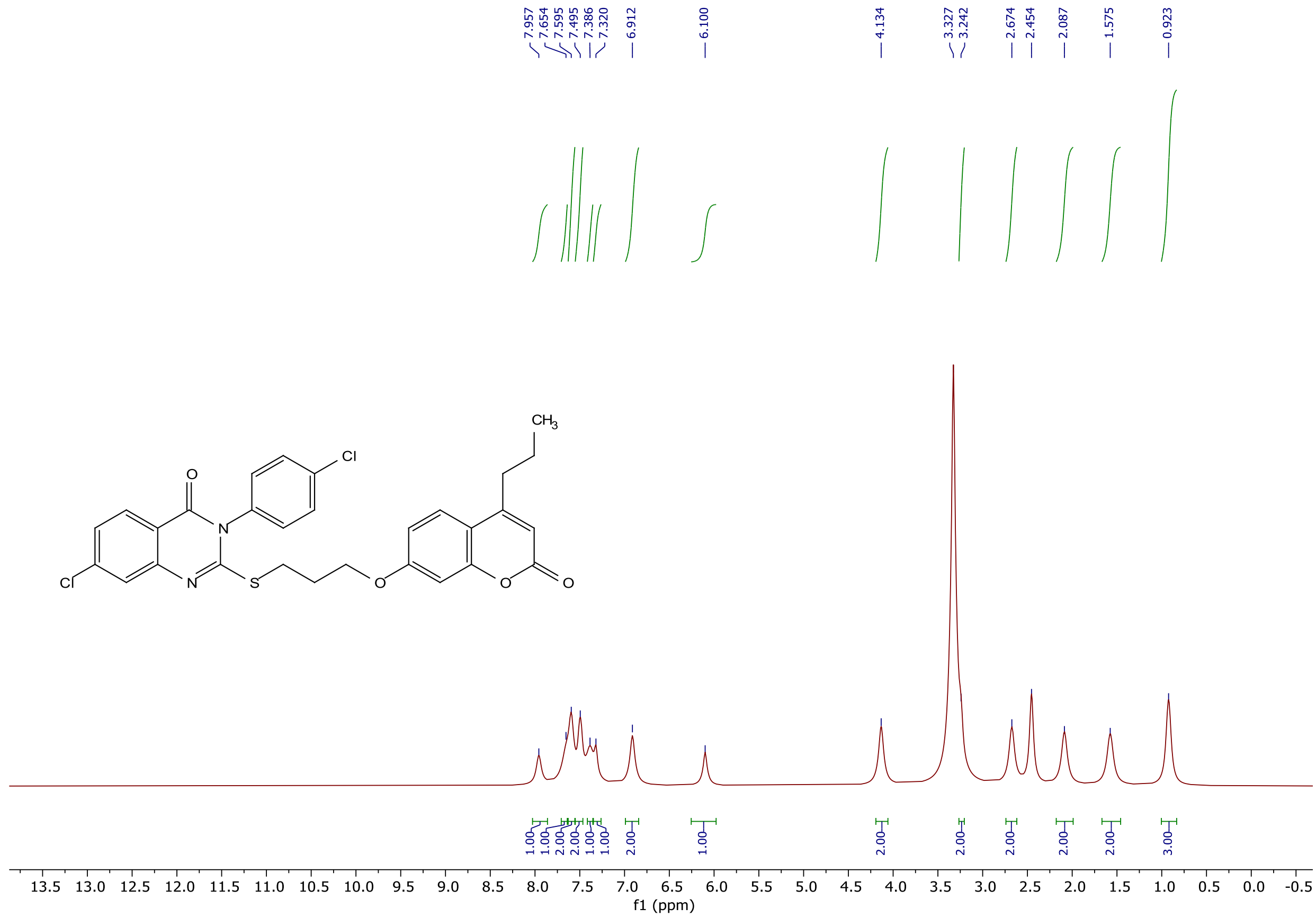

Figure S27. ^{1}H NMR (500 MHz, DMSO-d_6) spectrum of compound **4k**

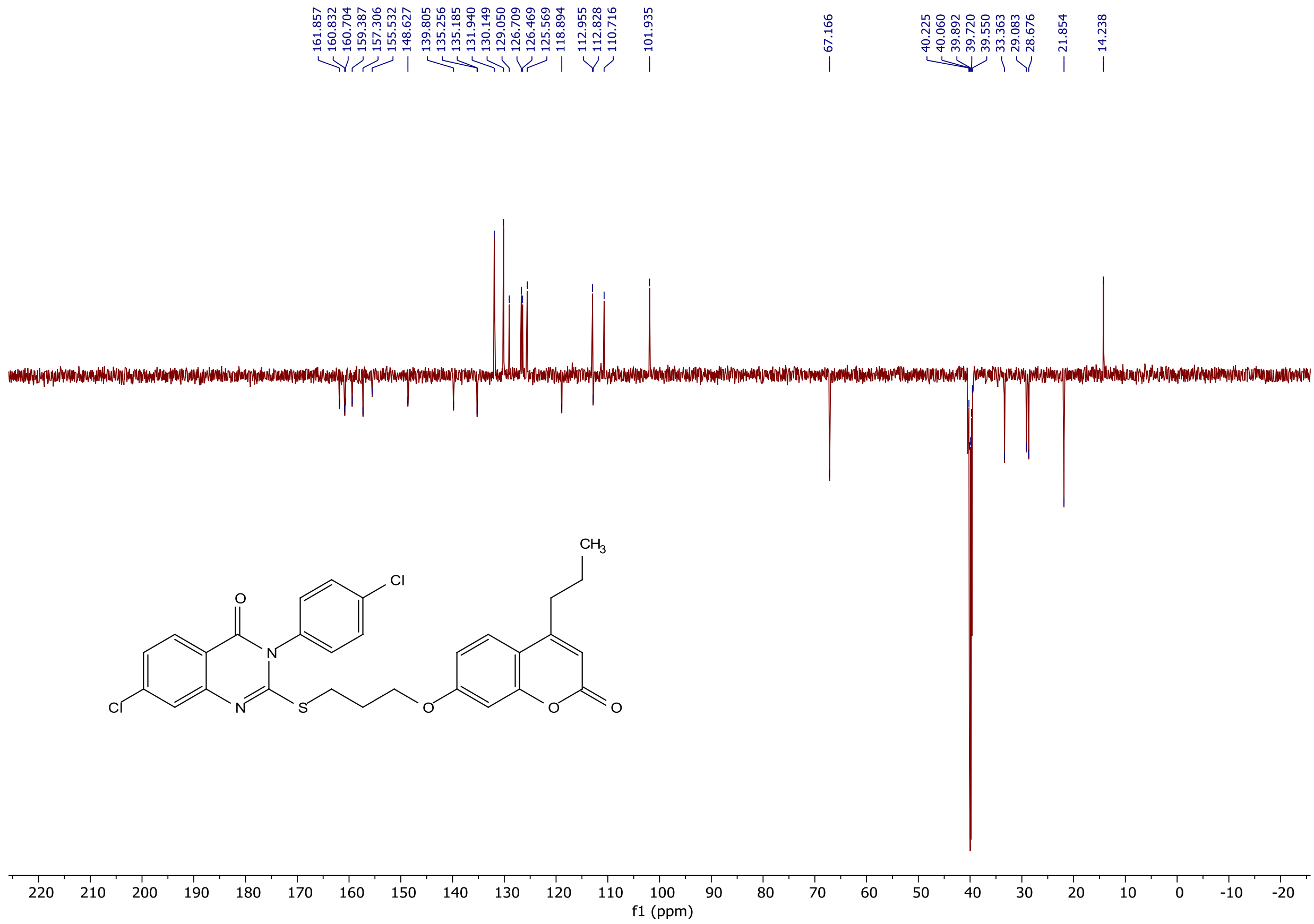

Figure S28. ^{13}C NMR-DEPTQ (125 MHz, DMSO-d_6) spectrum of compound **4k**

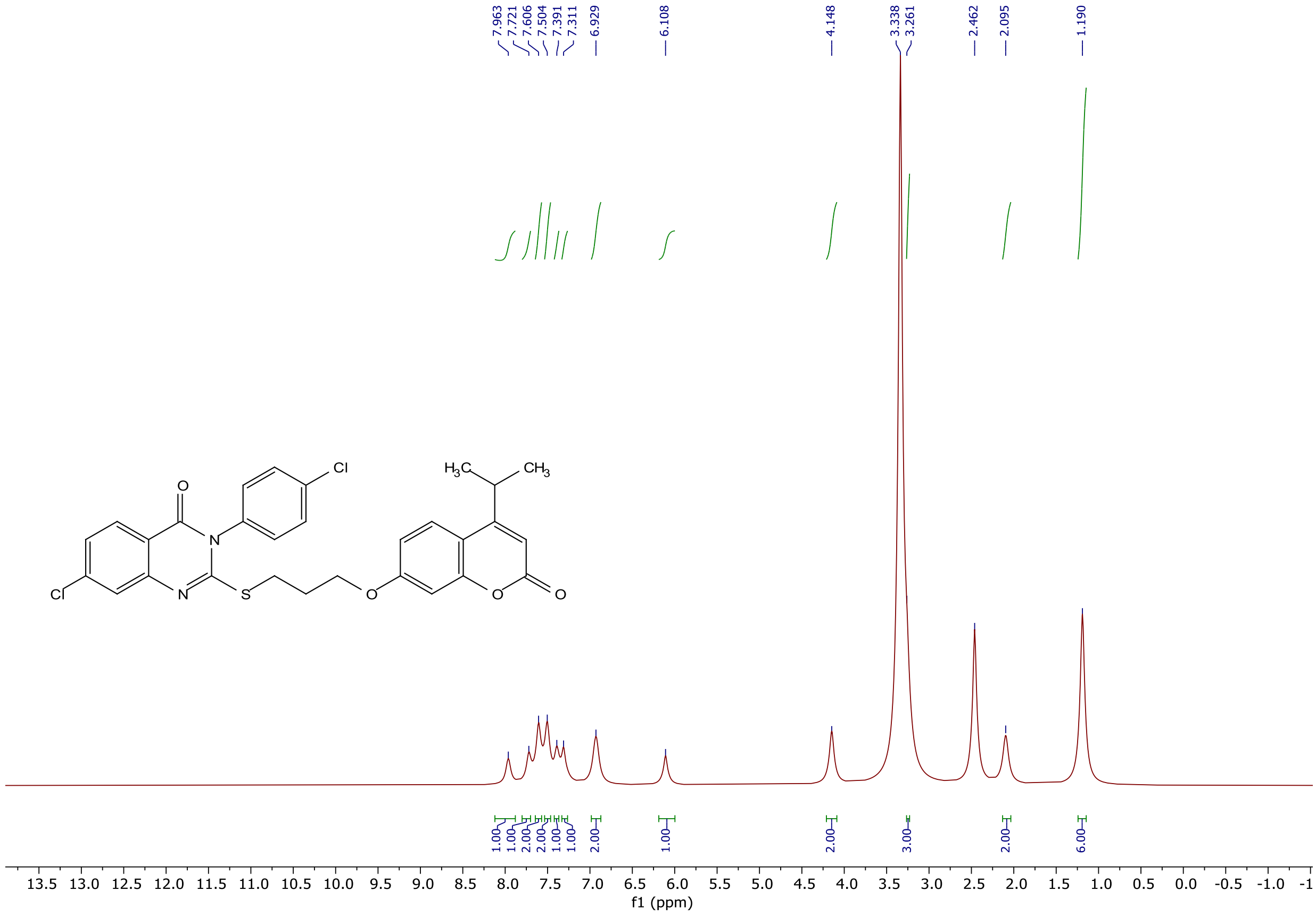

Figure S29. ^{1}H NMR (500 MHz, DMSO-d_6) spectrum of compound **4l**

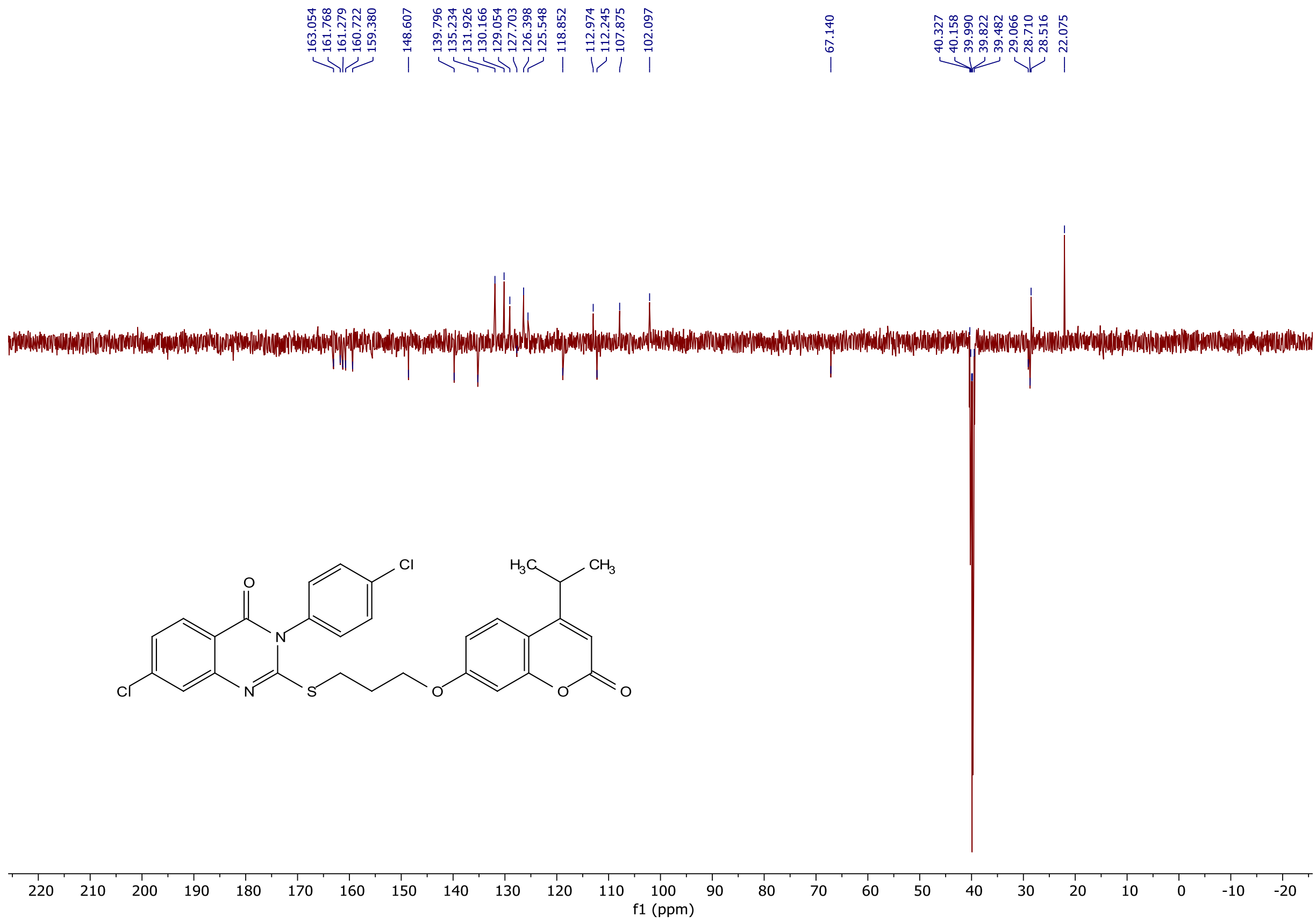

Figure S30. ^{13}C NMR-DEPTQ (125 MHz, DMSO-d_6) spectrum of compound **4l**

Mass spectral data of the target compounds (4a-l)

(A)

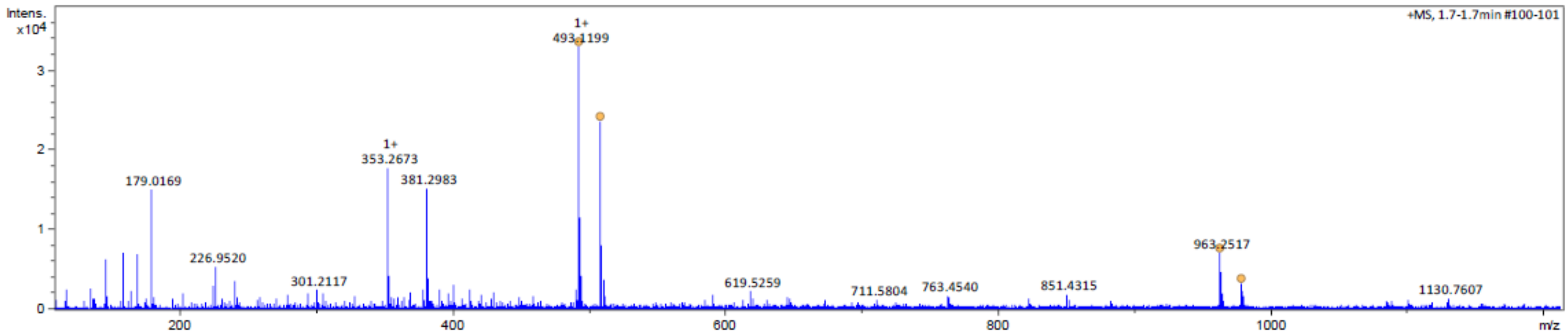

(B)

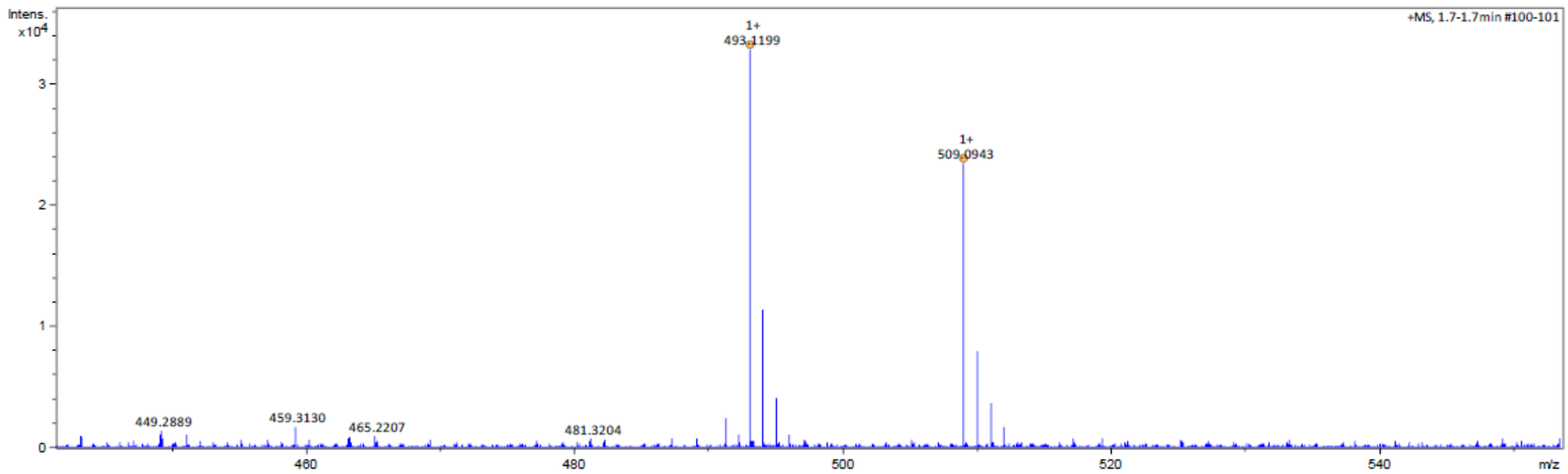

Figure S31. (A) Mass spectrum of compound **4a**, and **(B)** Zoom in spectra for range 460-540.

(A)

(B)

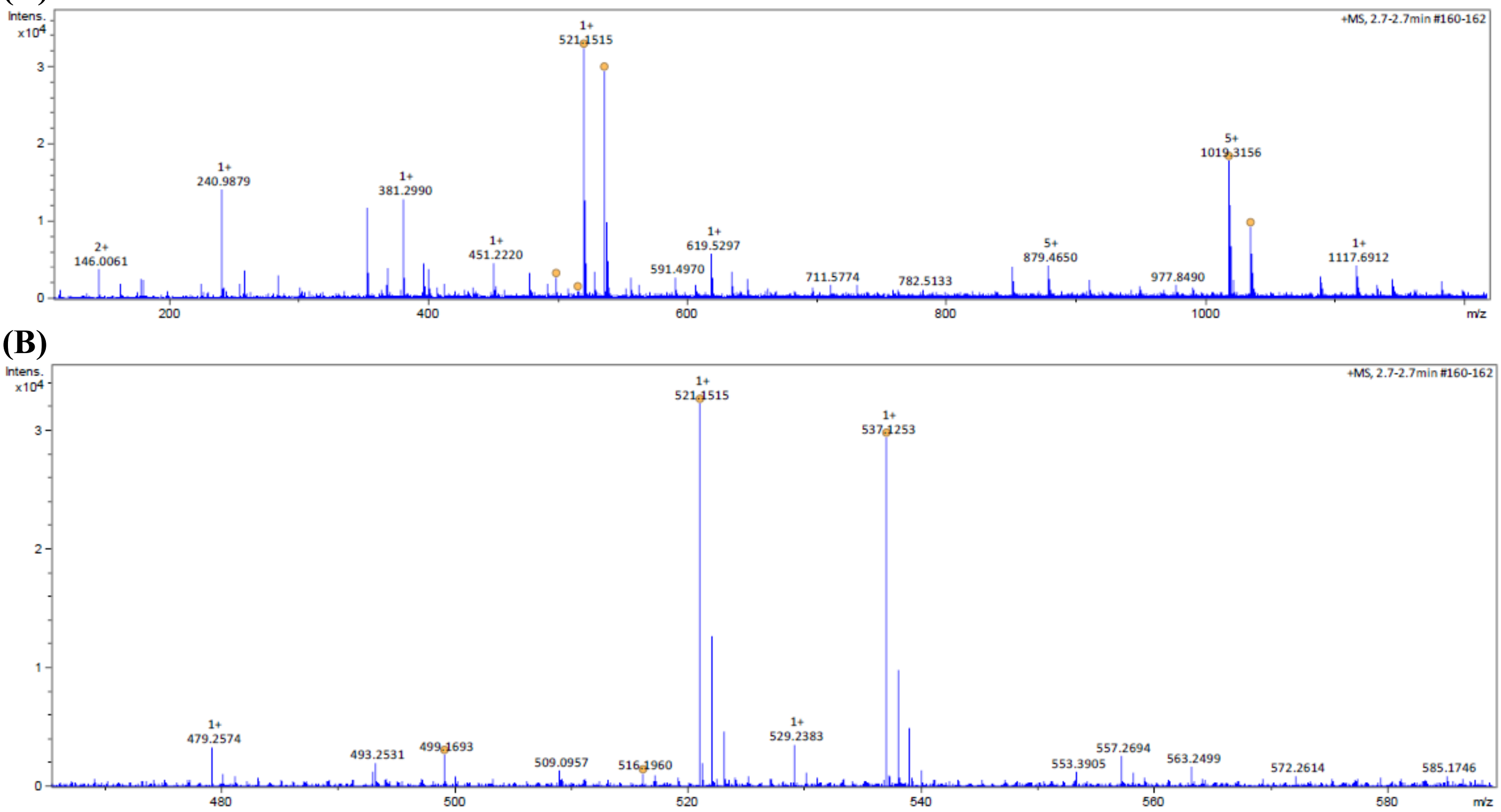

Figure S32. (A) Mass spectrum of compound **4b**, and **(B)** Zoom in spectra for range 480-580.

(A)

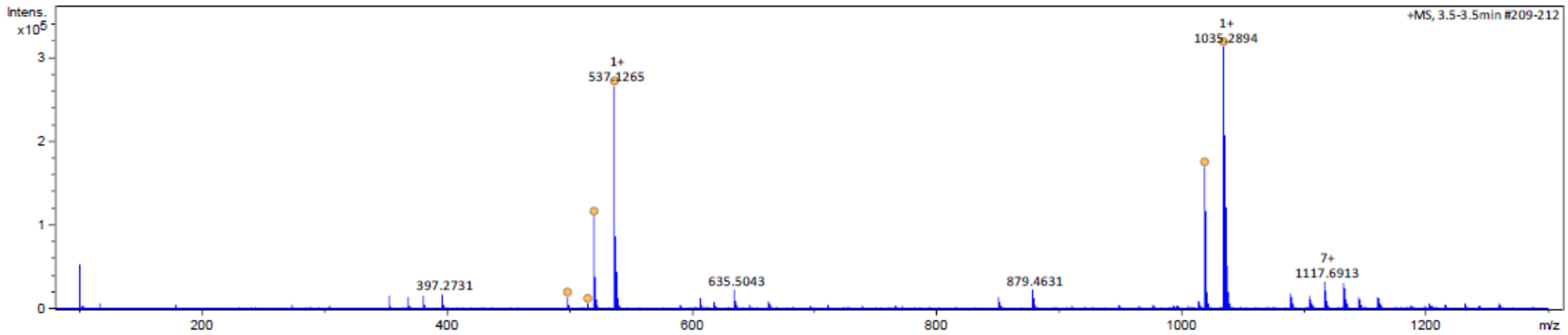

(B)

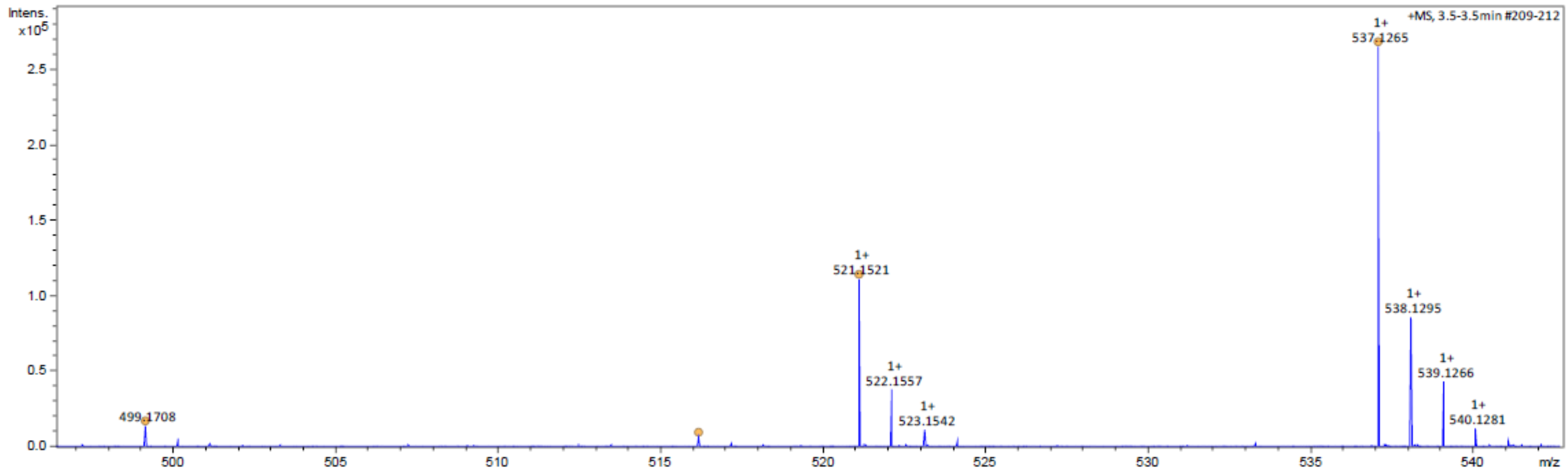

Figure S33. (A) Mass spectrum of compound **4c**, and **(B)** Zoom in spectra for range 500-540.

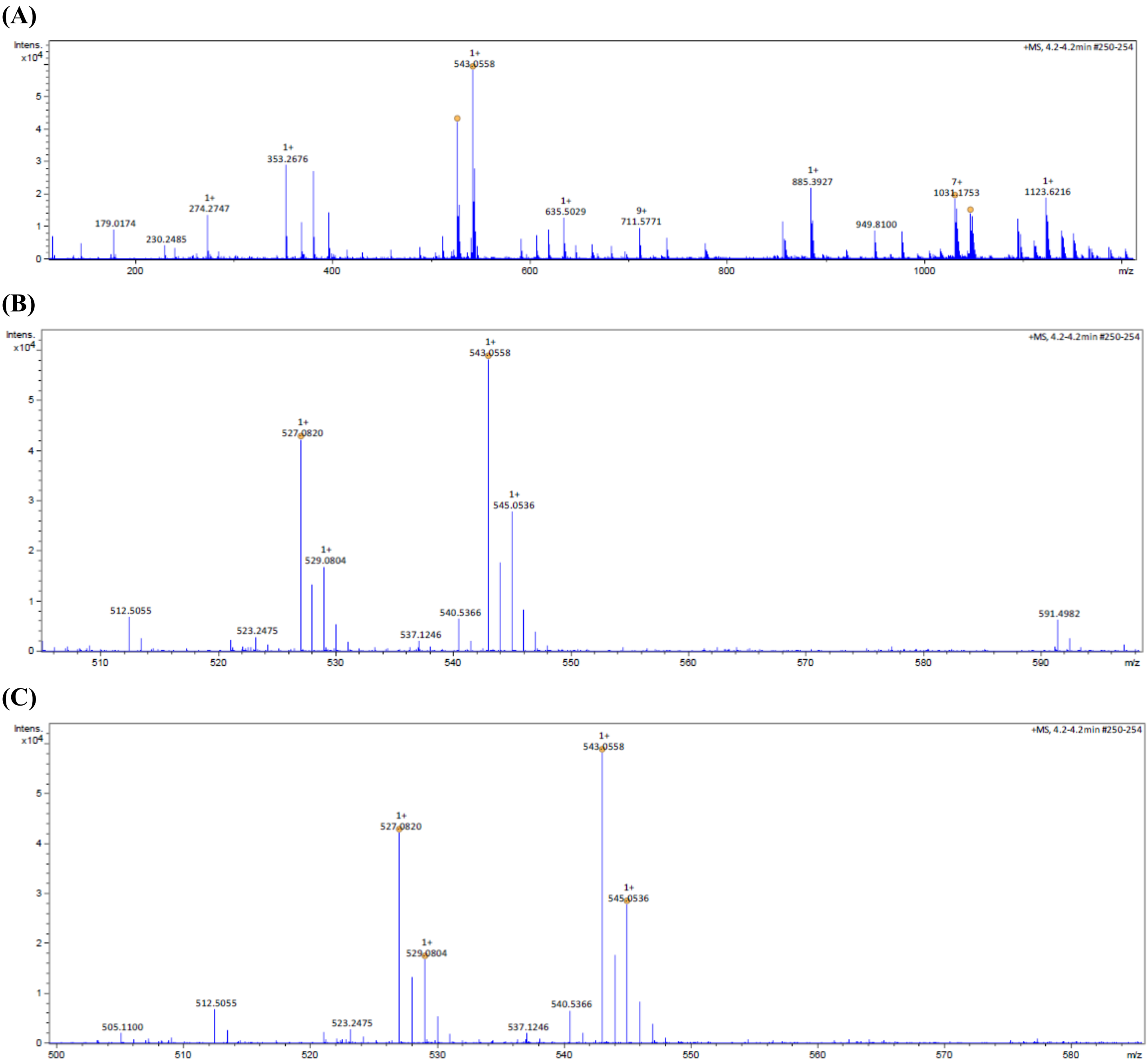

Figure S34. (A) Mass spectrum of compound **4d**, **(B)** Zoom in spectra for range 510-590, and **(C)** Isotopic profile of chloride.

(A)

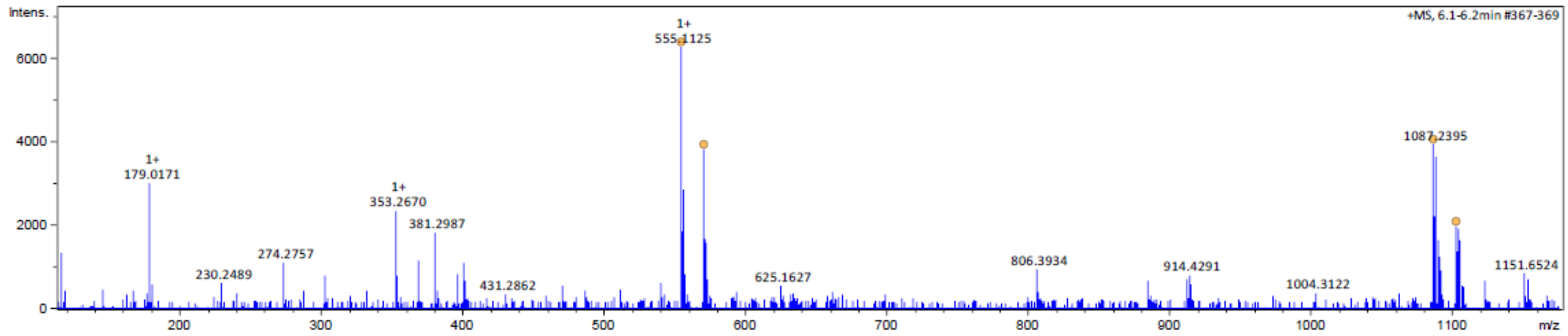

(B)

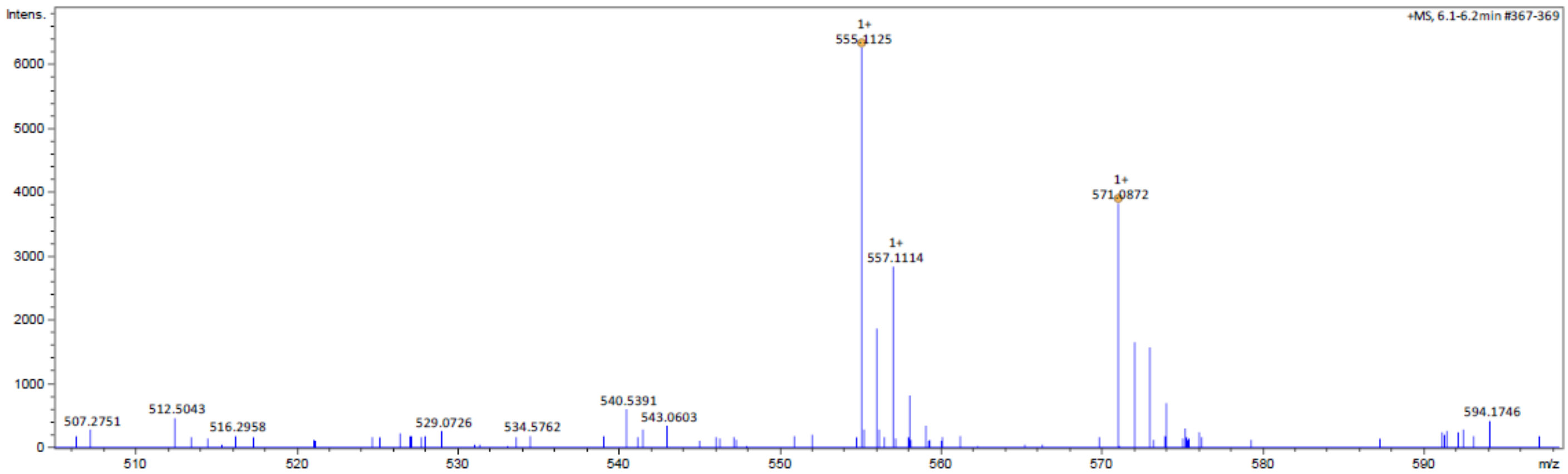

(C)

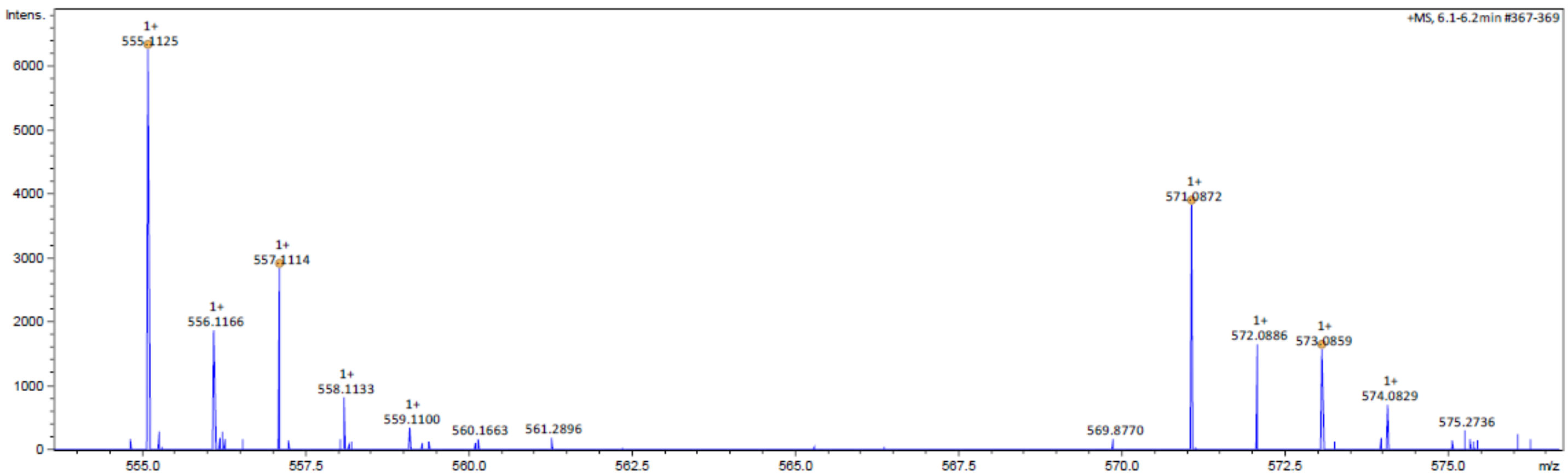

Figure S35. (A) Mass spectrum of compound **4e**, **(B)** Zoom in spectra for range 510-590, and **(C)** Isotopic profile of chloride.

(A)

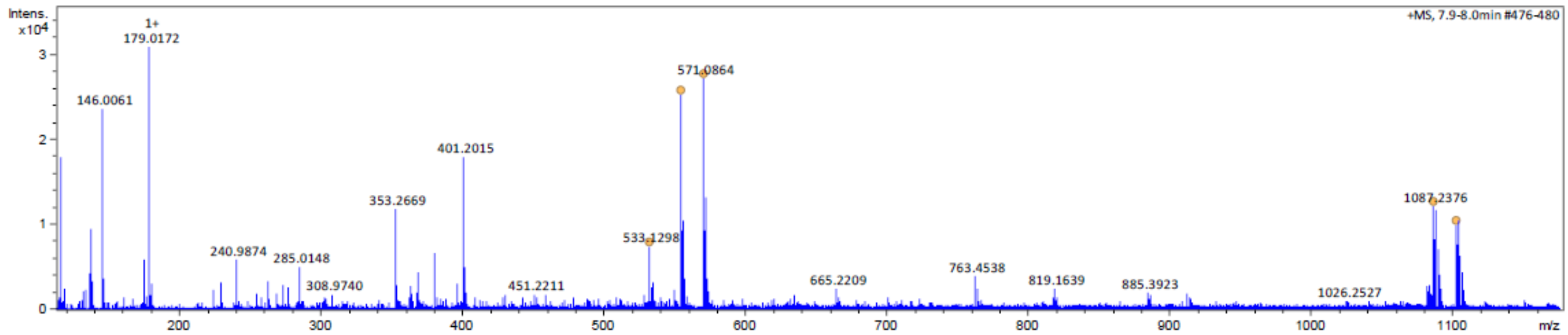

(B)

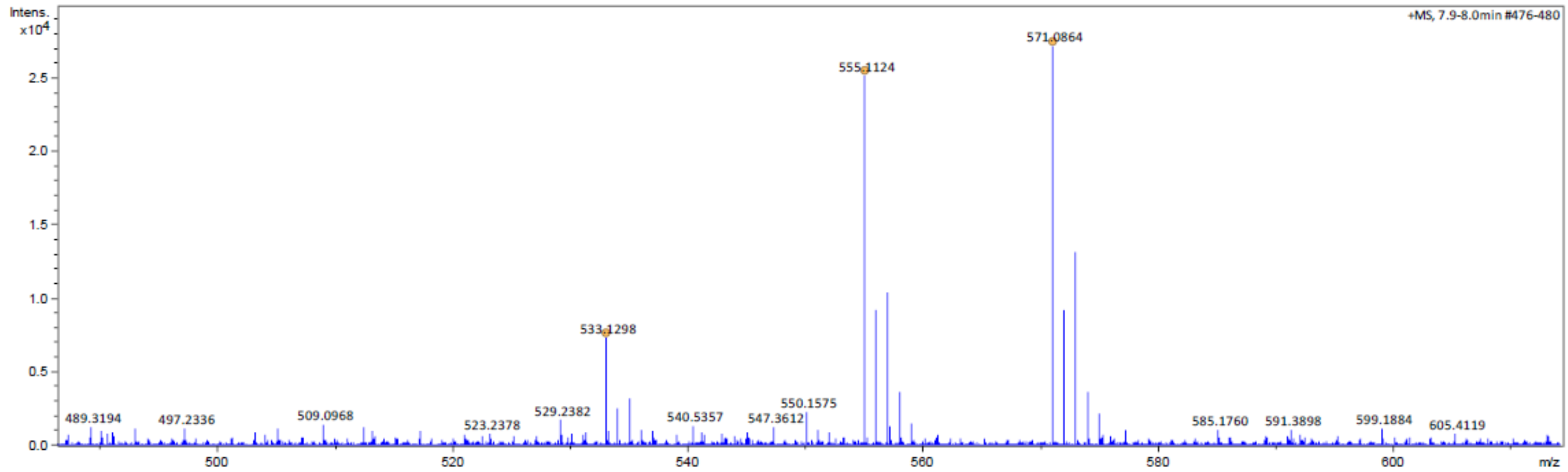

(C)

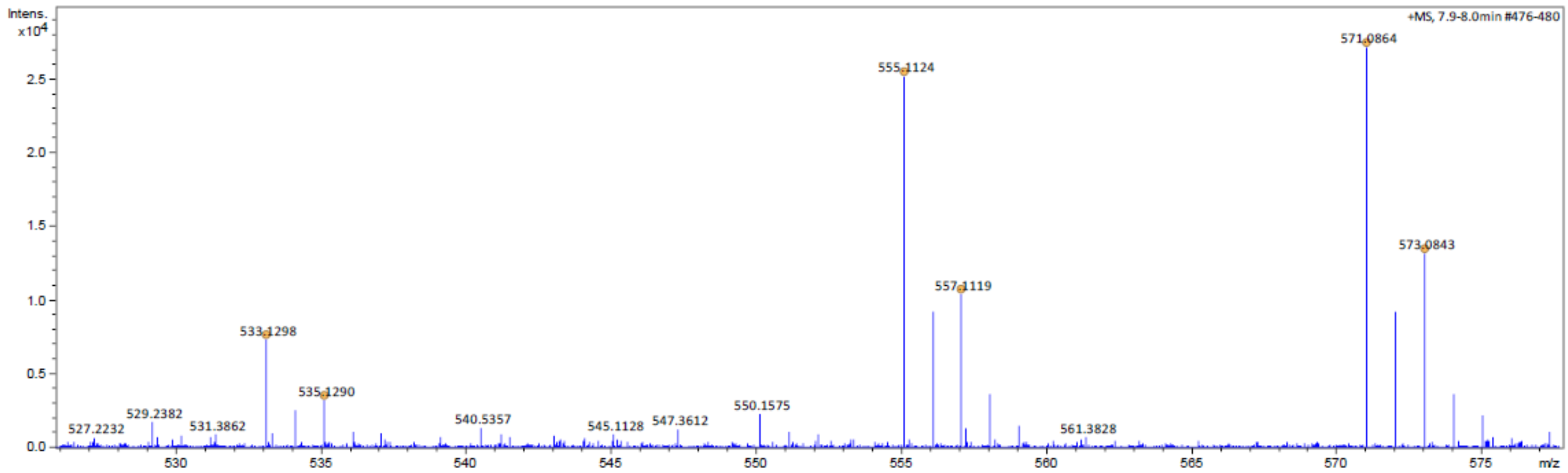

Figure S36. (A) Mass spectrum of compound **4f**, **(B)** Zoom in spectra for range 500-600, and **(C)** Isotopic profile of chloride.

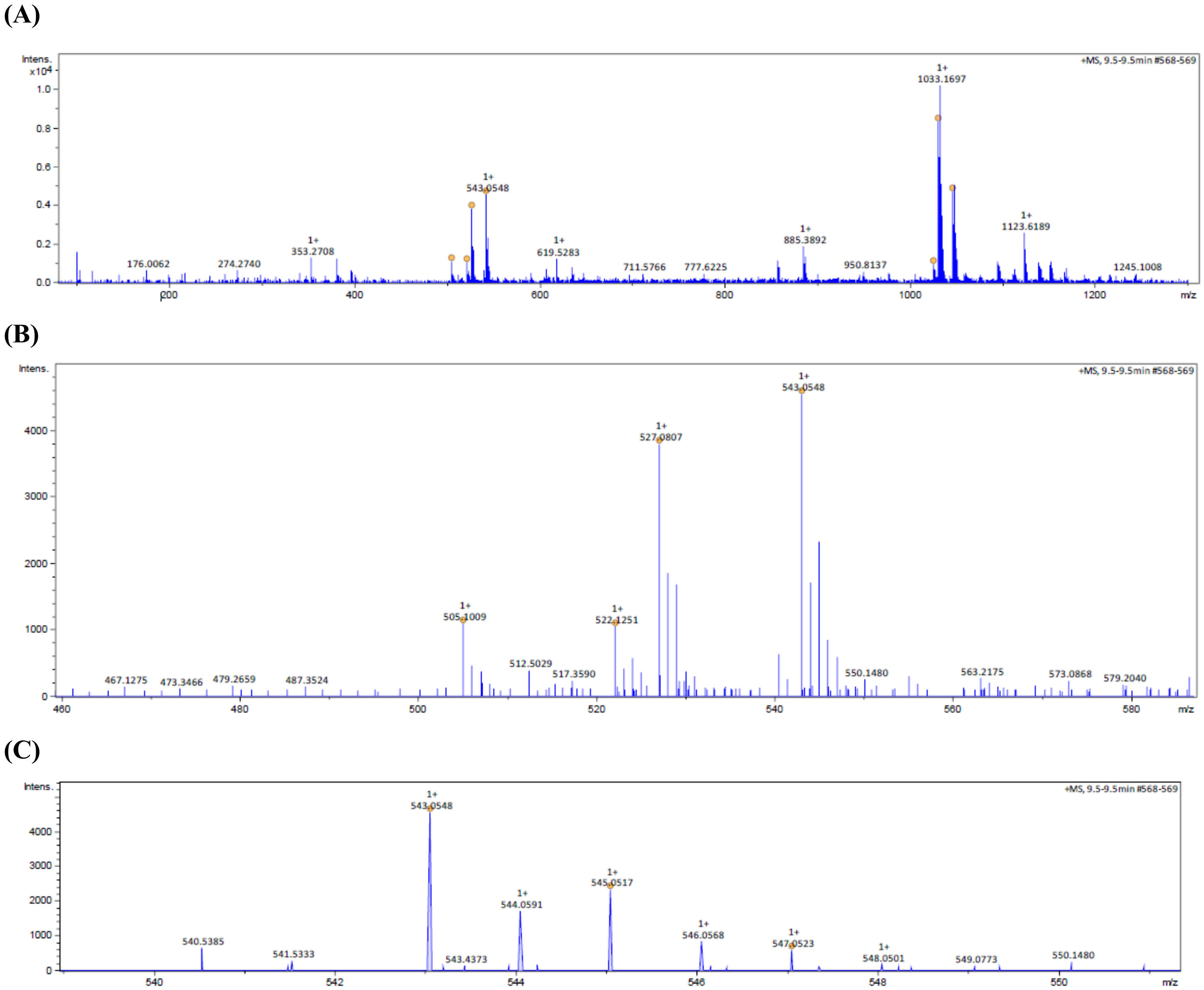

Figure S37. (A) Mass spectrum of compound **4g**, **(B)** Zoom in spectra for range 460-580, and **(C)** Isotopic profile of chloride.

(A)

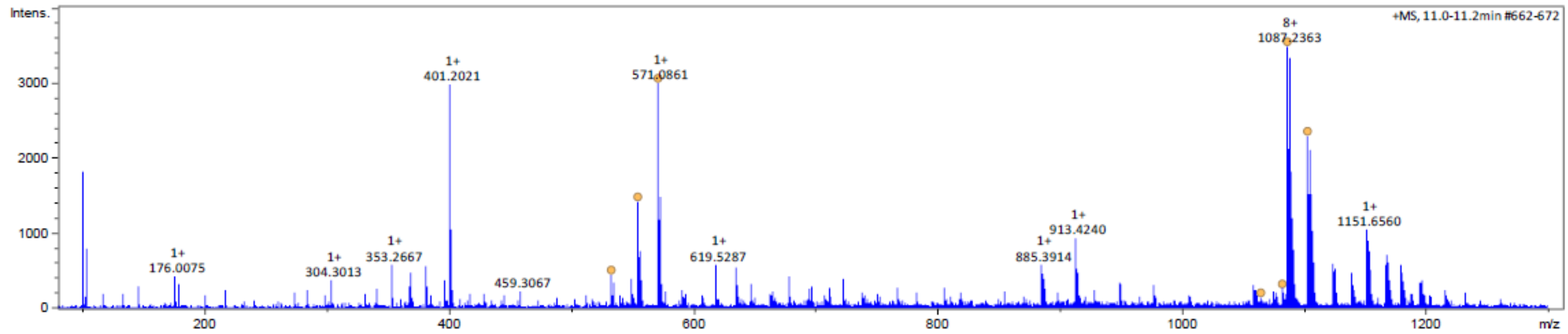

(B)

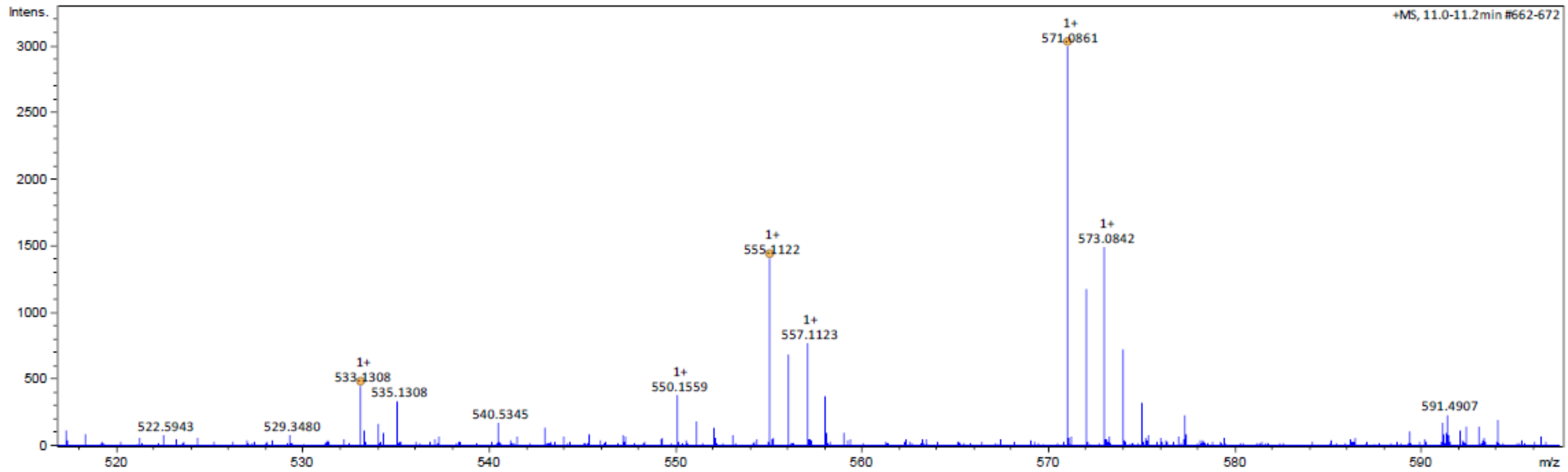

(C)

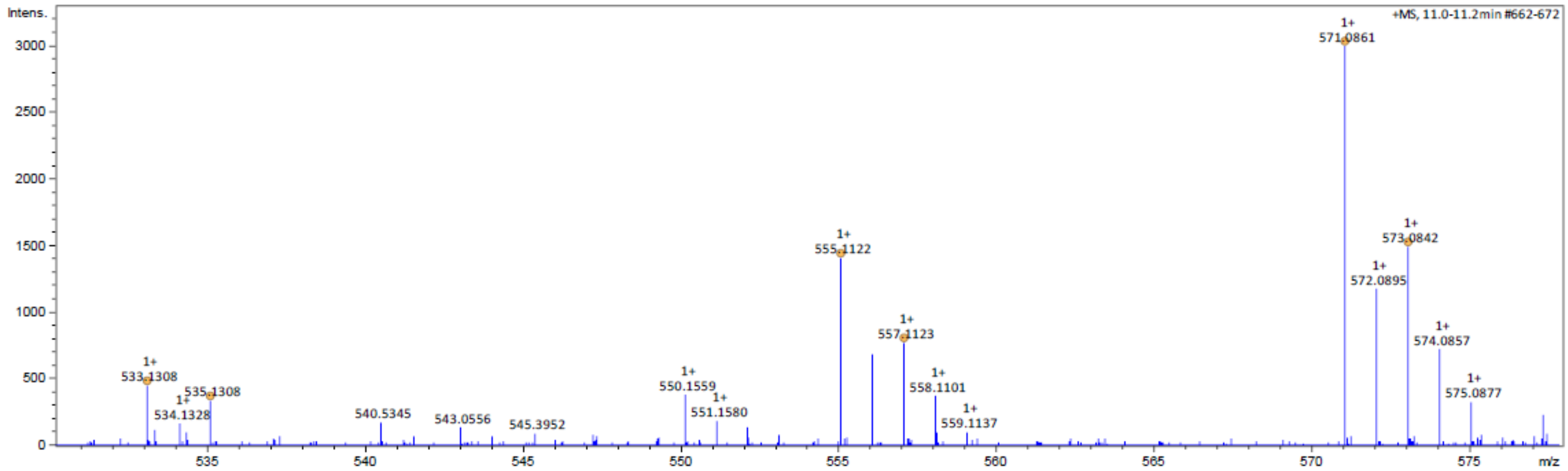

Figure S38. (A) Mass spectrum of compound **4h**, **(B)** Zoom in spectra for range 520-590, and **(C)** Isotopic profile of chloride.

(A)

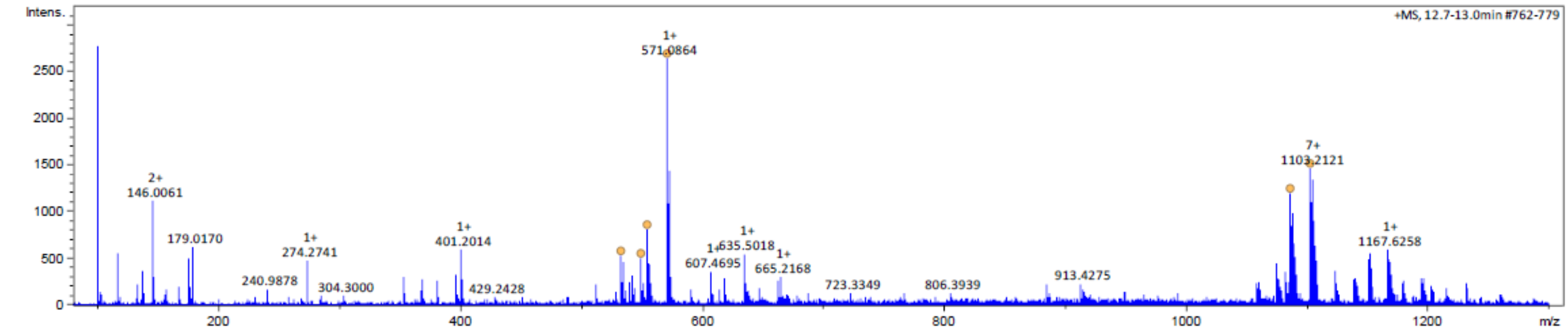

(B)

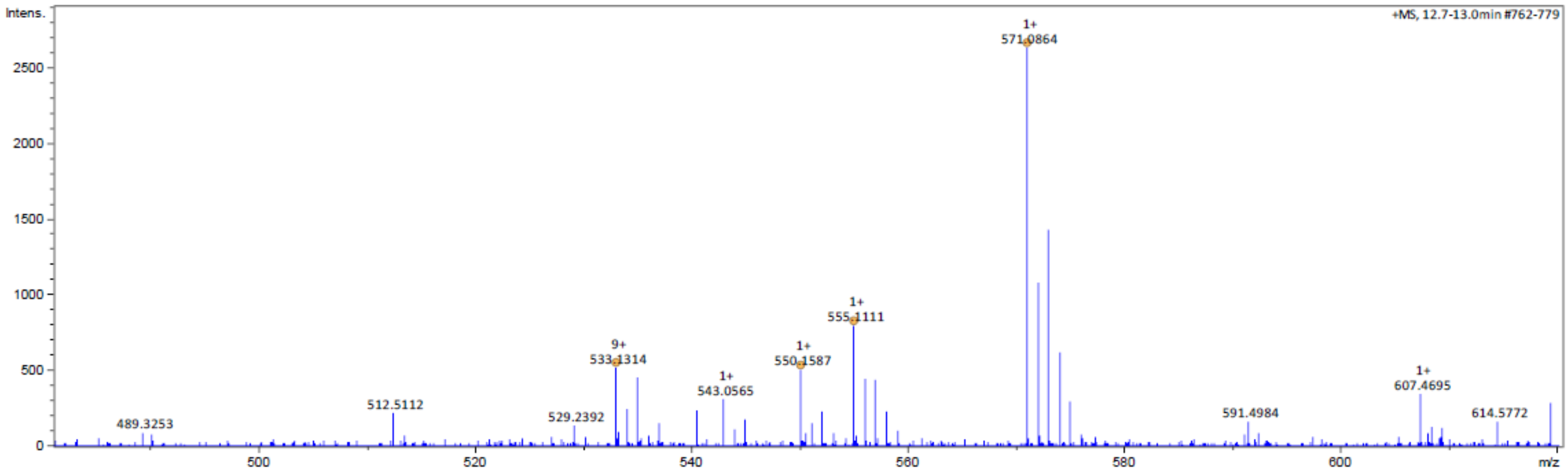

(C)

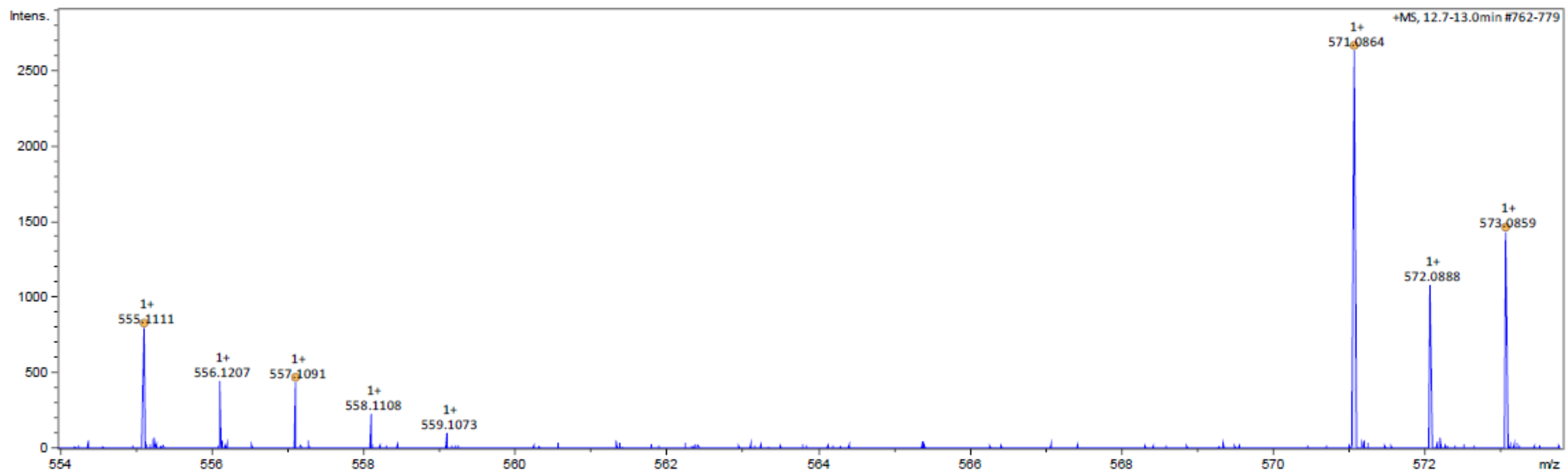

Figure S39. (A) Mass spectrum of compound **4i**, **(B)** Zoom in spectra for range 500-600, and **(C)** Isotopic profile of chloride.

(A)

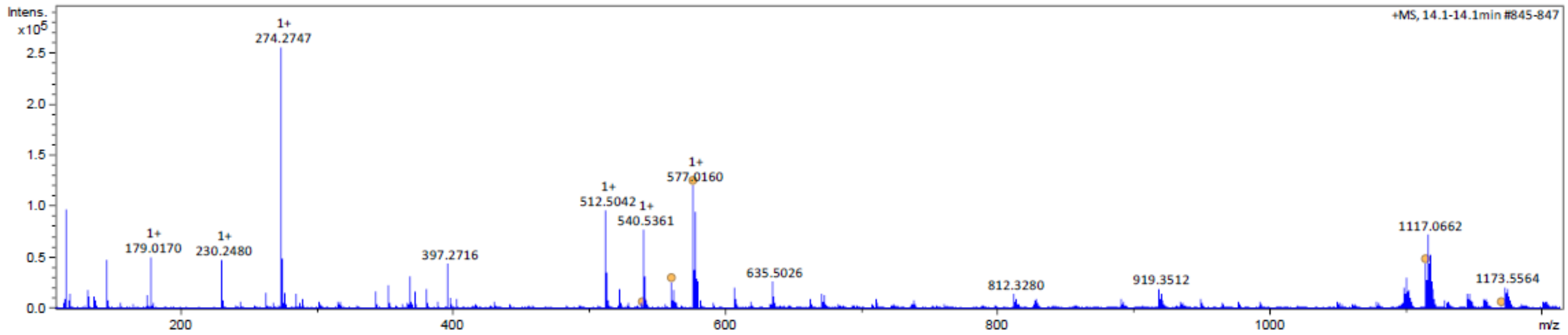

(B)

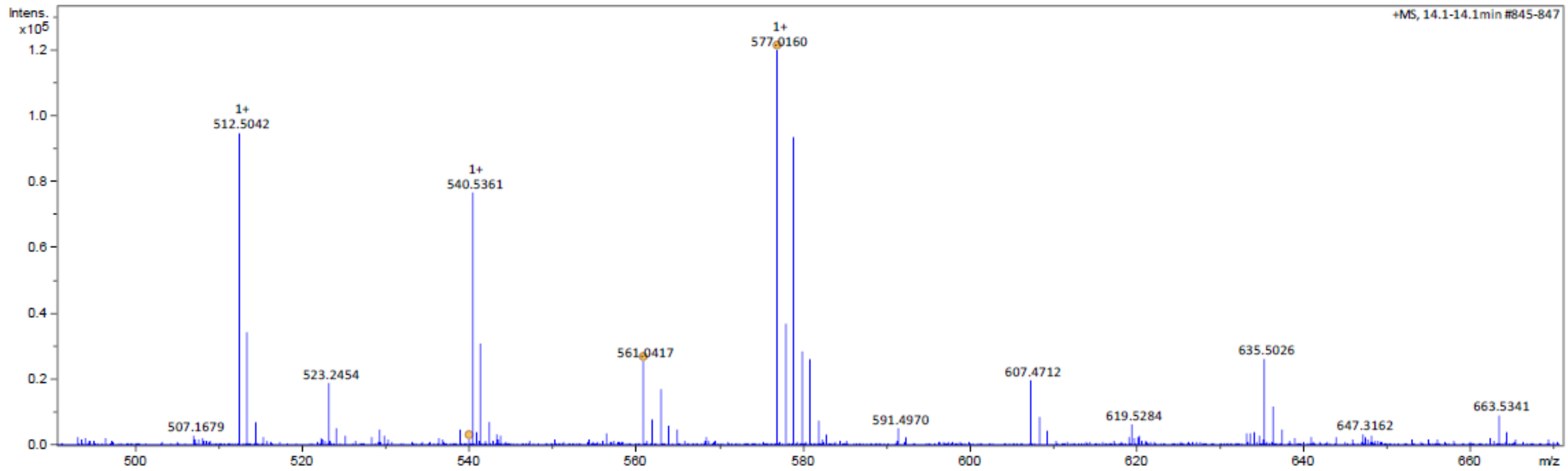

(C)

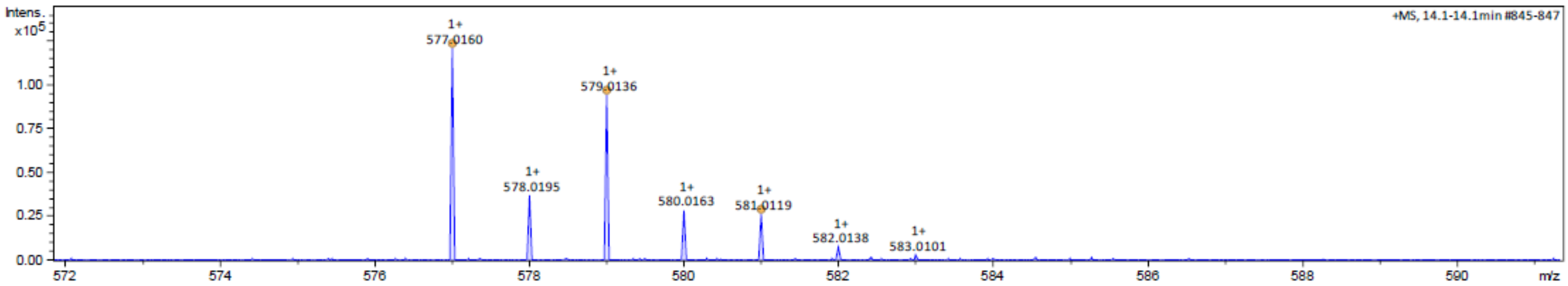

Figure S40. (A) Mass spectrum of compound **4j**, **(B)** Zoom in spectra for range 500-660, and **(C)** Isotopic profile of chloride.

(A)

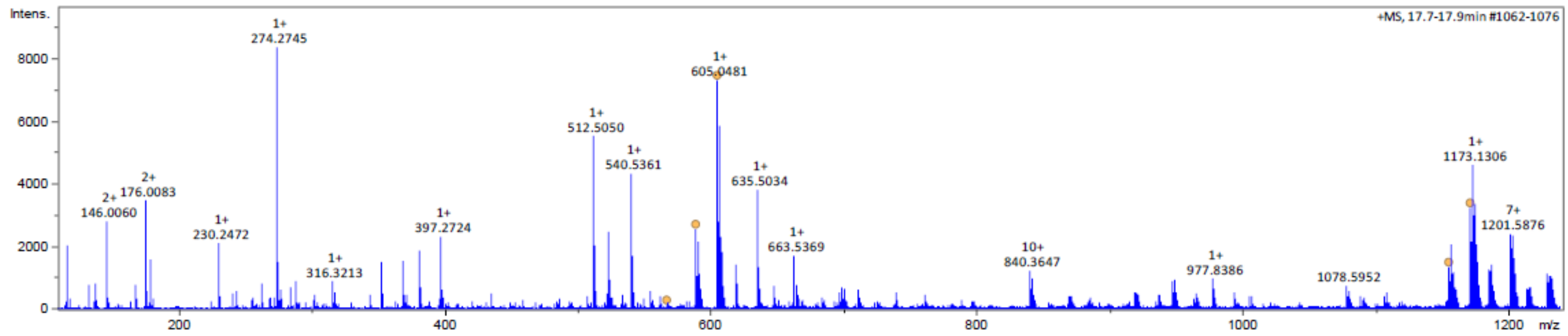

(B)

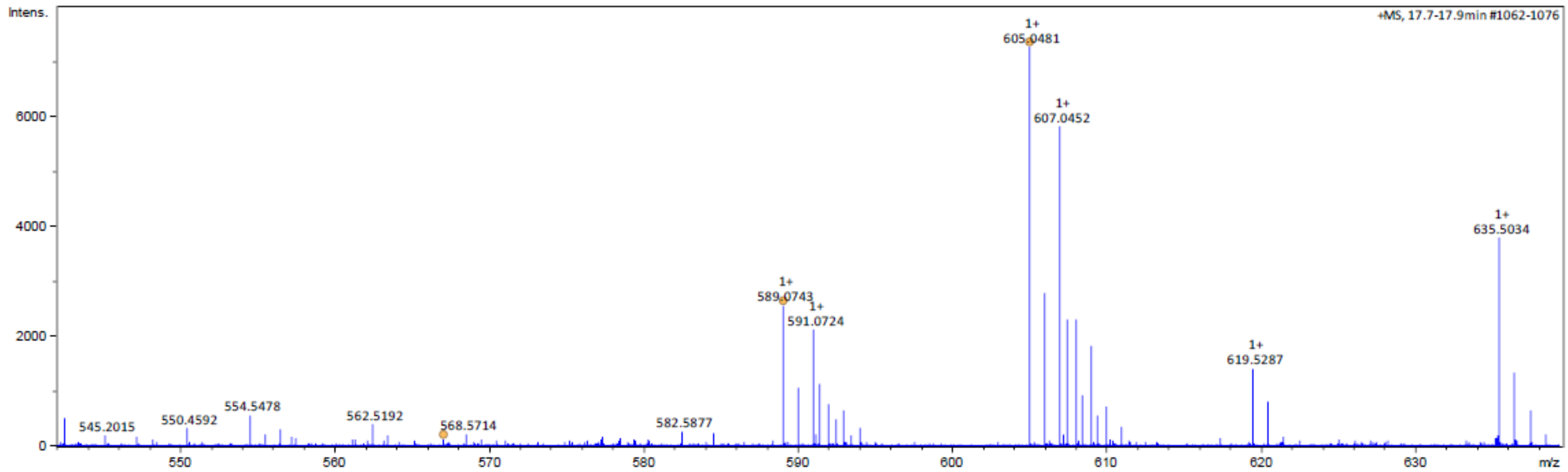

(C)

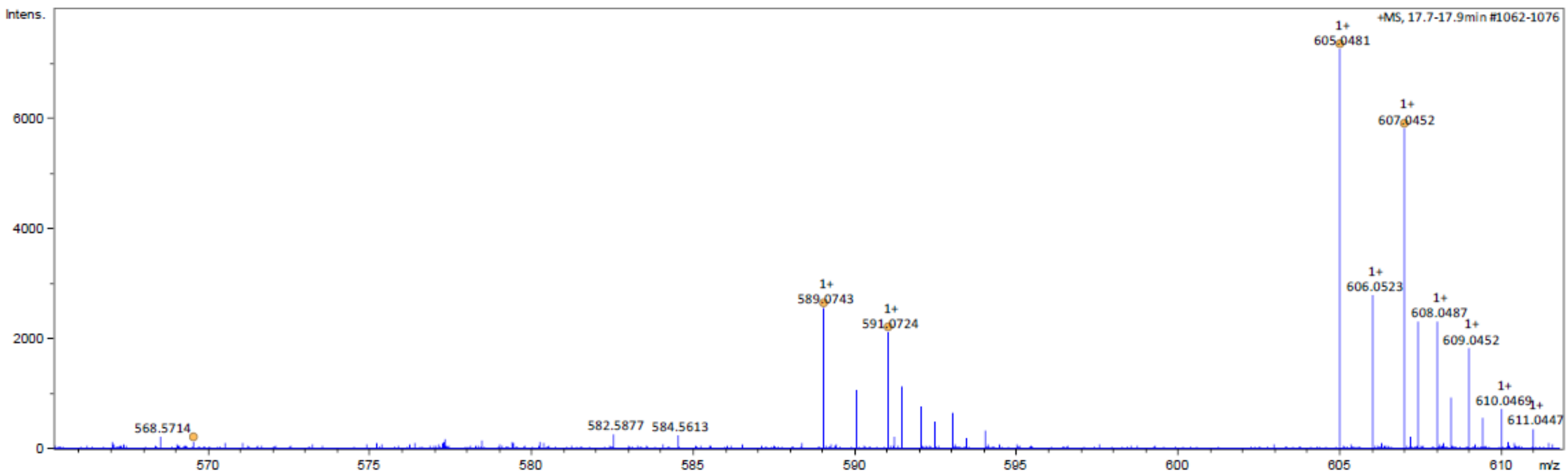

Figure S41. **(A)** Mass spectrum of compound **4k**, **(B)** Zoom in spectra for range 550-630, and **(C)** Isotopic profile of chloride.

(A)

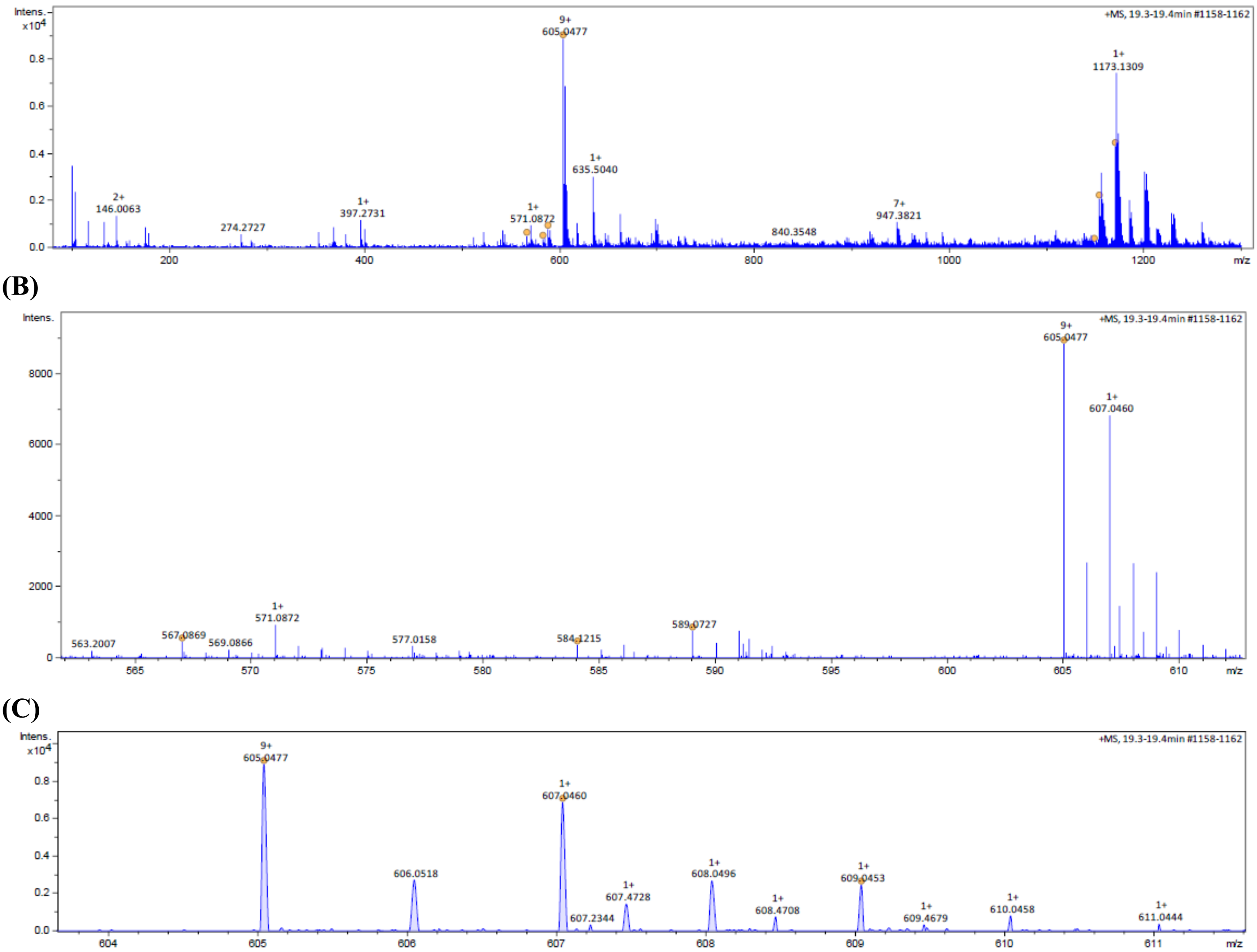

Figure S42. (A) Mass spectrum of compound **4l**, **(B)** Zoom in spectra for range 565-610, and **(C)** Isotopic profile of chloride.

HPLC purity

The target compounds were tested for purity using HPLC (Agilent™ 1260 Infinity HPLC system equipped with Agilent 1260 Infinity quaternary pump (G1311C), Agilent 1260 Infinity thermostated column compartment (G13b16A), Agilent 1260 Infinity autosampler with reliable injections from 0.1 to 100 µL (G1329B) and Agilent 1260 Infinity UV detector (G1321C). Data was recorded and analyzed with Agilent OpenLAB CDS Chemstation Edition software. HANNA pH 211 Microprocessor pH meter with double junction glass electrode was used to adjust the pH. The column used in HPLC was ZORBAX Eclipse plus C18 (150 x 4.6 mm, 3.5µm). Mobile phase consisted of acetonitrile (ACN)/ Phosphate buffer, pH 6.32 with different ratios, flow rate of 1.5 mL/min and the run time was set as double the elution time of each compound. Their purity was recorded with a high percentage (≥95).

Table S2. HPLC purity of target compounds **(4a-l)**

**Compound**	**Formula**	**Retention time (minutes)**	**Purity (%)**
**4a**	$C_{27}H_{22}N_2O_4S$	7.38	99.90
**4b**	$C_{29}H_{26}N_2O_4S$	6.74	95.77
**4c**	$C_{29}H_{26}N_2O_4S$	5.16	96.40
**4d**	$C_{27}H_{21}ClN_2O_4S$	5.08	99.26
**4e**	$C_{29}H_{25}ClN_2O_4S$	7.43	99.40
**4f**	$C_{29}H_{25}ClN_2O_4S$	7.16	98.37
**4g**	$C_{27}H_{21}ClN_2O_4S$	5.32	97.52
**4h**	$C_{29}H_{25}ClN_2O_4S$	6.00	98.49
**4i**	$C_{29}H_{25}ClN_2O_4S$	6.44	98.41
**4j**	$C_{27}H_{20}Cl_2N_2O_4S$	5.04	99.28
**4k**	$C_{29}H_{24}Cl_2N_2O_4S$	6.70	98.54
**4l**	$C_{29}H_{24}Cl_2N_2O_4S$	15.23	99.84

Area Percent Report

Data file:	C:\CHEM32\1\DATA\ANWAR\4A-003.D		
Sample name:	4a		
Description:	Solvent: DMSO + ACN, Flow rate: 1.5 mL/min<br>Mobile phase: 51% ACN + 49% Phosphate buffer		
Sample amount:	2.500	Sample type:	Sample
Instrument:	HPLC	Location:	Vial 51
Injection date:	4/26/2024 11:40:14 PM	Injection:	1 of 1
Acq. method:	ANWAR.M	Injection volume:	5.000
Analysis method:	ANWAR.M	Acq. operator:	SYSTEM
Last changed:	4/27/2024 12:01:31 AM<br>(modified after loading)		

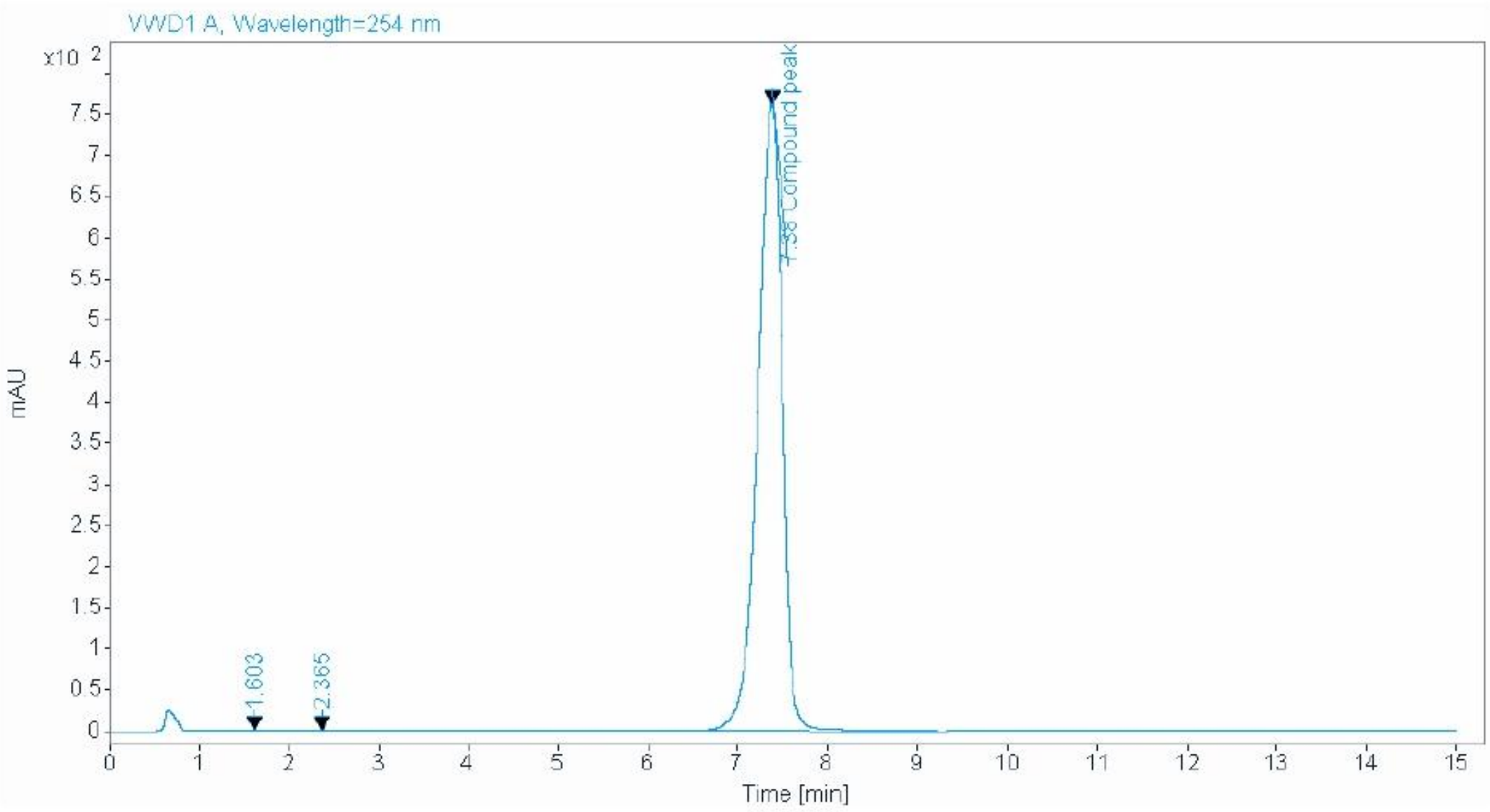

Signal: VWD1 A, Wavelength=254 nm

RT [min]	Type	Width [min]	Area	Height	Area%	Name
1.603	BB	0.0834	6.8272	1.1823	0.0472	
2.365	VB	0.0798	7.4617	1.4561	0.0516	
7.380	VB	0.2973	14454.7930	762.0634	99.9012	Compound peak
		Sum	14469.0819			

Figure S43. HPLC chromatogram of compound **4a**

Area Percent Report

Agilent Technologies

Data file:	C:\CHEM32\1\DATA\ANWAR\4B-004.D		
Sample name:	4b		
Description:	Solvent: DMSO + ACN, Flow rate: 1.5 mL/min<br>Mobile phase: 60% ACN + 40% Phosphate buffer		
Sample amount:	2.500	Sample type:	Sample
Instrument:	HPLC	Location:	Vial 52
Injection date:	4/27/2024 12:05:47 AM	Injection:	1 of 1
Acq. method:	ANWAR.M	Injection volume:	5.000
Analysis method:	ANWAR.M	Acq. operator:	SYSTEM
Last changed:	4/23/2024 7:36:37 AM		

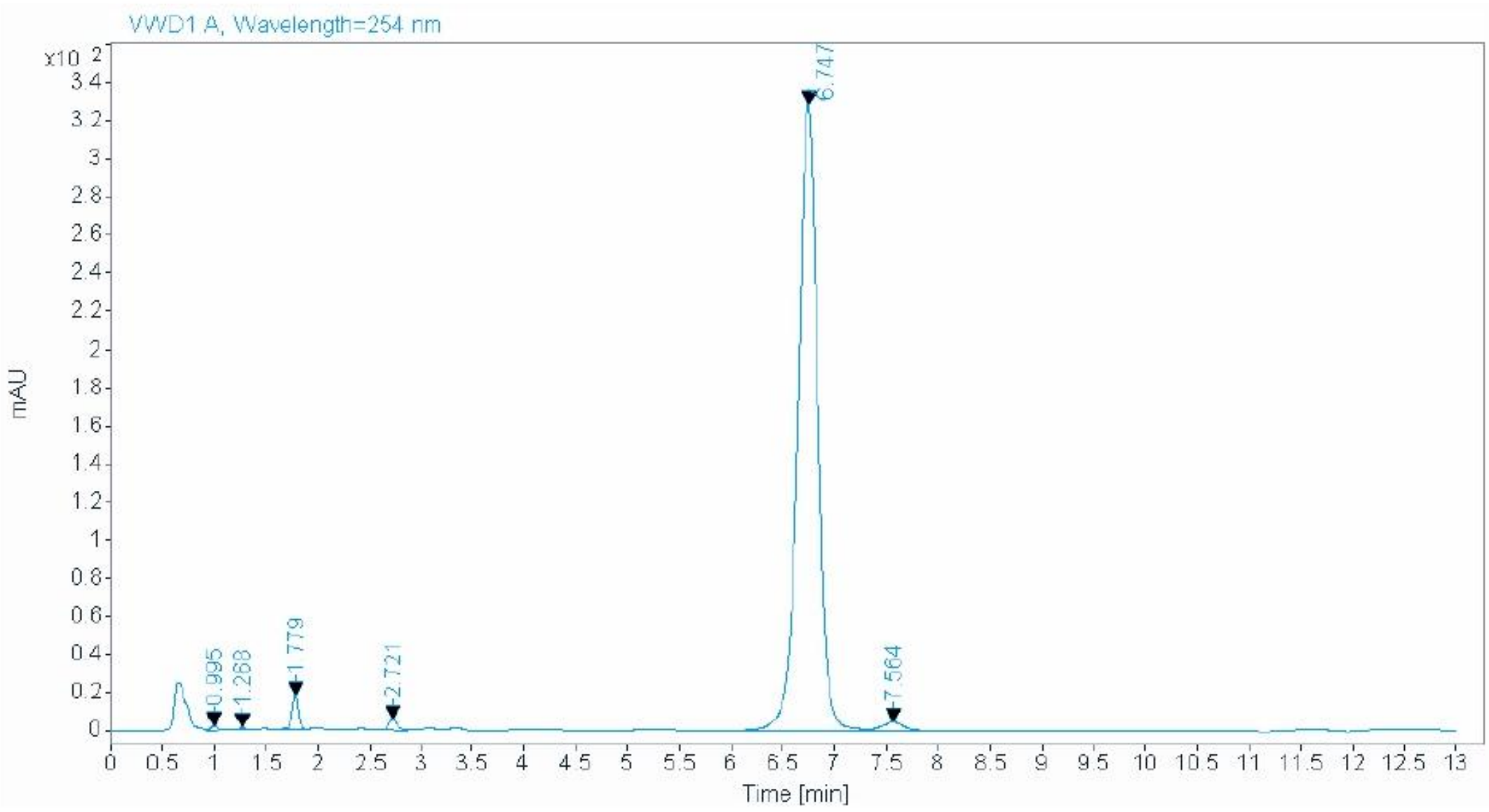

Signal: VWD1 A, Wavelength=254 nm

RT [min]	Type	Width [min]	Area	Height	Area%	Name
0.995	VB	0.0668	9.6734	2.1389	0.2123	
1.268	BB	0.0563	4.3843	1.1537	0.0962	
1.779	VV	0.0667	77.3652	17.7967	1.6976	
2.721	BV	0.0890	32.9812	5.5801	0.7237	
6.747	BV	0.2029	4365.0630	328.7443	95.7799	
7.564	VB	0.2372	67.9204	4.2795	1.4903	
		Sum	4557.3876			

Figure S44. HPLC chromatogram of compound **4b**

Area Percent Report

Agilent Technologies

Data file:	C:\CHEM32\1\DATA\ANWAR\4C-005.D		
Sample name:	4c		
Description:	Solvent: DMSO + ACN, Flow rate: 1.5 mL/min<br>Mobile phase: 60% ACN + 40% Phosphate buffer		
Sample amount:	2.500	Sample type:	Sample
Instrument:	HPLC	Location:	Vial 53
Injection date:	4/27/2024 12:20:32 AM	Injection:	1 of 1
Acq. method:	ANWAR.M	Injection volume:	5.000
Analysis method:	ANWAR.M	Acq. operator:	SYSTEM
Last changed:	4/27/2024 12:33:10 AM<br>(modified after loading)		

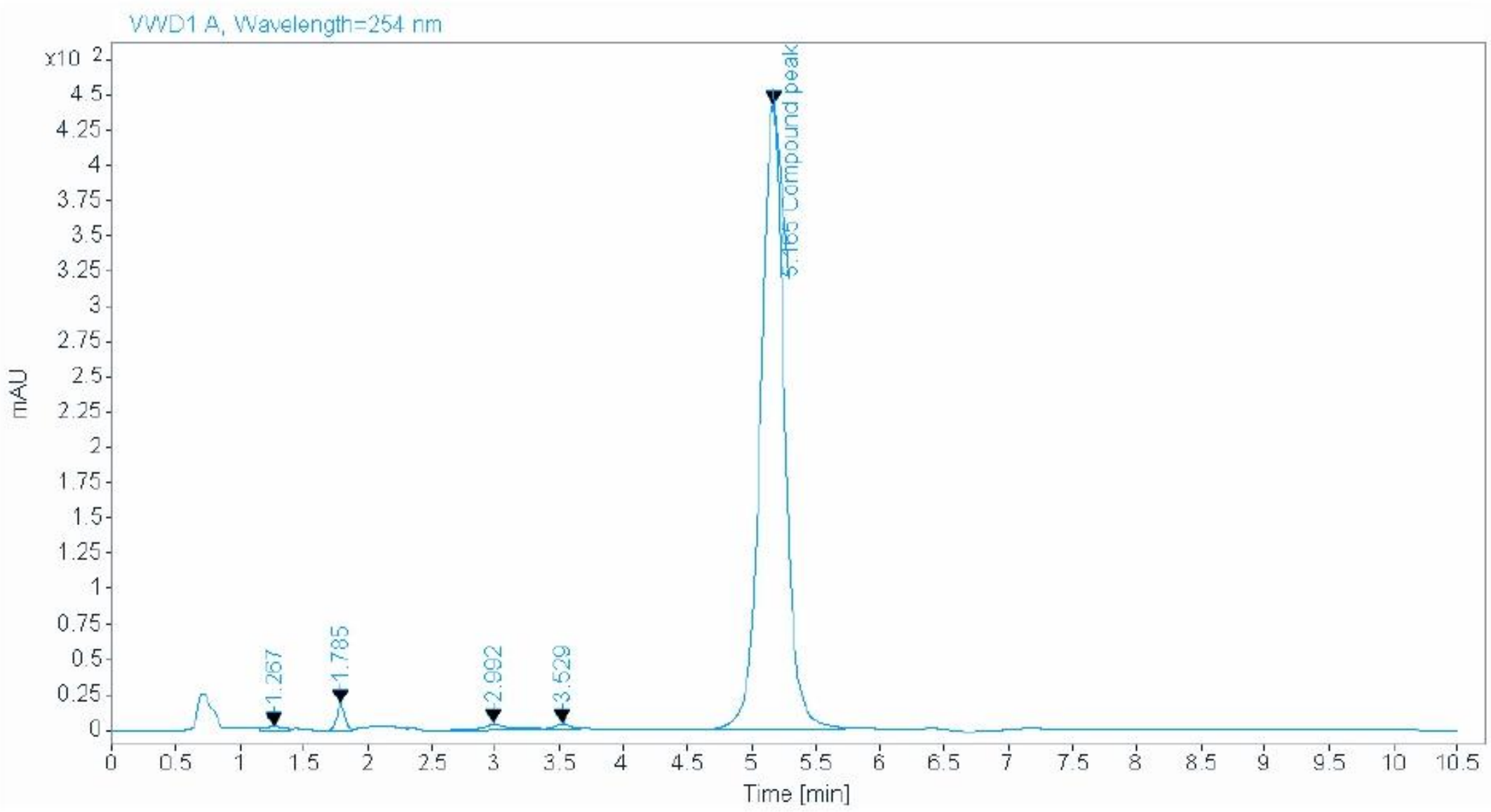

Signal: VWD1 A, Wavelength=254 nm

RT [min]	Type	Width [min]	Area	Height	Area%	Name
1.267	VV	0.1291	33.2903	3.3162	0.5593	
1.785	BV	0.0676	85.5028	19.3174	1.4366	
2.992	BV	0.2051	62.9120	4.0093	1.0570	
3.529	VV	0.1329	32.3316	3.6663	0.5432	
5.165	BV	0.1993	5737.7798	442.4784	96.4038	Compound peak
		Sum	5951.8165			

Figure S45. HPLC chromatogram of compound **4c**

Area Percent Report

Agilent Technologies

**Data file:**	C:\CHEM32\1\DATA\ANWAR\4D-006.D		
**Sample name:**	4d		
**Description:**	Solvent: DMSO + ACN, Flow rate: 1.5 mL/min<br>Mobile phase: 60% ACN + 40% Phosphate buffer		
**Sample amount:**	2.500	**Sample type:**	Sample
**Instrument:**	HPLC	**Location:**	Vial 61
**Injection date:**	4/27/2024 12:32:46 AM	**Injection:**	1 of 1
**Acq. method:**	ANWAR.M	**Injection volume:**	5.000
**Analysis method:**	ANWAR.M	**Acq. operator:**	SYSTEM
**Last changed:**	4/27/2024 12:45:51 AM<br>(modified after loading)		

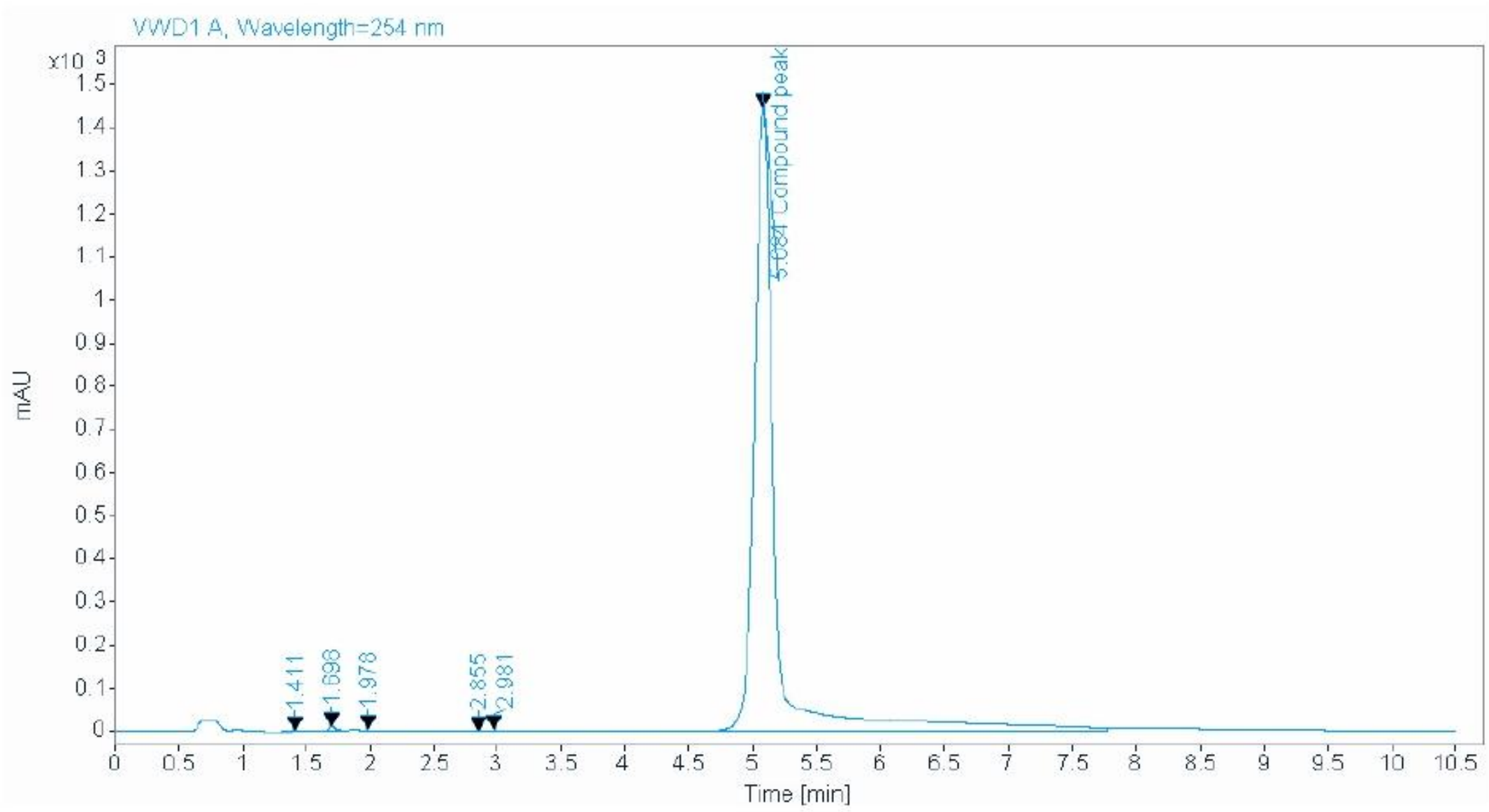

Signal: VWD1 A, Wavelength=254 nm

RT [min]	Type	Width [min]	Area	Height	Area%	Name
1.411	BB	0.0748	14.4968	2.7810	0.0854	
1.698	BV	0.0713	59.8810	12.1993	0.3527	
1.978	VB	0.1670	31.6257	2.6934	0.1863	
2.855	BV	0.0855	8.7633	1.5619	0.0516	
2.981	VB	0.0827	9.9151	1.8461	0.0584	
5.084	BV	0.1720	16854.6738	1446.0203	99.2657	Compound peak
		Sum	16979.3557			

Figure S46. HPLC chromatogram of compound **4d**

Area Percent Report

Agilent Technologies

Data file:	C:\CHEM32\1\DATA\ANWAR\4E-010.D		
Sample name:	4e		
Description:	Solvent: DMSO + ACN, Flow rate: 1.5 mL/min<br>Mobile phase: 65% ACN + 35% Phosphate buffer		
Sample amount:	2.500	Sample type:	Sample
Instrument:	HPLC	Location:	Vial 62
Injection date:	4/27/2024 2:36:32 AM	Injection:	1 of 1
Acq. method:	ANWAR.M	Injection volume:	5.000
Analysis method:	ANWAR.M	Acq. operator:	SYSTEM
Last changed:	4/27/2024 2:55:51 AM<br>(modified after loading)		

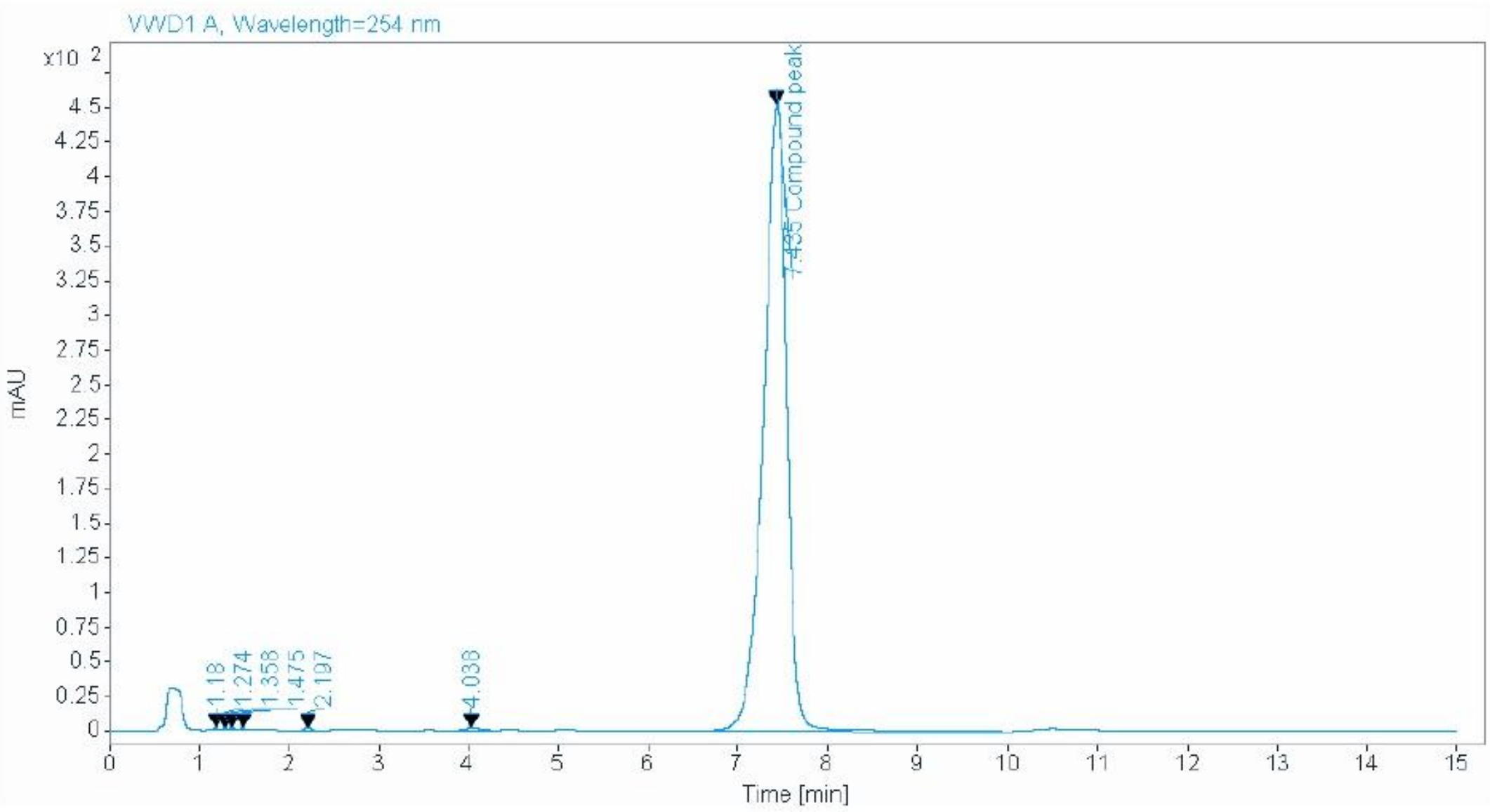

Signal: VWD1 A, Wavelength=254 nm

RT [min]	Type	Width [min]	Area	Height	Area%	Name
1.180	BB	0.0450	4.4353	1.5666	0.0542	
1.274	BV	0.0491	2.2912	0.7618	0.0280	
1.358	VV	0.0636	3.7698	0.9249	0.0461	
1.475	VB	0.0679	2.9040	0.7073	0.0355	
2.197	BB	0.0797	11.0363	2.2298	0.1350	
4.038	VV	0.1642	23.9199	2.1105	0.2925	
7.435	VB	0.2720	8128.6382	451.6813	99.4086	Compound peak
		Sum	8176.9946			

Figure S47. HPLC chromatogram of compound **4e**

Area Percent Report

Agilent Technologies

**Data file:**	C:\CHEM32\1\DATA\ANWAR\4F-009.D		
**Sample name:**	4f		
**Description:**	Solvent: DMSO + ACN, Flow rate: 1.5 mL/min<br>Mobile phase: 65% ACN + 35% Phosphate buffer		
**Sample amount:**	2.500	**Sample type:**	Sample
**Instrument:**	HPLC	**Location:**	Vial 63
**Injection date:**	4/27/2024 2:20:14 AM	**Injection:**	1 of 1
**Acq. method:**	ANWAR.M	**Injection volume:**	5.000
**Analysis method:**	ANWAR.M	**Acq. operator:**	SYSTEM
**Last changed:**	4/27/2024 1:40:50 AM		

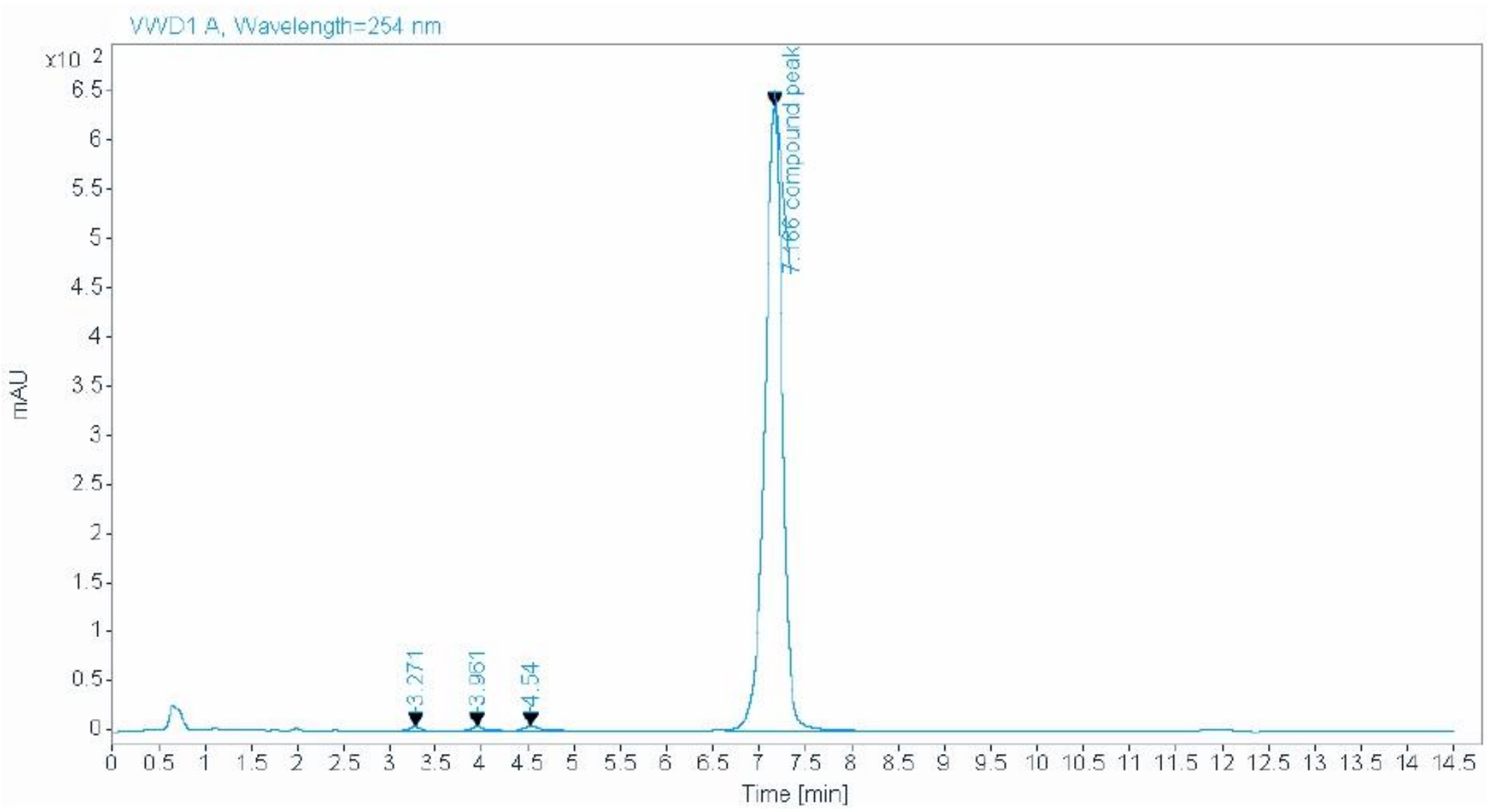

Signal: VWD1 A, Wavelength=254 nm

RT [min]	Type	Width [min]	Area	Height	Area%	Name
3.271	VB	0.1327	34.8678	3.8859	0.4081	
3.961	BB	0.1636	42.1835	3.7411	0.4937	
4.540	BV	0.1970	62.0650	4.6703	0.7264	
7.166	VB	0.2025	8404.5771	634.6093	98.3717	compound peak
		Sum	8543.6935			

Figure S48. HPLC chromatogram of compound **4f**

Area Percent Report

Agilent Technologies

**Data file:**	C:\CHEM32\1\DATA\ANWAR\4G-020.D		
**Sample name:**	4g		
**Description:**	Solvent: DMSO + ACN, Flow rate: 1.5 mL/min□<br>Mobile phase: 50% ACN + 50% Phosphate buffer		
**Sample amount:**	2.500	**Sample type:**	Sample
**Instrument:**	HPLC	**Location:**	Vial 71
**Injection date:**	4/27/2024 4:27:29 AM	**Injection:**	1 of 1
**Acq. method:**	ANWAR.M	**Injection volume:**	5.000
**Analysis method:**	ANWAR.M	**Acq. operator:**	SYSTEM
**Last changed:**	4/27/2024 5:20:30 AM<br>(modified after loading)		

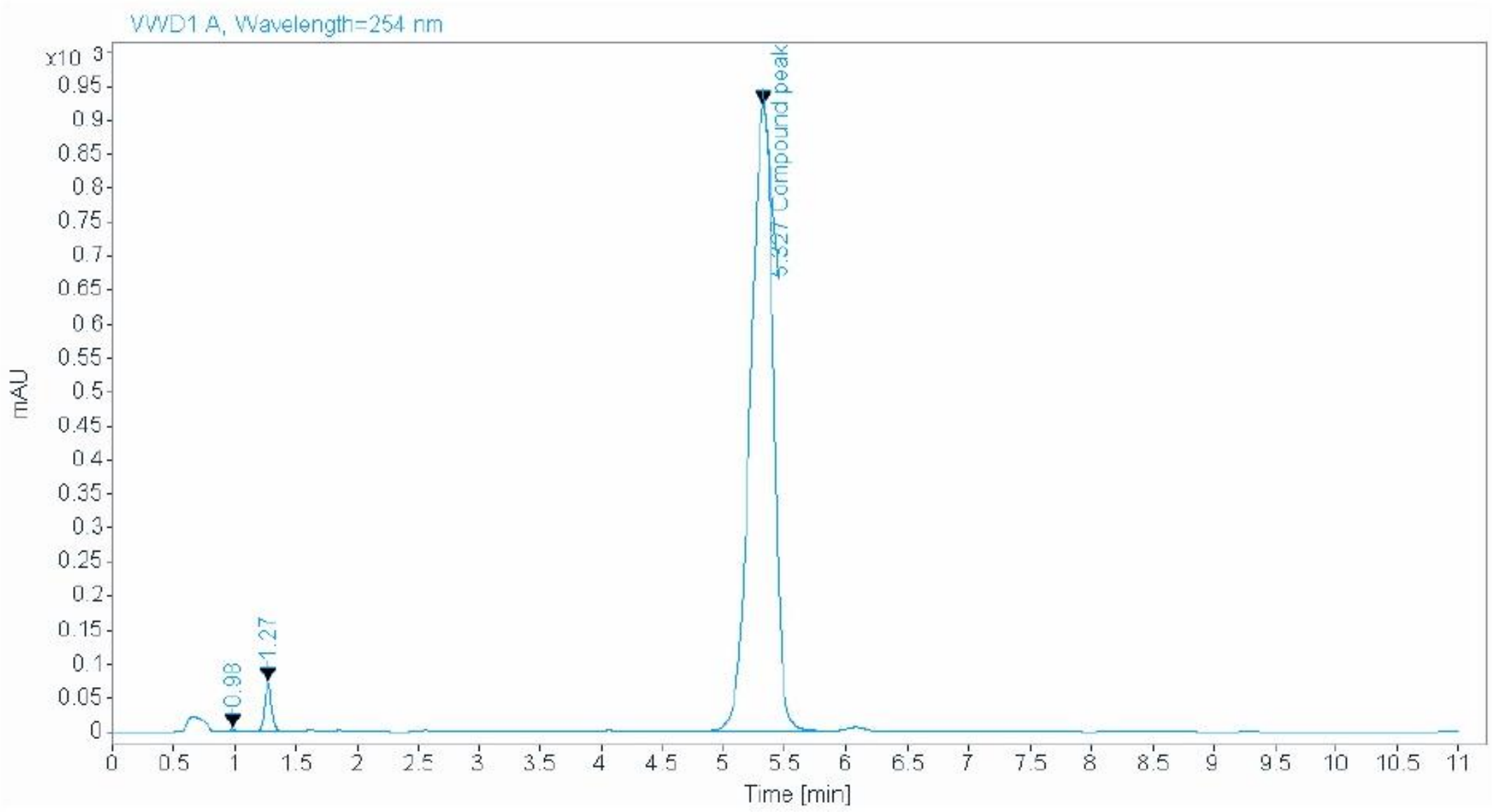

Signal: VWD1 A, Wavelength=254 nm

RT [min]	Type	Width [min]	Area	Height	Area%	Name
0.980	VB	0.0648	18.6408	4.2850	0.1502	
1.270	BB	0.0614	288.5186	74.1845	2.3247	
5.327	VV	0.2072	12104.0615	921.7103	97.5251	Compound peak
		Sum	12411.2208			

Figure S49. HPLC chromatogram of compound **4g**

Area Percent Report

Agilent Technologies

**Data file:**	C:\CHEM32\1\DATA\ANWAR\4H-011.D		
**Sample name:**	4h		
**Description:**	Solvent: DMSO + ACN, Flow rate: 1.5 mL/min<br>Mobile phase: 65% ACN + 35% Phosphate buffer		
**Sample amount:**	2.500	**Sample type:**	Sample
**Instrument:**	HPLC	**Location:**	Vial 72
**Injection date:**	4/27/2024 2:54:49 AM	**Injection:**	1 of 1
**Acq. method:**	ANWAR.M	**Injection volume:**	5.000
**Analysis method:**	ANWAR.M	**Acq. operator:**	SYSTEM
**Last changed:**	4/27/2024 3:08:27 AM<br>(modified after loading)		

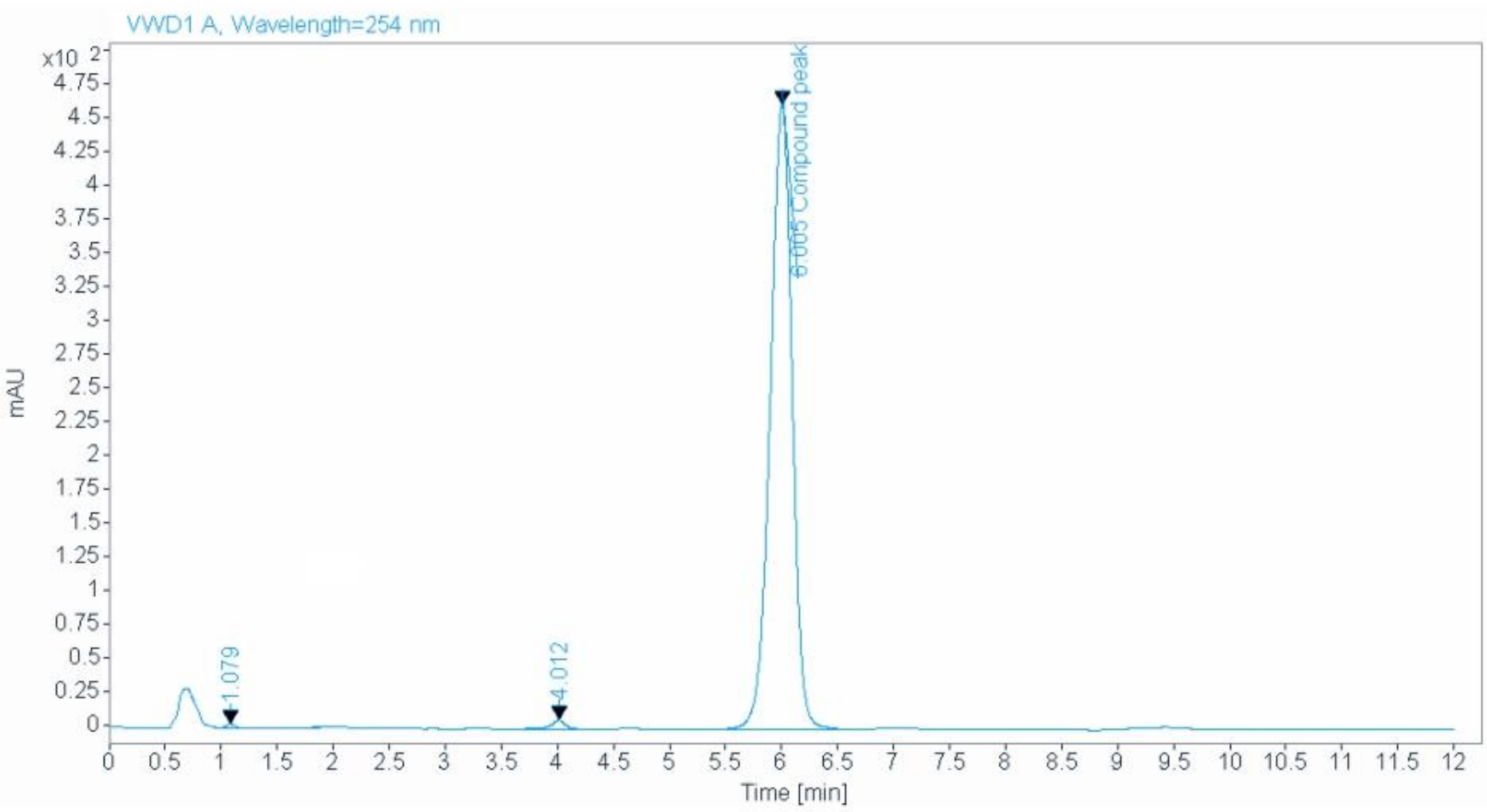

Signal: VWD1 A, Wavelength=254 nm

RT [min]	Type	Width [min]	Area	Height	Area%	Name
1.079	VV	0.1044	22.8440	3.3132	0.3572	
4.012	BV	0.1519	73.1181	6.8850	1.1433	
6.005	BB	0.2111	6299.1704	461.8958	98.4995	Compound peak
		Sum	6395.1325			

Figure S50. HPLC chromatogram of compound **4h**

Area Percent Report

Agilent Technologies

**Data file:**	C:\CHEM32\1\DATA\ANWAR\4I-013.D		
**Sample name:**	4i		
**Description:**	Solvent: DMSO + ACN, Flow rate: 1.5 mL/min<br>Mobile phase: 65% ACN + 35% Phosphate buffer		
**Sample amount:**	2.500	**Sample type:**	Sample
**Instrument:**	HPLC	**Location:**	Vial 73
**Injection date:**	4/27/2024 3:17:53 AM	**Injection:**	1 of 1
**Acq. method:**	ANWAR.M	**Injection volume:**	5.000
**Analysis method:**	ANWAR.M	**Acq. operator:**	SYSTEM
**Last changed:**	4/27/2024 3:33:43 AM<br>(modified after loading)		

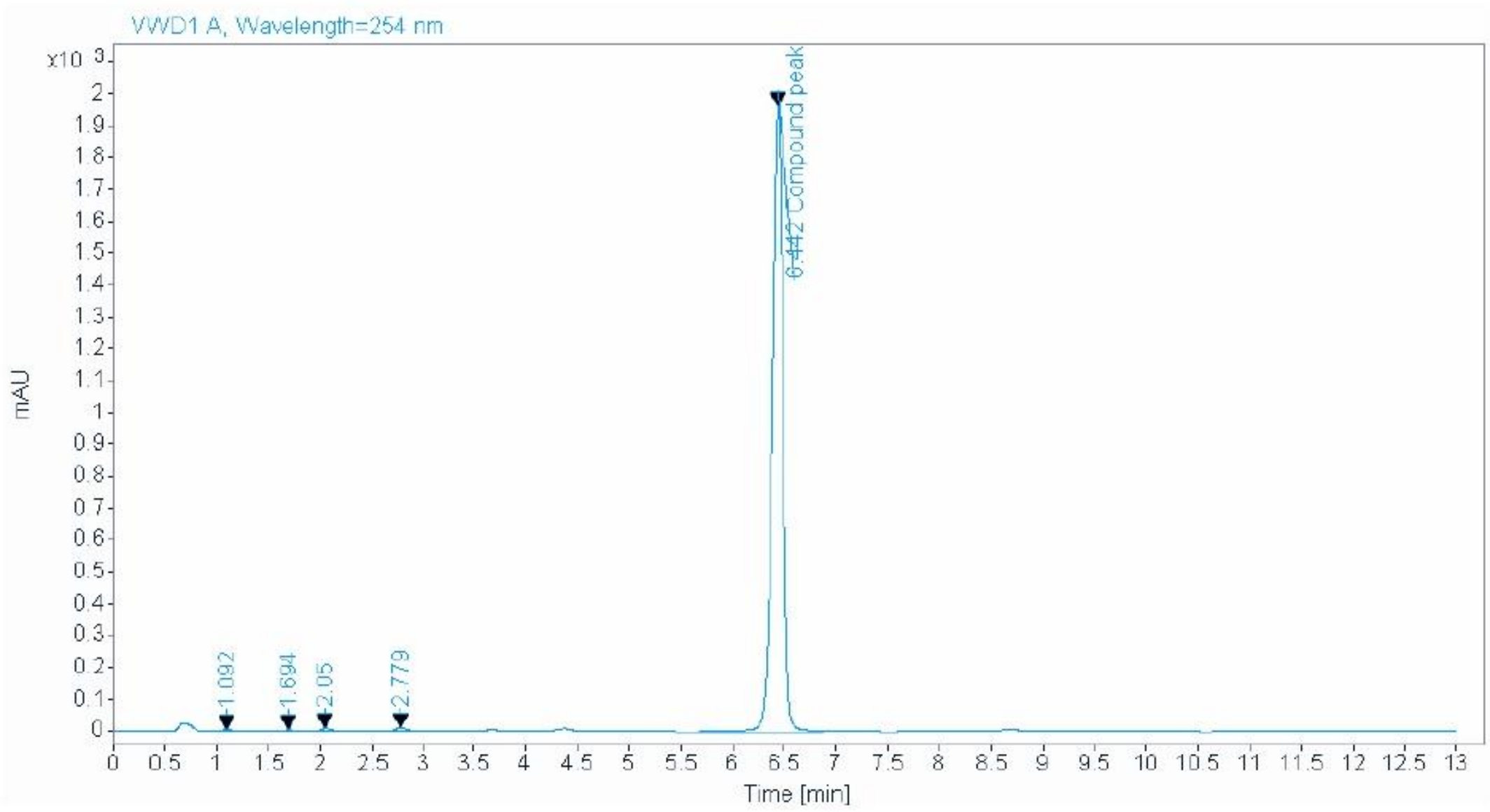

Signal: VWD1 A, Wavelength=254 nm

RT [min]	Type	Width [min]	Area	Height	Area%	Name
1.092	BB	0.0681	30.3048	6.7821	0.2172	
1.694	VV	0.1207	33.0264	3.9826	0.2367	
2.050	VB	0.0918	63.0008	9.9587	0.4515	
2.779	VB	0.1162	94.4464	12.4966	0.6768	
6.442	BV	0.1098	13734.2920	1959.7448	98.4179	Compound peak
		Sum	13955.0705			

Figure S51. HPLC chromatogram of compound **4i**

Area Percent Report

Agilent Technologies

**Data file:**	C:\CHEM32\1\DATA\ANWAR\4J-017.D		
**Sample name:**	4j		
**Description:**	Solvent: DMSO + ACN, Flow rate: 1.5 mL/min<br>Mobile phase: 60% ACN + 40% Phosphate buffer		
**Sample amount:**	2.500	**Sample type:**	Sample
**Instrument:**	HPLC	**Location:**	Vial 81
**Injection date:**	4/27/2024 4:01:35 AM	**Injection:**	1 of 1
**Acq. method:**	ANWAR.M	**Injection volume:**	5.000
**Analysis method:**	ANWAR.M	**Acq. operator:**	SYSTEM
**Last changed:**	4/27/2024 4:13:43 AM<br>(modified after loading)		

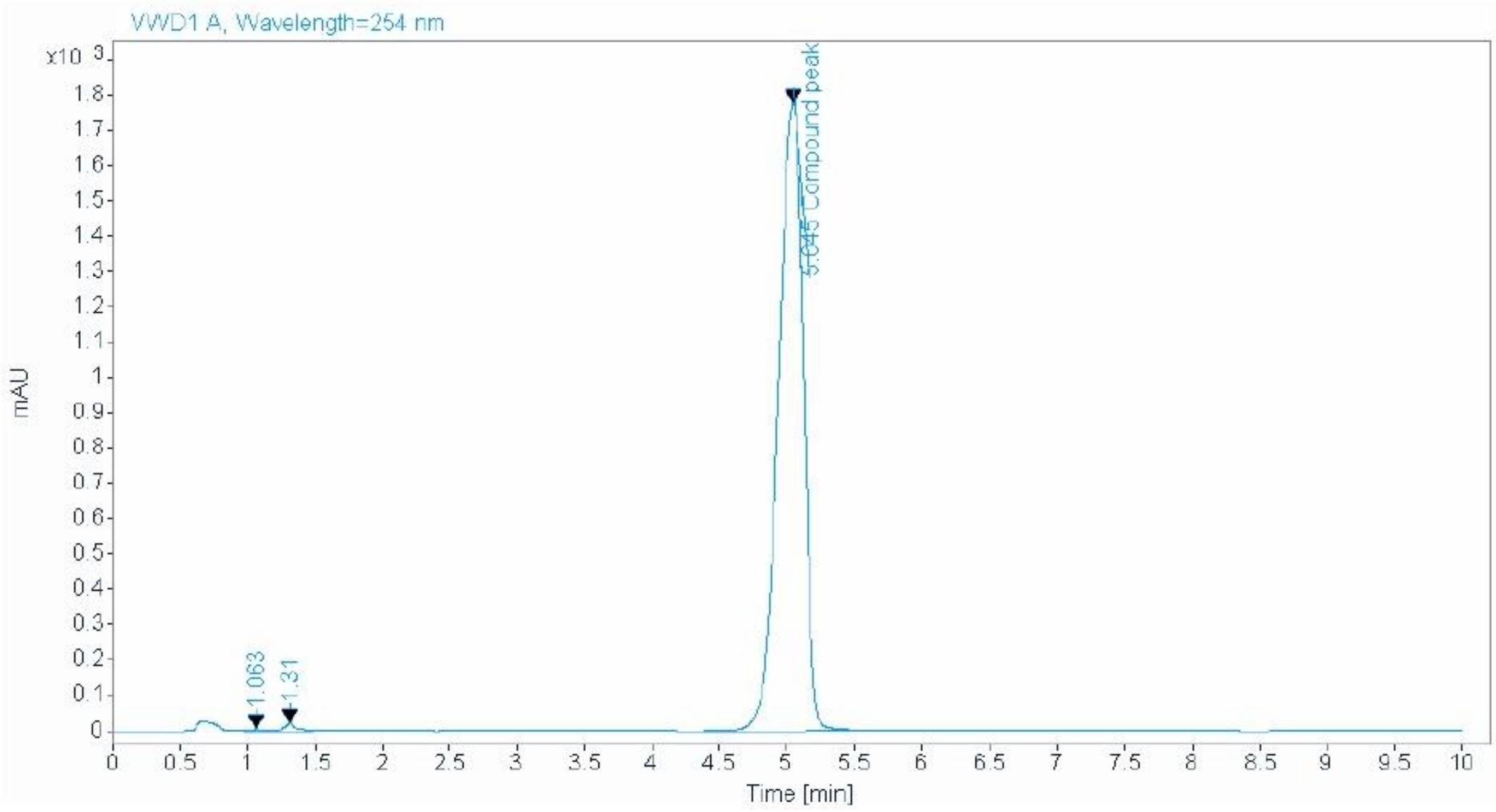

Signal: VWD1 A, Wavelength=254 nm

RT [min]	Type	Width [min]	Area	Height	Area%	Name
1.063	VV	0.1035	29.4262	4.3158	0.1273	
1.310	VB	0.0904	136.9560	21.4785	0.5923	
5.045	BB	0.2049	22955.2051	1774.5774	99.2804	Compound peak
		Sum	23121.5873			

Figure S52. HPLC chromatogram of compound **4j**

Area Percent Report

Agilent Technologies

Data file:	C:\CHEM32\1\DATA\ANWAR\4K-024.D		
Sample name:	4k		
Description:	Solvent: DMSO + ACN, Flow rate: 1.5 mL/min<br>Mobile phase: 70% ACN + 30% Phosphate buffer		
Sample amount:	2.500	Sample type:	Sample
Instrument:	HPLC	Location:	Vial 82
Injection date:	4/27/2024 5:58:00 AM	Injection:	1 of 1
Acq. method:	ANWAR.M	Injection volume:	5.000
Analysis method:	ANWAR.M	Acq. operator:	SYSTEM
Last changed:	4/27/2024 6:13:56 AM<br>(modified after loading)		

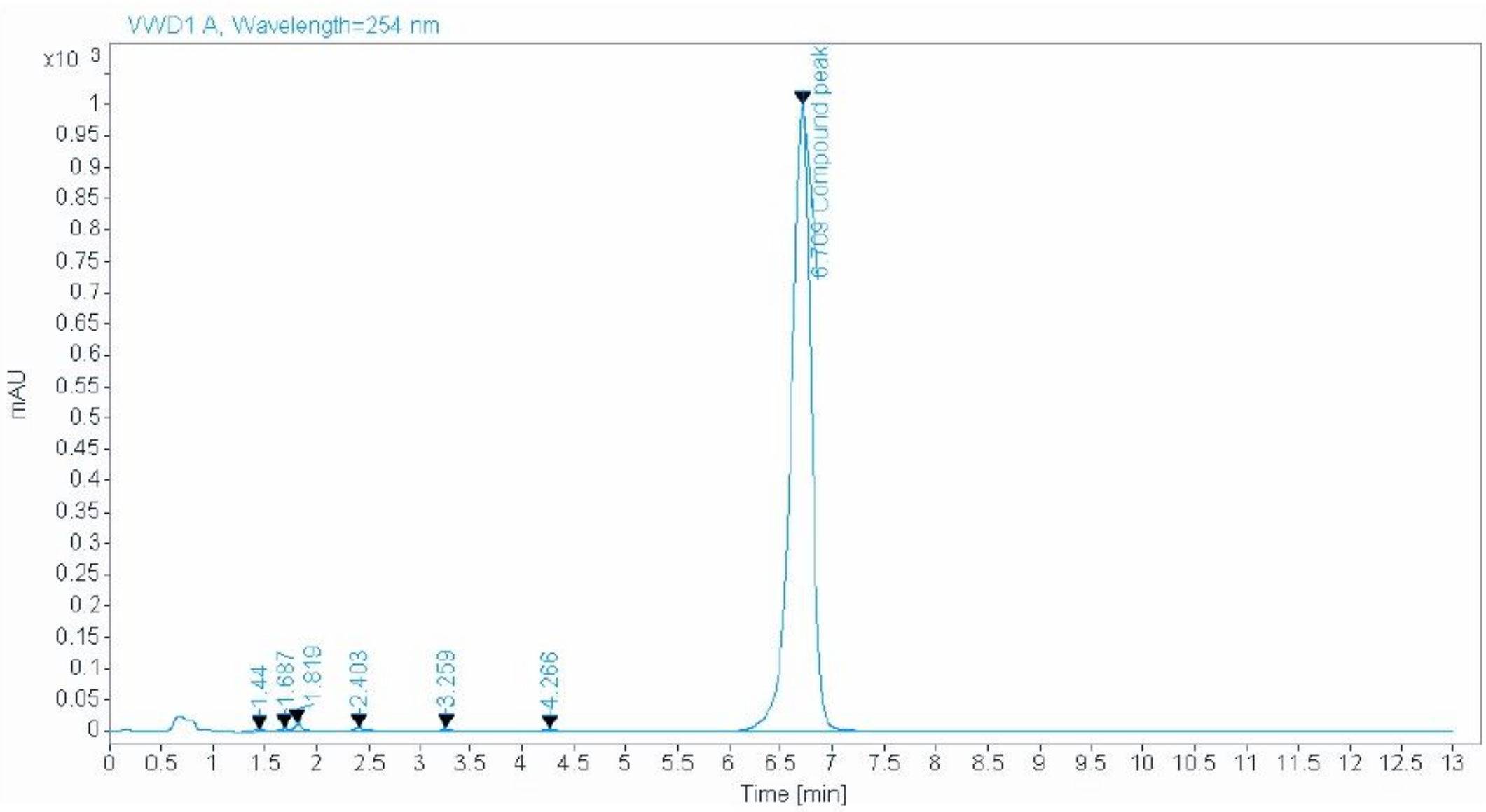

Signal: VWD1 A, Wavelength=254 nm

RT [min]	Type	Width [min]	Area	Height	Area%	Name
1.440	BB	0.1290	17.5351	1.9859	0.1276	
1.687	BV	0.0855	16.6348	2.9640	0.1210	
1.819	VB	0.0722	49.3596	10.2551	0.3592	
2.403	VB	0.1364	57.4918	5.5731	0.4184	
3.259	BV	0.1286	37.5319	3.9574	0.2731	
4.266	BV	0.1099	22.0118	2.9897	0.1602	
6.709	BB	0.2064	13541.7773	997.3005	98.5405	Compound peak
		Sum	13742.3423			

Figure S53. HPLC chromatogram of compound **4k**

Area Percent Report

**Data file:**	C:\CHEM32\1\DATA\ANWAR\4L-023.D		
**Sample name:**	4l		
**Description:**	Solvent: DMSO + ACN, Flow rate: 1.5 mL/min<br>Mobile phase: 60% ACN + 40% Phosphate buffer		
**Sample amount:**	2.500	**Sample type:**	Sample
**Instrument:**	HPLC	**Location:**	Vial 83
**Injection date:**	4/27/2024 5:18:46 AM	**Injection:**	1 of 1
**Acq. method:**	ANWAR.M	**Injection volume:**	5.000
**Analysis method:**	ANWAR.M	**Acq. operator:**	SYSTEM
**Last changed:**	4/27/2024 5:55:33 AM<br>(modified after loading)		

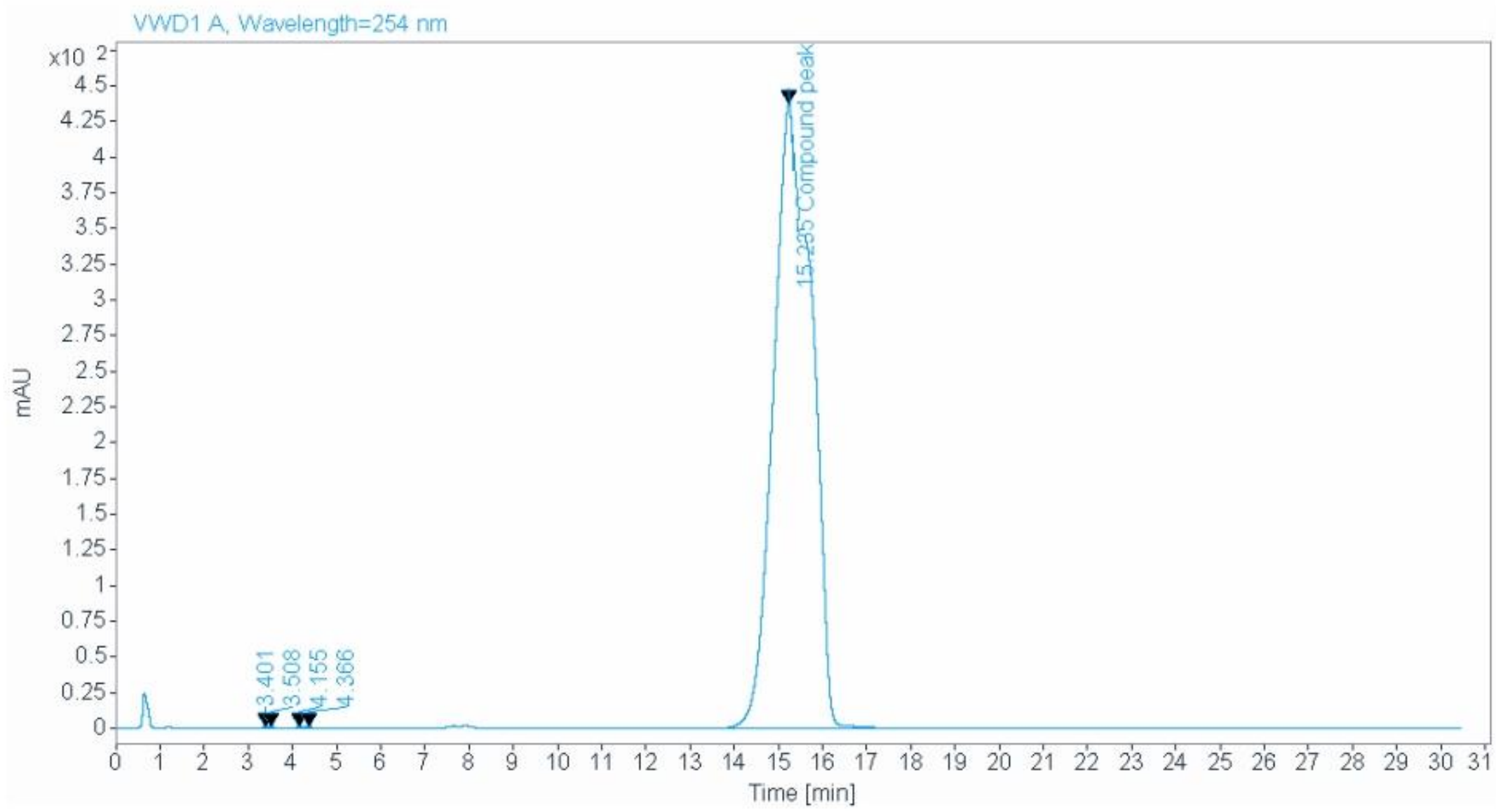

Signal: VWD1 A, Wavelength=254 nm

RT [min]	Type	Width [min]	Area	Height	Area%	Name
3.401	BV	0.1112	9.3620	1.2814	0.0357	
3.508	VB	0.1283	11.2454	1.3084	0.0429	
4.155	BV	0.1405	8.7635	0.9254	0.0334	
4.366	VV	0.1717	12.4623	1.0878	0.0476	
15.235	MM	0.9971	26161.7207	437.3030	99.8404	Compound peak
		Sum	26203.5538			

Figure S54. HPLC chromatogram of compound **4l**

General steps for the preparation of 2-mercaptoquinazolin-4(3*H*)-one derivatives (1a-d)

To synthesize intermediate (**1a**), a mixture of anthranilic acid (1371 mg, 10 mmol) and phenyl isothiocyanate (1487 mg, 11 mmol) in absolute ethanol (50 mL) containing TEA (1113 mg, 11 mmol) was heated under reflux at 80 °C for 24 h. The reaction mixture was filtered while hot. The solid obtained and left to cool at room temp. then washed with absolute ethanol, then diethyl ether, and vacuum-dried to furnish compound (**1a**).

2-Mercapto-3-phenylquinazolin-4(3*H*)-one (1a). Off-white powder (81%). Mp: > 300 °C [45]. Anal. calcd for $C_{14}H_{10}N_2OS$: C, 66.12; H, 3.96; N, 11.02; S, 12.61. Found: C, 66.25; H, 3.97; N, 10.94; S, 12.55%.

7-Chloro-2-mercapto-3-phenylquinazolin-4(3*H*)-one (**1b**). Compound (**1b**) was prepared following the procedure adopted for (**1a**), employing 4-chloroanthranilic acid and phenyl isothiocyanate. Off-white powder (55%). Mp: > 300 °C [46]. Anal. calcd for $C_{14}H_9ClN_2OS$: C, 58.24; H, 3.14; N, 9.70; S, 11.10. Found: C, 58.01; H, 3.11; N, 9.70; S, 11.01%.

3-(4-Chlorophenyl)-2-mercaptoquinazolin-4(3*H*)-one (1c). Compound (**1c**) was prepared following the procedure adopted for (**1a**), employing anthranilic acid and 4-chlorophenyl isothiocyanate. Off-white powder (87%). Mp: > 300 °C [47]. Anal. calcd for $C_{14}H_9ClN_2OS$: C, 58.24; H, 3.14; N, 9.70; S, 11.10. Found: C, 58.49; H, 3.15; N, 9.56; S, 11.14%.

7-Chloro-3-(4-chlorophenyl)-2-mercaptoquinazolin-4(3*H*)-one (1d). Compound (**1d**) was prepared following the procedure adopted for (**1a**), employing 4-chloroanthranilic acid and 4-chlorophenyl isothiocyanate. Off-white powder (66%). Mp: > 300 °C [46]. Anal. calcd for $C_{14}H_8Cl_2N_2OS$: C, 52.03; H, 2.50; N, 8.67; S, 9.92. Found: C, 51.89; H, 2.49; N, 8.59; S, 9.99%.

General steps for the preparation of 7-hydroxy-2*H*-chromen-2-one derivatives (2a-c)

To synthesize intermediate (**2a**), conc. H_2SO_4 (98.0%) (0.5 mL) was cooled to 5 °C. Resorcinol (500 mg, 4.54 mmol) was dissolved in ethyl acetoacetate (670 mg, 5.14 mmol), and the mixture was added dropwise to chilled conc. H_2SO_4 (98.0%) while stirring for 30 min. After the addition was complete, the stirring continued at 25 °C for 24 h. The reaction mixture was then poured onto ice-cold water while stirring. The mixture was left overnight, then neutralized with conc. NaOH solution (1.5 g NaOH / 15.0 mL distilled water) until pH~8. After neutralization, the mixture was filtered. The solid obtained was washed with distilled water several times, then with absolute methanol and diethyl ether, and vacuum-dried to furnish compound (**2a**).

7-Hydroxy-4-methyl-2*H*-chromen-2-one (2a)

White powder (64%). Mp: 187 °C [48]. ^{1}H NMR (500 MHz, DMSO-d_6) δ (ppm): 2.31 (3H, s, CH_3), 6.08 (1H, s, 3-H$_{coumarin}$), 6.65 (1H, s, 6-H$_{coumarin}$), 6.74 (1H, s, 8-H$_{coumarin}$), 7.55 (1H, s, 5-H$_{coumarin}$), 10.52 (1H, s, OH). Anal. calcd for $C_{10}H_8O_3$: C, 68.18; H, 4.58. Found: C, 68.34; H, 4.61%.

7-Hydroxy-4-propyl-2*H*-chromen-2-one (2b). Compound (**2b**) was prepared following the procedure adopted for (**2a**), employing ethyl butyrylacetate. Brown powder (75%). Mp: 138 °C [49]. Anal. calcd for $C_{12}H_{12}O_3$: C, 70.58; H, 5.92. Found: C, 70.77; H, 5.85%.

7-Hydroxy-4-isopropyl-2*H*-chromen-2-one (2c). Compound (**2c**) was prepared following the procedure adopted

for (**2a**), employing ethyl isobutyrylacetate. Brown powder (66%). Mp: 68 °C [50]. Anal. calcd for $C_{12}H_{12}O_3$: C, 70.58; H, 5.92. Found: C, 70.75; H, 5.89%.

General steps for the preparation of 7-(3-chloropropoxy)-2*H*-chromen-2-one derivatives (3a-c)

To synthesize intermediate (**3a**), a mixture of 7-hydroxy-4-methyl-2*H*-chromen-2-one (**2a**) (528 mg, 3 mmol), anhydrous potassium carbonate (4560 mg, 33 mmol), and 1-bromo-3-chloropropane (472 mg, 3 mmol) was heated under reflux in dry acetone (50 mL) at 60 °C for 48 h. The reaction mixture was filtered while hot. The filtrate was concentrated under reduced pressure to a residue, then petroleum ether was added to the residue, and the mixture was kept in the freezer for 1 h. The solid formed was filtered off and vacuum-dried to furnish compound (**3a**).

7-(3-Chloropropoxy)-4-methyl-2*H*-chromen-2-one (3a). White powder (72%). Mp: 83 °C [51]. Anal. calcd for $C_{13}H_{13}ClO_3$: C, 61.79; H, 5.19. Found: C, 61.49; H, 5.23%.

7-(3-Chloropropoxy)-4-propyl-2*H*-chromen-2-one (3b)

Compound (**3b**) was prepared following the procedure adopted for (**3a**), employing 7-hydroxy-4-propyl-2*H*-chromen-2-one (**2b**). Yellow powder (85%). Mp: 70 °C. ^{1}H NMR (500 MHz, DMSO-d_6) δ (ppm): 0.92 (3H, t, J = 7.5 Hz, $CH_2CH_2\mathbf{CH_3}$), 1.58 (2H, sext, J = 7.5 Hz, $CH_2\mathbf{CH_2}CH_3$), 2.15 (2H, quint, J = 6.5 Hz, $OCH_2\mathbf{CH_2}CH_2Cl$), 2.69 (2H, t, J = 7.5 Hz, $\mathbf{CH_2}CH_2CH_3$), 3.76 (2H, t, J = 6.5 Hz, $OCH_2CH_2\mathbf{CH_2}Cl$), 4.15 (2H, t, J = 6.5 Hz, $O\mathbf{CH_2}CH_2CH_2Cl$), 6.12 (1H, s, 3-H $_{coumarin}$), 6.93 (1H, dd, J = 8.5 Hz, J = 2.5 Hz, 6-H $_{coumarin}$), 6.97 (1H, d, J = 2.5 Hz, 8-H $_{coumarin}$), 7.69 (1H, d, J = 8.5 Hz, 5-H $_{coumarin}$). Anal. calcd for $C_{15}H_{17}ClO_3$: C, 64.17; H, 6.10. Found: C, 63.94; H, 6.14%.

7-(3-Chloropropoxy)-4-isopropyl-2*H*-chromen-2-one (3c)

Compound (**3c**) was prepared following the procedure adopted for (**3a**), employing 7-hydroxy-4-isopropyl-2*H*-chromen-2-one (**2c**). Yellow powder (72%). Mp: 69 °C. ^{1}H NMR (500 MHz, DMSO-d_6) δ (ppm): 1.18 (6H, d, J = 7.0 Hz, $CH(\mathbf{CH_3})_2$), 2.15 (2H, quint, J = 6.5 Hz, $OCH_2\mathbf{CH_2}CH_2Cl$), 3.28 (1H, sept, J = 7.0 Hz, $\mathbf{CH}(CH_3)_2$), 3.76 (2H, t, J = 6.5 Hz, $OCH_2CH_2\mathbf{CH_2}Cl$), 4.15 (2H, t, J = 6.5 Hz, $O\mathbf{CH_2}CH_2CH_2Cl$), 6.10 (1H, s, 3-H $_{coumarin}$), 6.93 (1H, d, J = 8.5 Hz, 6-H $_{coumarin}$), 6.96 (1H, s, 8-H $_{coumarin}$), 7.73 (1H, d, J = 8.5 Hz, 5-H $_{coumarin}$). Anal. calcd for $C_{15}H_{17}ClO_3$: C, 64.17; H, 6.10. Found: C, 64.43; H, 6.09%.
